# Synthesis of New Triarylpyrazole Derivatives Possessing Terminal Sulfonamide Moiety and Their Inhibitory Effects on PGE_2_ and Nitric Oxide Productions in Lipopolysaccharide-Induced RAW 264.7 Macrophages

**DOI:** 10.3390/molecules23102556

**Published:** 2018-10-07

**Authors:** Mohammed S. Abdel-Maksoud, Mohammed I. El-Gamal, Mahmoud M. Gamal El-Din, Yunji Choi, Jungseung Choi, Ji-Sun Shin, Shin-Young Kang, Kyung Ho Yoo, Kyung-Tae Lee, Daejin Baek, Chang-Hyun Oh

**Affiliations:** 1 Medicinal & Pharmaceutical Chemistry Department, Pharmaceutical and Drug Industries Research Division, National Research Centre (NRC), Dokki, Giza 12622, Egypt; ph_ss@hotmail.com (M.S.A.-M.); dr.m.g.eldin@hotmail.com (M.M.G.E.-D.); 2 Department of Medicinal Chemistry, College of Pharmacy, University of Sharjah, Sharjah 27272, UAE; drmelgamal2002@gmail.com; 3 Sharjah Institute for Medical Research, University of Sharjah, Sharjah 27272, UAE; 4 Department of Medicinal Chemistry, Faculty of Pharmacy, University of Mansoura, Mansoura 35516, Egypt; 5 Department of Chemistry, Hanseo University, Seosan 31962, Korea; cossmoss@paran.com (Y.C.); djbaek@hanseo.ac.kr (J.C.); 6 Department of Pharmaceutical Biochemistry, College of Pharmacy, Kyung Hee University, Seoul 02792 Korea; jsshin@khu.ac.kr (J.-S.S.); kang940818@naver.com (S.-Y.K.); ktlee@khu.ac.kr (K.-T.L.); 7 Department of Life and Nanopharmaceutical Science, College of Pharmacy, Kyung Hee University, Seoul 130-650, Korea; 8 Chemical Kinomics Research Center, Korea Institute of Science and Technology, Seoul 130-650, Korea; khyoo@kist.re.kr; 9 Center for Biomaterials, Korea Institute of Science and Technology, Cheongryang, Seoul 130-650, Korea; 10 Department of Biomolecular Science, University of Science and Technology, Daejeon, Yuseong-gu 34113, Korea

**Keywords:** anti-inflammatory, inducible nitric oxide synthase (iNOS), nitric oxide, prostaglandine E_2_, triarylpyrazole

## Abstract

This article describes the design, synthesis, and in vitro anti-inflammatory screening of new triarylpyrazole derivatives. A total of 34 new compounds were synthesized containing a terminal arylsulfonamide moiety and a different linker between the sulfonamide and pyridine ring at position 4 of the pyrazole ring. All the target compounds were tested for both cytotoxicity and nitric oxide (NO) production inhibition in lipopolysaccharide (LPS)-induced RAW 264.7 macrophages. Compounds **1b**, **1d**, **1g**, **2a**, and **2c** showed the highest NO inhibition percentages and the lowest cytotoxic effect. The most potent derivatives were tested for their ability to inhibit prostaglandin E_2_ (PGE_2_) in LPS-induced RAW 264.7 macrophages. The IC_50_ for nitric oxide inhibition, PGE_2_ inhibition, and cell viability were determined. In addition, **1b**, **1d**, **1g**, **2a**, and **2c** were tested for their inhibitory effect on LPS-induced inducible nitric oxide synthase (iNOS) and Cyclooxygenase 2 (COX-2) protein expression as well as iNOS enzymatic activity.

## 1. Introduction

Inflammation is one of the most important and complicated defense mechanisms. Inflammation participates in vital pathological and physiological processes like infection and wound healing [1]. As a result of tissue damage, many chemical intermediates are released in the damaged area. The chemical intermediates (such as E, L, and P-selectin and chemokines) initiate activation and migration of white blood cells to the damaged area. Eosinophils and neutrophils are the first leucocytes that migrate to the affected area followed by macrophages that release a number of cytokines and growth factors that affect the surrounding tissues [2,3,4,5]. Inflammation can be acute, occurring as part of a healing process, or chronic inflammation, which arises from the over response of the immune system and can lead to tissue damage. Chronic inflammation contributes to several physiological disorders such as neurodegenerative diseases [6], cancer [7], inflammatory bowel disease [8], and arteriosclerosis [9].

At the inflammation site, monocytes are converted to macrophages that release a large amount of nitric oxide (NO). Nitric oxide is produced as a result of oxidation of l-arginine by one of the nitric oxide synthase family members: endothelial nitric oxide synthase (eNOS), neuronal NOS (nNOS), which is a calcium-dependent enzyme, and inducible NOS (iNOS), which is calcium-independent enzyme) [10,11]. The presence of pro-inflammatory and chemical stimuli, such as lipopolysaccharide (LPS), leads to over-expression of iNOS [12]. Successful NO production inhibitory agents act through inhibition of iNOS protein expression and/or inhibition of iNOS enzymatic activity.

In addition to nitric oxide, prostaglandins are another important inflammation phospholipid by-product [13]. Prostaglandin E_2_ (PGE_2_) plays an important role in most inflammation conditions [14], such as glomerulonephritis, which may lead to renal failure [15]. The production of PGE_2_ is initiated by membrane phospholipids that are converted to arachidonic acid under the effect of the phospholipases enzyme. Arachidonic acid is transformed first to prostaglandin H_2_, which finally produces PGE_2_ [16]. The increase in both prostaglandin E_2_ and nitric oxide in chronic inflammation cases can lead to severe complicated physiological disorders [17,18]. So, the inhibition of both PGE_2_ and nitric oxide could result in the discovery of new anti-inflammatory drug candidates.

**Figure 1:**
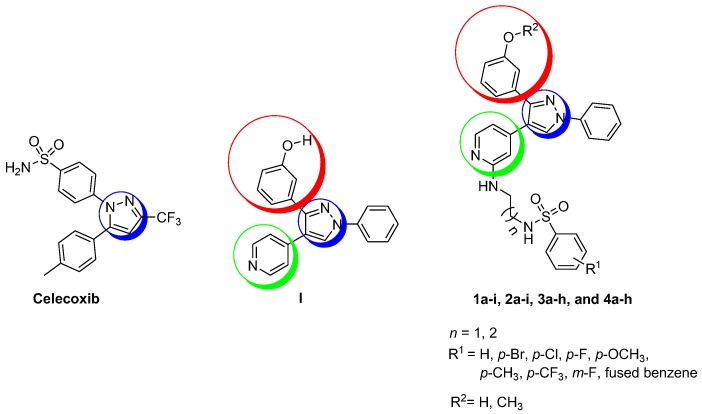
General structures of the target compounds, Celecoxib, and previously-reported pyrazole compound [40].

Several scaffolds have been investigated for their antiinflammatory activity, such as thiadiazole [19,20,21], chromones [22,23], triazoles [24], imidazole [25], and pyrazole. Many compounds with a pyrazole backbone have been proven to exhibit both anticancer [26,27,28,29,30,31] and antiinflammatory effects [32,33,34,35]. Celecoxib is an anti-inflammatory drug that contains diarylpyrazole as a back bone and works through inhibition of the COX-2 enzyme [36,37]. Previously, we reported the synthesis of a series of triarylpyrazoles [38,39,40,41], from which compound **I** (Figure 1) showed the highest activity for both nitric oxide and PGE_2_ production inhibition [40]. In the current work and based on our previous work, we synthesized a new series of triarylpyrazole derivatives. The new series contains 2-substituted pyridine at position 4 of the pyrazole ring. The substitutions contain a terminal sulfonamide moiety and a different linker between the sulfonamide and pyridine ring. The linker we used to investigate the effect of linker length on the activity was either ethylene or propylene. The new series was screened for its ability to inhibit nitric oxide; their cytotoxicity on RAW 264.7 macrophages was also investigated. The most potent compounds were tested for their inhibitory effect on PGE_2_ and iNOS expression. 

## 2. Results and Discussion

### 2.1. Chemistry

**Scheme 1:**
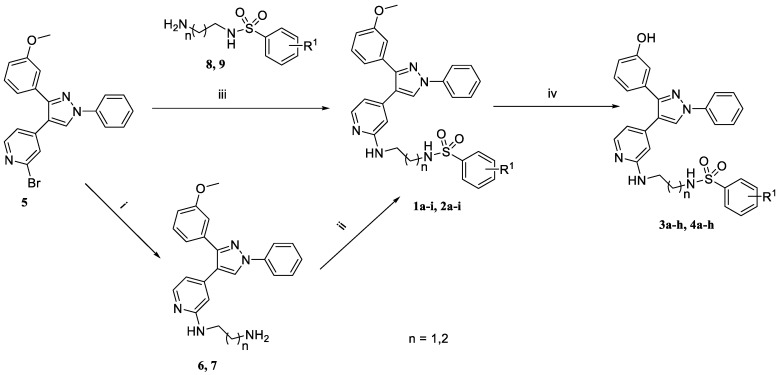
Synthesis of final target compounds **1a**–**i**, **2a**–**i**, **3a**–**h**, and **4a**–**h**. Reagents and conditions: (i) 1,2-ethylenediamine or 1,3-propylenediamine, reflux 8 h; (ii) appropriate aryl sulfonyl chloride, Triethylamine, Dichloromethane, 0 °C, overnight; (iii) pyridine, **8a**–**i** or **9a**–**i**, reflux 12 h; and (vii) BBr_3_, DCM, −78 °C; 0 °C, overnight.

The synthesis of the final target compounds **1a**–**i**, **2a**–**i**, **3a**–**h**, and **4a**–**h** was achieved by adopting the synthetic strategy illustrated in Scheme 1. We first synthesized the side chains **8a**–**i** and **9a**–**i**. The main intermediate **5** was synthesized according to previously reported procedures [42,43]. Eventually, the target compounds **1a**–**i** and **2a**–**i** were obtained by coupling compound **5** with **8a**–**i** and **9a**–**i** using pyridine as a solvent and refluxing for 12 h. Another pathway to obtain **1a**–**i** and **2a**–**i** was refluxing **5** with 1,2-ethylenediamine or 1,3-propylenediamine to produce **6** and **7**, which, upon reaction with the appropriate arylsulfonyl chloride in the presence of triethylamine, produced the desired final compounds **1a**–**i** and **2a**–**i**. Demethylation of compounds **1** and **2** using boron tribromide produced the hydroxyl final analogues **3a**–**h** and **4a**–**h** (Scheme 1). The structures of the final target compounds and their yields are represented in Table 1.

### 2.2. Biology

The ability of a certain molecule to inhibit the production of inflammatory mediator(s) is one of successful approach to treatment both chronic and acute inflammation. The final target compounds **1a**–**i**, **2a**–**i**, **3a**–**h**, and **4a**–**h** were tested for their ability to inhibit NO release in LPS-induced RAW 264.7 macrophages at three different concentrations (Table 2).

The tested derivatives exhibited diverse activity for NO production inhibition. All compounds inhibited NO release in a dose-dependent manner. For series **1a**–**i**, most of the compounds inhibited the production of NO by 50% or more at 10 µM. Compound **1b** (*p*-bromo) showed the highest inhibition at 68.66% followed by **1d** (*p*-flouro) with inhibition of 61.28%, then **1g** (*p*-CF_3_) with inhibition 60.80%. Compound **1a**, **1f**, and **1i** had moderate activity with 52.93%, 53.09%, and 51.51% inhibition, respectively. Regarding compounds **2a**–**i**, the highest inhibition was obtained from compounds **2a** (62.76%), **2c** (59.09%), **2g** (56.03%), **2d** (55.00%), and **2b** (52.44%). Generally for methoxy series, derivatives with ethylene bridges were more active compared to compounds with propylene bridges. In addition, compounds with electron-withdrawing groups were more potent compared to compounds with electron-donating groups, and *para* substitutions were slightly more active than *meta* substitutions. The electronic nature and the position of the substituents were other important factors that confer optimum affinity to the receptor site. 

Derivatives containing hydroxyl group, **3a**–**h** and **4a**–**h**, were less active compared to methoxy derivatives. The highest percent inhibition for **3a**–**h** was exhibited by 3a (48.97%). Series **4a**–**h** showed good inhibition with the highest demonstrated by compound **4b** (63.85%) followed by **4e** (59.69%), **4a** (59.25%), **4f** (53.82%), and **4g** (51.15%), as illustrated in Table 2. The methoxy group is more hydrophobic and bulkier than hydroxyl, and this might affect the activity by enhancing the molecule’s ability to cross the cell membrane and/or increasing the affinity with the target receptor site. Furthermore, the most active compounds (e.g., **1b**) were more active than the lead compound possessing no tether on the pyridyl ring [33]. So, this side chain is an important contributor to the inhibition activity, which could improve the molecular affinity to its receptor site.

In addition to NO inhibition, the cytotoxic activity of compounds **1a**–**i**, **2a**–**i**, **3a**–**h**, and **4a**–**h** in RAW 264.7 macrophages were measured using the 3-(4,5-dimethylthiazol-2-yl)-2,5-diphenyltetrazolium bromide (MTT) assay to check whether the effects on the production of NO was caused by nonspecific cytotoxicity. The IC_50_ values for both nitric oxide inhibition and cell viability are presented in Table 3. 

Compounds **1a**–**i** and **2a**–**i** had high IC_50_ values in the cell viability test and all compounds had an IC_50_ of more than 169 μM. The IC_50_ for nitric oxide production inhibition was less than 14 μM. The most potent compound among the methoxy derivatives was **1b** with an IC_50_ of 7.90 μM followed by **2a**, **1d**, and **2c** with IC_50_ values of 8.04, 8.2, and 8.68 μM, respectively. These most potent molecules showed extreme safety expressed by very high IC_50_ values as cytotoxic agents. This means that their inhibitory effect against NO production is not due to the cytotoxic effect. Compounds **3a**–**h** and **4a**–**h** showed cytotoxic effects at low doses and the IC_50_s for nitric oxide inhibition were close to the IC_50_s of the cell viability test. From Table 3, it can be predicted that the inhibitory effect of hydroxyl-containing compounds is due to the cytotoxic effect. 

Compounds **1b**, **1d**, **1g**, **2a**, and **2c**, which exhibited the highest activities regarding nitric oxide inhibition and the highest IC_50_ values in the cell viability test, were investigated for their ability to inhibit PGE_2_ production in LPS-induced RAW 264.7 macrophages at 1, 5, and 10 µM. The investigation results are shown in Table 4. Compounds **1b**, **1g**, **2a**, and **2c** were able to inhibit more than 50% of the prostaglandin production at a dose 5 μM. The five compounds were able to reduce PGE_2_ production by over 75% at a dose of 10 μM. Compound **1g** was the most potent compound with an IC_50_ of 4.55 μM followed by **2c**, **1b**, **2a**, and **1d** with IC_50_ values of 4.68, 4.72, 4.87, and 5.06 μM, respectively.

**Figure 2:**
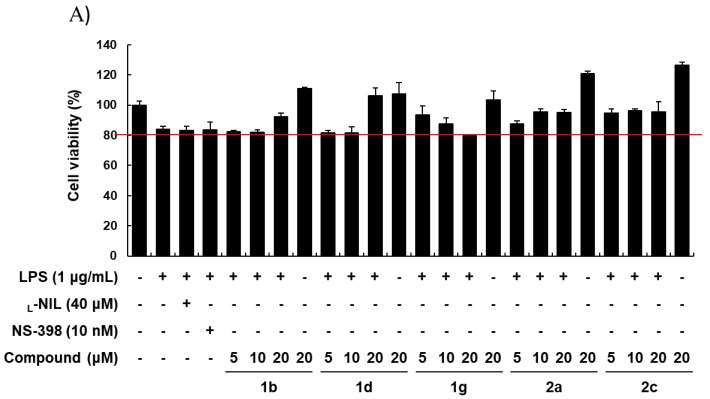

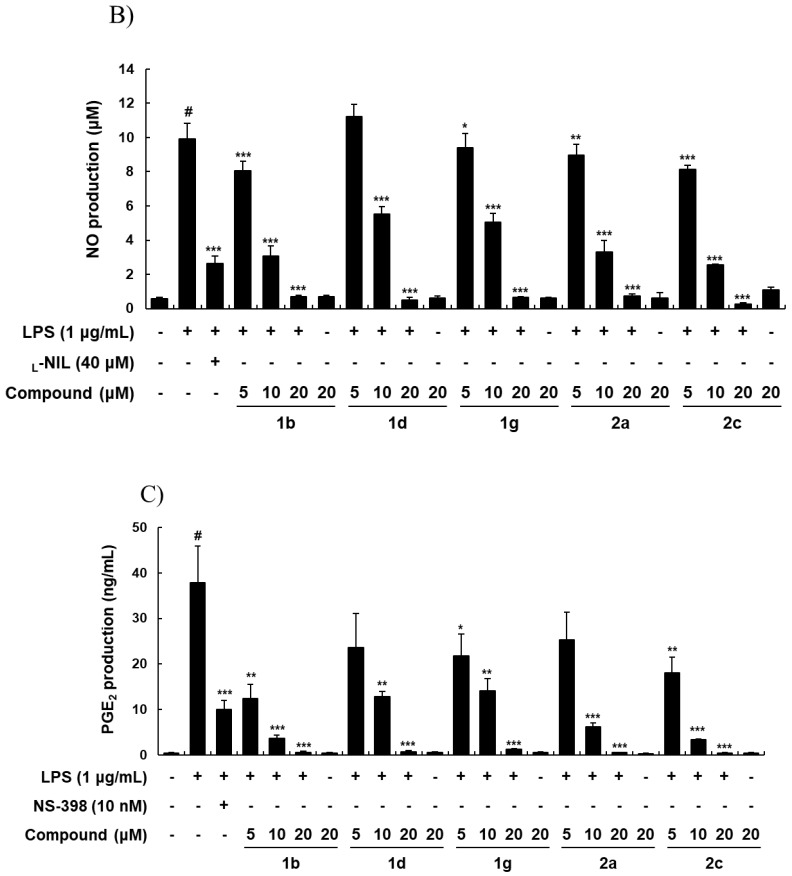
(**A**) In vitro cytotoxicity, (**B**) nitric oxide inhibition, and (**C**) prostaglandin E_2_ inhibition of compounds **1b**, **1d**, **1g**, **2a**, and **2c**. Data are presented as the means ± SD of three independent experiments. # *p* < 0.05 versus the control cells; *** *p* < 0.001 versus lipopolysaccharide-stimulated cells; ** *p* < 0.05 versus lipopolysaccharide-stimulated cells; * statistical significances were compared using ANOVA and Dunnett’s post hoc test.

The cumulative activities of compounds **1b**, **1d**, **1g**, **2a**, and **2c** are illustrated in Figure 2 using *N*-(2-cyclohexyloxy-4-nitrophenyl)methanesulfonamide (NS398) and N^6^-(1-Iminoethyl)-l-lysine (l-NIL) as standard compounds for PGE_2_ production inhibition and NO production inhibition, respectively. The tested compounds showed low cytotoxic activity in the viability test. A significant reduction in both nitric oxide and PGE_2_ production was observed starting from 5 μM. At 20 μM, the production of both inflammatory mediators was restored to normal levels.

**Figure 3:**
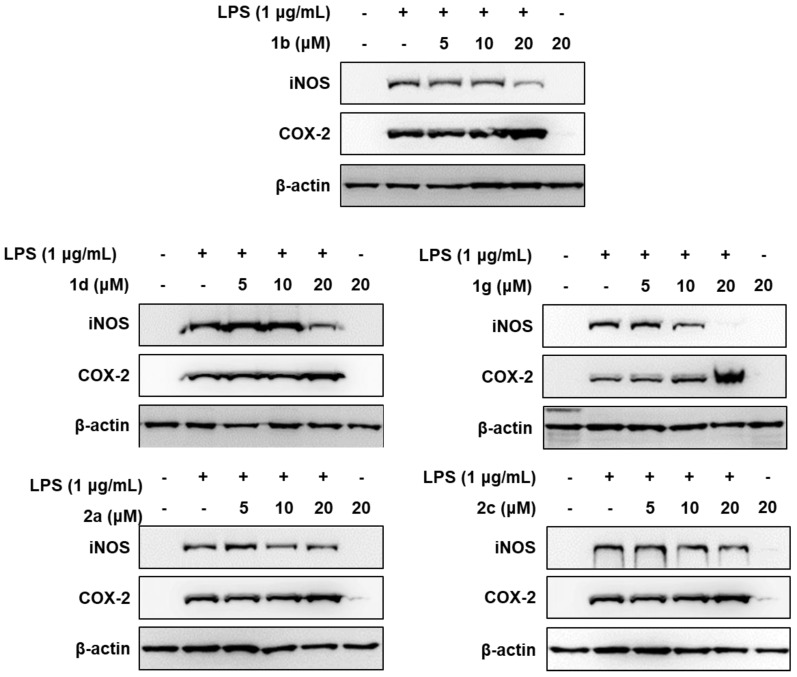
Inhibitory activity of compounds **1b**, **1d**, **1g**, **2a**, and **2c** on inducible nitric oxide synthase (iNOS) and Cyclooxygenase 2 (COX-2) Cellular lysates were prepared from the with/without pretreatment tested compound (5, 10, and 20 μM) for one hour and then with LPS (1 μg/mL) for 24 h. Total cellular proteins were resolved by sodium dodecyl sulphate-polyacrylamide gel electrophoresis (SDS-PAGE), transferred to Polyvinylidene fluoride (PVDF) membranes, and detected with specific iNOS and COX-2 antibodies. β-actin was used as an internal control.

**Figure 4:**
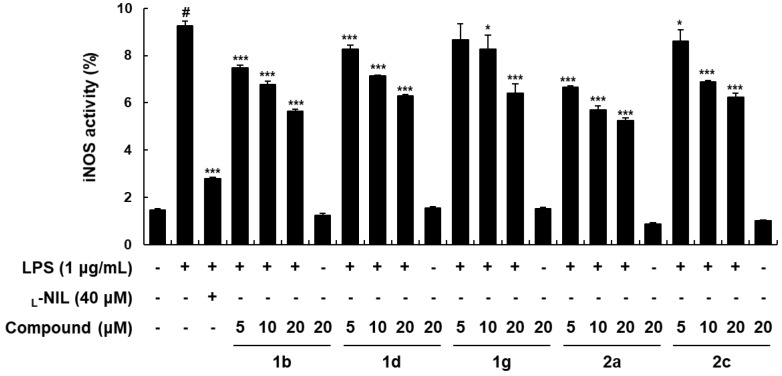
Effect of compounds **1b**, **1d**, **1g**, **2a**, and **2c** on iNOS activity. Following pretreatment with lipopolysaccharide (LPS, 1 µg/mL) for 12 h and wash with phosphate buffer solution (PBS), cells were treated with **1g** (5, 10, or 20 μM) for 12 h N^6^-(1-Iminoethyl)-l-lysine. (l-NIL) (40 μM) was used as the positive control in the assay. Levels of NO in culture media were quantified using the Griess reaction assay. Data are presented as the means ± SD of three independent experiments. # *p* < 0.05 versus the control cells; *** *p* < 0.001 versus LPS-stimulated cells; * statistical significances were compared using ANOVA and Dunnett’s post hoc test.

As a result of their activity against both NO and PGE_2_ production and low cellular toxicity, compounds **1b**, **1d**, **1g**, **2a**, and **2c** were tested for their inhibitory effect on the expression of both iNOS and COX-2. The cellular lysates were prepared from the with- and without-pretreatment tested compounds (5, 10, 20 μM) for one hour and then with LPS (1 μg/mL) for 24 h, using β-actin as a reference. The results are shown in Figure 3. Compound **1g**, possessing an ethylene spacer, 3-methoxyphenyl at position 3 of the pyrazole ring, and a *p*-(trifluoromethyl)phenyl terminal ring, showed complete inhibition of iNOS expression at 20 µM. Compounds **1b** and **1d** exhibited a partial inhibitory effect against iNOS at the same concentration (Figure 3 and Figure 4). Compound **1g** might express its inhibitory effect on NO production mainly through inhibition of iNOS protein expression and partially through inhibition of iNOS enzyme activity.

## 3. Materials and Methods

### 3.1. General

All chemicals were commercially available and used with no further purification. The final compounds and intermediates were purified by column chromatography using silica gel (0.040–0.063 mm, 230–400 mesh) and technical grade solvents. Analytical thin layer chromatography (TLC) was adopted on silica gel 60 F_254_ plates from Merck (Merck, Massachusetts, MA, USA). Purity percentages of the target compounds were confirmed to be more than 96% by liquid chromatography-mass spectrometry (LC-MS). Proton nuclear magnetic resonance (^1^H-NMR) and carbon NMR (^13^C-NMR) spectra were recorded on a Bruker Avance 400 or 300 spectrometer (Massachusetts, MA, USA) using tetramethylsilane as an internal standard and signals are described as s (singlet), d (doublet), t (triplet), q (quartet), p (pentet), m (multiplet), brs (broad singlet), or dd (doublet of doublets). LC-MS analysis was carried out using the following system: Waters 2998 photodiode array detector, Waters 3100 mass detector, Waters SFO system fluidics organizer, Waters 2545 binary gradient module, Waters reagent manager, Waters 2767 sample manager, Waters 2998 photodiode and Sunfire™ C18 column (4.6 × 50 mm, 5 μm particle size) (Waters, Massachusetts, MA, USA). The solvent gradient = 95% A at 0 min, 1% A at 5 min. Solvent A was 0.035% trifluoroacetic acid (TFA) in water, solvent B was 0.035% TFA in CH_3_OH, and the flow rate was 3.0 mL/min. The area under the curve (AUC) was calculated using Waters MassLynx 4.1 Waters, Massachusetts, MA, USA) software. Solvents and liquid reagents were transferred using hypodermic syringes. Melting points were obtained on a Walden Precision Apparatus Electro thermal 9300 apparatus (Stone, Staffordshire, England) and were uncorrected.

### 3.2. Synthesis of N-(2-aminoethyl)benzenesulfonamide *(**8a**)*, N-(2-aminoethyl)substituted benzenesulfonamides *(**8b***–***i**)*, N-(3-aminopropyl)benzenesulfonamide *(**9a**)*, and N-(3-aminopropyl)benzenesulfonamides *(**9b***–***i**)*.

These compounds were synthesized performing the six-step procedure reported in the literature [42].

### 3.3. Synthesis of 2-bromo-4-(3-(3-methoxyphenyl)-1-phenyl-1H-pyrazol-4-yl)pyridine *(**5**)*

This compound was synthesized performing the four-step procedure reported in the literature [40].

### 3.4. Synthesis of N^1^-(4-(3-(3-Methoxyphenyl)-1-phenyl-1H-pyrazol-4-yl)pyridin-2-yl) ethane1,2-diamine *(**6**)* and N^1^-(4-(3-(3-Methoxyphenyl)-1-phenyl-1H-pyrazol-4-yl)pyridin-2-yl) propane-1,3-diamine *(**7**)*

These compounds were synthesized utilizing the five-step procedure reported in the literature [43]. The detailed procedures are mentioned in the supplementary file.

### 3.5. General Procedure for Synthesis of the Target Compounds N-(2-((4-(3-(3-methoxyphenyl)-1-phenyl-1H-pyrazol-4-yl)pyridin-2-yl)amino)ethyl)arylsulfonamides *(**1a***–***i**)* and N-(3-((4-(3-(3-methoxyphenyl)-1-phenyl-1H-pyrazol-4-yl)pyridin-2-yl)amino)propyl)arylsulfonamides *(**2a***–***i**)*.

#### 3.5.1. Method A

To a solution of compound **6** or **7** (0.2 mmol) in anhydrous dichloromethane (5 mL), triethylamine (50.5 mg, 0.5 mmol) was added at 0 °C. A solution appropriate arylsulfonyl chloride (0.21 mmol) in anhydrous dichloromethane (1 mL) was added dropwise. The reaction mixture was stirred at room temperature for 24 h. When the reaction was finished, the solvent was removed in vacuo, and the residue was partitioned between ethyl acetate (5 mL) and water (5 mL). The organic layer was separated and the aqueous layer was extracted with ethyl acetate (3 × 10 mL). The combined organic layer was washed with saturated saline (2 × 5 mL) and the organic solvent was evaporated under reduced pressure. The residue was purified by column chromatography (silica gel, hexane-ethyl acetate 4:1 *v*/*v*) to produce the required product.

#### 3.5.2. Method B

A mixture of compound **5** (81 mg, 0.2 mmol) and compound **8** or **9** (0.2 mmol) in pyridine was heated at 100 °C for 24 h. After complete reaction, monitored by thin-layer chromatograph (TLC), pyridine was removed under reduced pressure. The residue was partitioned between water (50 mL) and ethylacetate (50 mL). The organic layer was separated and washed three times with distilled water (3 × 50 mL) and dried over Na_2_SO_4_. The organic layer was evaporated and the residue was purified using column chromatography.

*N-(2-((4-(3-(3-Methoxyphenyl)-1-phenyl-1H-pyrazol-4-yl)pyridin-2-yl)amino)ethyl)benzenesulfonamide* (**1a**); White solid (65%); mp 104–106 °C; ^1^H-NMR (300 MHz, CDCl_3_) δ 7.93 (s, 1H, Ar-H), 7.86–7.79 (m, 4H, Ar-H), 7.51–7.40 (m, 3H, Ar-H), 7.25–7.21 (m, 5H, Ar-H), 6.92–6.88 (m, 1H, Ar-H), 6.77 (d, *J* = 9.0 Hz, 1H, Ar-H), 6.70 (s, 1H, Ar-H), 6.42 (d, *J* = 6.0 Hz, 1H, Ar-H), 6.24 (s, 1H, Ar-H), 4.98 (brs, 1H, NH), 3.65 (s, 3H, OCH_3_), 3.32 (d, *J* = 3.0 Hz, 2H, CH_2_), 3.09 (d, *J* = 6.0 Hz, 2H, CH_2_); ^13^C-NMR (75 MHz, CDCl_3_) δ 159.6, 158.6, 147.4, 141.9, 140.1, 140.1, 139.5, 139.4, 132.4, 131.0, 130.0, 129.9, 128.9, 128.8, 127.6, 126.9, 125.1, 122.7, 120.0, 115.8, 114.7, 112.4, 105.9 (Ar-C), 55.3 (OCH_3_), 43.9 (CH_2_), 41.6 (CH_2_); LC-MS (*m*/*z*) calculated for C_29_H_27_N_5_O_3_S: 525.18, found: 526.0 (M + 1)^+^.

*4-Bromo-N-(2-((4-(3-(3-methoxyphenyl)-1-phenyl-1H-pyrazol-4-yl)pyridin-2-yl)amino)ethyl)benzenesulfonamide* (**1b**); White solid (61%); mp 136–138 °C; ^1^H-NMR (300 MHz, CDCl_3_) δ 7.96 (s, 1H, Ar-H), 7.89 (d, *J* = 6.0 Hz, 1H, Ar-H), 7.64 (d, *J* = 9 Hz, 2H, Ar-H), 7.58–7.55 (m, 2H, Ar-H), 7.31–7.28 (m, 6H, Ar-H), 6.93 (d, *J* = 6.0 Hz, 1H, Ar-H), 6.81 (d, *J* = 9.0 Hz, 1H, Ar-H), 6.72 (s, 1H, Ar-H), 6.48 (d, *J* = 6.0 Hz, 1H, Ar-H), 6.24 (s, 1H, Ar-H), 4.81 (brs, 1H, NH), 3.69 (s, 3H, OCH_3_), 3.36 (brs, 2H, CH_2_), 3.12 (brs, 2H, CH_2_); ^13^C-NMR (75 MHz, CDCl_3_) δ 159.7, 158.5, 147.2, 142.2, 140.2, 139.5, 139.3, 139.2, 132.6, 132.6, 132.1, 131.0, 130.0, 128.8, 128.5, 127.6, 127.1, 125.1, 122.7, 119.8, 115.8, 114.8, 112.6, 106.1 (Ar-C), 55.3 (OCH_3_), 44.4 (NH-CH_2_), 41.6 (CH_2_-NH); LC-MS (*m*/*z*) calculated for C_29_H_26_BrN_5_O_3_S: 603.09, found: 605.0 (M + 2)^+^.

*4-Chloro-N-(2-((4-(3-(3-methoxyphenyl)-1-phenyl-1H-pyrazol-4-yl)pyridin-2-yl)amino)ethyl)benzenesulfonamide* (**1c**); White solid (60%); mp 132–134 °C; ^1^H-NMR (300 MHz, CDCl_3_) δ 7.94 (s, 1H, Ar-H), 7.85 (d, *J* = 6.0 Hz, 1H, Ar-H), 7.72–7.69 (m, 2H, Ar-H), 7.54-7.51 (m, 2H, Ar-H), 7.39–7.22 (m, 6H, Ar-H), 6.91 (d, *J* = 9.0 Hz, 1H, Ar-H), 6.78 (d, *J* = 9.0 Hz, 1H, Ar-H), 6.70 (s, 1H, Ar-H), 6.43 (d, *J* = 3.0 Hz, 1H, Ar-H), 6.24 (s, 1H, Ar-H), 4.95 (brs, 1H, NH), 3.66 (s, 3H, OCH_3_), 3.33 (d, *J* = 3.0 Hz, 2H, NH-CH_2_), 3.09 (d, *J* = 3.0 Hz, 2H, -CH_2_-NH); ^13^C-NMR (75 MHz, CDCl_3_) δ 159.6, 158.5, 147.3, 142.0, 140.1, 139.5, 139.3, 138.6, 131.0, 129.9, 129.1, 128.8, 127.6, 125.1, 122.7, 119.9, 115.8, 114.7, 112.5, 106.9 (Ar-C), 55.2 (OCH_3_), 44.1 (NH-CH_2_), 41.5 (-CH_2_-NH).; LC-MS (*m*/*z*) calculated for C_29_H_26_ClN_5_O_3_S: 559.14, found: 560.0 (M + 1)^+^.

*4-Fluoro-N-(2-((4-(3-(3-methoxyphenyl)-1-phenyl-1H-pyrazol-4-yl)pyridin-2-yl)amino)ethyl)benzenesulfonamide* (**1d**); White solid (67%); mp 155–156 °C; ^1^H-NMR (400 MHz, CDCl_3_) δ 7.83 (s, 1H, Ar-H), 7.71–7.67 (m, 3H, Ar-H), 7.19–7.15 (m, 5H, Ar-H), 6.99 (t, *J* = 8.4 Hz, 2H, Ar-H), 6.80 (d, *J* = 8.4 Hz, 1H, Ar-H), 6.67 (d, *J* = 8.8 Hz, 1H, ar-H), 6.59 (s, 1H, Ar-H), 6.34 (d, *J* = 5.2 Hz, 1H, Ar-H), 6.13 (s, 1H, Ar-H), 4.85 (brs, NH), 3.56 (s, 3H, OCH_3_), 3.23 (s, 2H, NH-CH_2_), 2.98 (d, *J* = 4.4 Hz, 2H, -CH_2_-NH); ^13^C-NMR (100 MHz, CDCl_3_) δ 159.7, 158.6, 147.1, 142.2, 140.1, 139.5, 139.3, 136.1, 130.9, 130.0, 129.7, 129.6, 129.5, 128.9, 128.7, 125.2, 125.0, 122.7, 119.8, 116.0, 115.0, 112.5, 105.9 (Ar-C), 55.3 (OCH_3_), 44.2 (CH_2_), 41.6 (CH_2_); LC-MS (*m*/*z*) calculated for C_29_H_26_FN_5_O_3_S: 543.17, found: 544.0 (M + 1)^+^.

*4-Methoxy-N-(2-((4-(3-(3-methoxyphenyl)-1-phenyl-1H-pyrazol-4-yl)pyridin-2-yl)amino)ethyl)benzenesulfonamide* (**1e**); White solid (62%); mp 144–146 °C; ^1^H-NMR (400 MHz, CDCl_3_) δ 7.94 (s, 1H, Ar-H), 7.88 (d, *J* = 4.0 Hz, 1H, Ar-H), 7.74 (d, *J* = 8.0 Hz, 2H, Ar-H), 7.33–7.23 (m, 6H, Ar-H), 6.91 (d, *J* = 8.0 Hz, 3H, Ar-H), 6.79–6.77 (m, 1H, Ar-H), 6.70–6.69 (m, 1H, Ar-H), 6.43 (dd, *J* = 8.0, *J* =4.0 Hz, 1H, Ar-H), 6.23 (s, 1H, Ar-H), 4.85 (s, NH), 3.83 (s, 3H, OCH_3_), 3.67 (s, 3H, OCH_3_), 3.34 (t, *J* = 8.0 Hz, 2H, NH-CH_2_-), 3.08 (t, *J* = 8.0 Hz, 2H, -CH_2_-NH); ^13^C-NMR (100 MHz, CDCl_3_) δ 162.6, 159.6, 158.6, 147.5, 141.9, 140.1, 139.5, 139.4, 131.6, 131.0, 129.9, 129.1, 128.8, 127.6, 125.1, 122.7, 120.0, 115.7, 114.7, 114.1, 112.4, 105.9 (Ar-C), 55.5 (OCH_3_), 55.3 (OCH_3_), 43.8 (CH_2_), 41.5 (CH_2_); LC-MS (*m*/*z*) calculated for C_30_H_29_N5O_4_S: 555.19, found: 556.0 (M + 1)^+^.

*N-(2-((4-(3-(3-Methoxyphenyl)-1-phenyl-1H-pyrazol-4-yl)pyridin-2-yl)amino)ethyl)-4-methyl benzenesulfonamide* (**1f**); White solid (69%); mp 150–152 °C; ^1^H-NMR (300 MHz, CDCl_3_) δ 7.97 (s, 1H, Ar-H), 7.91 (d, *J* = 6.0 Hz, 1H), 7.71 (d, *J* = 9.0 Hz, 2H, Ar-H), 7.32–7.26 (m, 7H), 6.94 (d, *J* = 9.0 Hz, 1H, Ar-H), 6.81 (d, *J* = 6.0 Hz, 1H, Ar-H), 6.72 (s, 1H, Ar-H), 6.49 (d, *J* = 3.0 Hz, 1H, Ar-H), 6.26 (s, 1H, Ar-H), 4.97 (brs, 1H, NH), 3.70 (s, 3H, OCH_3_), 3.36 (brs, 2H, NH-CH_2_-), 3.12 (t, *J* = 6.0 Hz, 2H, -CH_2_-NH), 2.43 (s, 3H, CH_3_); ^13^C-NMR (75 MHz, CDCl_3_) δ 159.7, 158.5, 147.4, 143.1, 142.1, 140.1, 139.6, 139.3, 137.1, 131.1, 129.9, 129.5, 128.8, 127.6, 127.0, 125.1, 122.7, 120.0, 115.7, 114.7, 112., 105.5 (Ar-C), 55.3 (OCH3), 44.0 (CH_2_), 41.7 (CH_2_), 21.4 (CH_3_); LC-MS (*m*/*z*) calculated for C_30_H_29_N_5_O_3_S: 539.20, found: 540.0 (M + 1)^+^.

*N-(2-((4-(3-(3-Methoxyphenyl)-1-phenyl-1H-pyrazol-4-yl)pyridin-2-yl)amino)ethyl)-4-(trifluoromethyl)benzenesulfonamide* (**1g**); White solid (74%); mp 132–134 °C; ^1^H-NMR (300 MHz, CDCl_3_) δ 7.91 (d, *J* = 9.0 Hz, 4H, Ar-H), 7.71 (s, 1H, Ar-H), 7.30 (brs, 6H, Ar-H), 6.93 (s, 1H, Ar-H), 6.80 (s, 1H, Ar-H), 6.71 (s, 2H, Ar-H), 6.48 (s, 1H, Ar-H), 6.25 (s, 1H, Ar-H), 4.87 (brs, 1H, NH), 3.67 (s, 3H, OCH_3_), 3.37 (s, 2H, NH-CH_2_-), 3.14 (s, 2H, -CH_2_-NHSO_2_); ^13^C-NMR (75 MHz, CDCl_3_) δ 159.7, 158.5, 147.1, 147.0, 143.8, 142.4, 139.5, 139.4, 139.2, 134.1, 133.7, 130.9, 130.1, 129.9, 128.9, 127.4, 126.0, 125.2, 125.0, 122.7, 119.7, 115.8, 114.7, 112.8, 106.2 (Ar-C), 55.3 (OCH_3_), 44.7 (CH_2_), 41.7 (CH_2_); LC-MS (*m*/*z*) calculated for C_30_H_26_F_3_N_5_O_3_S: 593.17, found: 594.0 (M + 1)^+^.

*3-Fluoro-N-(2-((4-(3-(3-methoxyphenyl)-1-phenyl-1H-pyrazol-4-yl)pyridin-2-yl)amino)ethyl)benzenesulfonamide* (**1h**); White solid (66%); mp 118–120 °C; ^1^H-NMR (300 MHz, CDCl_3_) δ 7.94 (s, 1H, Ar-H), 7.85 (d, *J* = 6.0 Hz, 1H, Ar-H), 7.59 (s, 1H, Ar-H), 7.49 (s, 1H, Ar-H), 7.46–7.37 (m, 2H, Ar-H), 7.29–7.21 (m, 6 H), 6.90 (d, *J* = 9.0 Hz, 1H, Ar-H), 6.78 (d, *J* = 9.0 Hz, 1H, Ar-H), 6.71 (s, 1H), 6.43 (d, *J* = 6.0 Hz, 1H, Ar-H), 6.25 (s, 1H, Ar-H), 4.99 (brs, 1H, NH), 3.65 (s, 3H, OCH_3_), 3.33 (brs, 2H, NH-CH_2_-), 3.10 (t, *J* = 6.0 Hz, 2H, -CH_2_-NH-SO_2_); ^13^C-NMR (75 MHz, CDCl_3_) δ 159.6, 158.6, 147.3, 142.2, 142.0, 140.2, 139.5, 139.4, 130.9, 130.8, 130.7, 129.9, 128.8, 127.6, 125.1, 122.7, 119.9, 119.6, 119.3, 115.7, 114.7, 114.4, 114.1, 112.5, 106.0 (Ar-C), 55.2 (OCH_3_), 44.2 (CH_2_), 41.5( CH_2_); LC-MS (*m*/*z*) calculated for C_29_H_26_FN_5_O_3_S: 543.17, found: 544.0 (M + 1)^+^.

*N-(2-((4-(3-(3-Methoxyphenyl)-1-phenyl-1H-pyrazol-4-yl)pyridin-2-yl)amino)ethyl)naphthalene-1-sulfonamide* (**1i**); White solid (71%); mp 178–180 °C; ^1^H-NMR (400 MHz, CDCl_3_) δ 8.38 (s, 1H, Ar-H), 7.91–7.85 (m, 4H, Ar-H), 7.75 (dd, *J* = 8.8 Hz, *J* = 1.6 Hz, 1H, Ar-H), 7.64–7.55 (m, 2H, Ar-H), 7.34–7.21 (m, 6H, Ar-H), 7.02 (brs, 1H, NH), 6.89 (dd, *J* = 8.4 Hz, *J* = 2.0 Hz, 1H, Ar-H), 6.76 (d, *J* = 7.6 Hz, 1H, Ar-H), 6.68 (s, 1H, Ar-H), 6.42 (d, *J* = 4.8 Hz, 1H, Ar-H), 6.17 (s, 1H, Ar-H), 4.87 (brs, 1H, NH), 3.65 (s, 3H, OCH_3_), 3.34 (d, *J* = 4.4 Hz, 2H, NH-CH_2_-), 3.14 (t, *J* = 5.2 Hz, 2H, -CH_2_-NH-SO_2_ ); ^13^C-NMR (100 MHz, CDCl_3_) δ 159.6, 158.4, 147.1, 142.1, 140.1, 139.5, 139.4, 136.8, 134.6, 132.1, 130.9, 129.8, 129.3, 129.1, 128.8, 128.6, 128.2, 127.8, 127.6, 127.4, 125.1, 122.7, 122.3, 119.9, 115.7, 114.7, 112.4, 105.9 (Ar-C), 55.2 (OCH_3_), 44.3 (CH_2_), 41.5 (CH_2_); LC-MS (*m*/*z*) calculated for C_33_H_29_N_5_O_3_S: 575.20, found: 576.0 (M + 1)^+^.

*N-(3-((4-(3-(3-Methoxyphenyl)-1-phenyl-1H-pyrazol-4-yl)pyridin-2-yl)amino)propyl)benzenesulfonamide* (**2a**); White solid (60%); mp 119–120 °C; ^1^H-NMR (300 MHz, CDCl_3_) δ 7.98 (d, *J* = 12.0 Hz, 2H, Ar-H), 7.83 (d, *J* = 9.0 Hz, 2H, Ar-H), 7.52–7.45 (m, 3H, Ar-H), 7.31–7.24 (m, 6H, Ar-H), 6.93 (d, *J* = 6.0 Hz, 1H, Ar-H), 6.79 (d, *J* = 9.0 Hz, 1H, Ar-H), 6.71 (s, 1H, Ar-H), 6.45 (d, *J* = 3.0 Hz, 1H, Ar-H), 6.22 (s, 1H, Ar-H), 4.73 (brs, 1H, NH), 3.68 (s, 3H, OCH_3_), 3.31 (brs, 2H, NH-CH_2_-), 2.97 (brs, 2H, -CH_2_-NH-SO_2_), 1.67–1.58 (m, 2H, -CH_2_-); ^13^C-NMR (75 MHz, CDCl_3_) δ 159.6, 158.7, 147.2, 142.1, 140.4, 140.1, 139.5, 139.3, 132.2, 131.0, 129.9, 128.9, 128.8, 127.6, 126.9, 125.1, 122.7, 120.0, 115.7, 114.8, 112.1, 105.7 (Ar-C), 55.3 (OCH_3_), 40.1 (CH_2_), 38.3 (CH_2_), 29.9 (CH_2_); LC-MS (*m*/*z*) calculated for C_30_H_29_N_5_O_3_S: 539.20, found: 540.0 (M + 1)^+^.

*4-Bromo-N-(3-((4-(3-(3-methoxyphenyl)-1-phenyl-1H-pyrazol-4-yl)pyridin-2-yl)amino)propyl)benzenesulfonamide* (**2b**); White solid (62%); mp 117–119 °C; ^1^H-NMR (400 MHz, CDCl_3_) δ 7.98 (s, 1H, Ar-H), 7.90 (d, *J* = 5.6 Hz, 1H, Ar-H), 7.69 (d, *J* = 8.8 Hz, 2H, Ar-H), 7.60 (d, *J* = 8.8 Hz, 2H, Ar-H), 7.35–7.20 (m, 6H, Ar-H), 6.95 (dd, *J* =8.0, *J* = 2.0 Hz, 1H, Ar-H), 6.72 (t, *J* = 2.0 Hz, 1H, Ar-H), 6.49 (dd, *J* = 6.0 Hz, *J* = 1.6 Hz, 1H, Ar-H), 6.28 (s, 1H, Ar-H), 5.21 (brs, 1H, NH), 3.70 (s, 3H, OCH_3_), 3.36 (q, *J* = 6.4 Hz, 2H, NH-CH_2_-), 2.99 (d, *J* = 5.6 Hz, 2H, -CH_2_NHSO_2_), 1.68 (p, *J* = 6.4 Hz, 2H, CH_2_-CH_2_-CH_2_); ^13^C-NMR (75 MHz, CDCl_3_) δ 159.7, 158.9, 147.3, 142.1, 140.2, 139.6, 139.4, 132.2, 131.0, 130.0, 128.8, 128.5, 127.6, 127.1, 125.1, 122.8, 120.0, 115.8, 114.8, 112.1, 105.9 (Ar-C), 55.3 (OCH_3_), 40.2 (CH_2_), 38.3(CH_2_), 29.9(CH_2_); LC-MS (*m*/*z*) calculated for C_30_H_28_BrN_5_O_3_S: 617.11, found: 618.00 (M + 1)^+^.

*4-Chloro-N-(3-((4-(3-(3-methoxyphenyl)-1-phenyl-1H-pyrazol-4-yl)pyridin-2-yl)amino)propyl)benzenesulfonamide* (**2c**); White solid (62%); mp 98–100 °C; ^1^H-NMR (400 MHz, CDCl_3_) δ 7.95 (s, 1H, Ar-H), 7.93 (d, *J* = 5.2 Hz, 1H, Ar-H), 7.75 (d, *J* = 8.4 Hz, 2H, Ar-H), 7.42 (d, *J* = 8.4 Hz, 2H, Ar-H), 7.32–7.25 (m, 6H, Ar-H), 6.93 (dd, *J* = 8.0 Hz, *J* = 2.0 Hz, 1H, Ar-H), 6.79 (d, *J* = 7.6 Hz, 1H, Ar-H), 6.71 (s, 1H, Ar-H), 6.45 (d, *J* = 5.6 Hz, 1H, Ar-H), 6.25 (s, 1H, Ar-H), 4.84 (brs, 1H, NH), 3.68 (s, 3H, OCH_3_), 3.34 (d, *J* = 5.6 Hz, 2H, NH-CH_2_-), 2.90 (brs, 2H, -CH_2_NHSO_2_), 1.66 (t, *J* = 6.0 Hz, 2H, -CH_2_-); ^13^C-NMR (100 MHz, CDCl_3_) δ 159.4, 158.4, 146.8, 142.8, 140.2, 139.3, 139.3, 138.9, 138.6, 130.9, 130.0, 129.5, 129.2, 128.8, 128.4, 127.6, 125.1, 122.7, 119.8, 115.7, 114.8, 112.0, 105.9 (Ar-C), 55.2 (OCH_3_), 40.0 (CH_2_), 38.2 (CH_2_), 29.9 (CH_2_); LC-MS (*m*/*z*) calculated for C_30_H_28_ClN_5_O_3_S: 573.16, found: 574.0 (M + 1)^+^.

*4-Fluoro-N-(3-((4-(3-(3-methoxyphenyl)-1-phenyl-1H-pyrazol-4-yl)pyridin-2-yl)amino)propyl)benzenesulfonamide* (**2d**); White solid (75%); mp 92–94 °C; ^1^H-NMR (300 MHz, CDCl_3_) δ 7.96 (s, 2H, Ar-H), 7.82 (d, *J* = 3.0 Hz, 2H, Ar-H), 7.32–7.28 (m, 6H, Ar-H), 7.14 (t, *J* = 9.0 Hz, 2H, Ar-H), 6.94 (d, *J* = 6.0 Hz, 1H, Ar-H), 6.80 (d, *J* = 6.0 Hz, 1H, Ar-H), 6.72 (s, 1H, Ar-H), 6.46 (d, *J* = 6.0 Hz, 1H, Ar-H), 6.24 (s, 1H, Ar-H), 4.60 (brs, 1H, NH), 3.69 (s, 3H, OCH_3_), 3.36 (s, 2H, NH-CH_2_-), 2.98 (brs, 2H, -CH_2_NHSO_2_), 1.66 (d, *J* = 6.0 Hz, 2H, -CH_2_-); ^13^C-NMR (75 MHz, CDCl_3_) δ 159.6, 158.7, 147.2, 140.1, 139.3, 131.0, 129.9, 129.6, 129.5, 128.8, 127.6, 125.1, 122.7, 119.9, 116.2, 115.9, 115.7, 114.8, 112.2, 106.0 (Ar-C), 55.2 (OCH_3_), 40.0 (CH_2_), 38.2 (CH_2_), 30.0 (CH_2_); LC-MS (*m*/*z*) calculated for C_30_H_28_FN_5_O_3_S: 557.19, found: 558.0 (M + 1)^+^.

*4-Methoxy-N-(3-((4-(3-(3-methoxyphenyl)-1-phenyl-1H-pyrazol-4-yl)pyridin-2-yl)amino)propyl)benzenesulfonamide* (**2e**); White solid (71%); mp 124–126 °C; ^1^H-NMR (300 MHz, CDCl_3_) δ 7.96 (s, 1H, Ar-H), 7.94 (d, *J* = 6.0 Hz, 1H, Ar-H), 7.76 (d, *J* = 9.0 Hz, 2H, Ar-H),7.33–7.24 (m, 6H, Ar-H), 6.93 (d, *J* = 9.0 Hz, 3H, Ar-H), 6.79 (d, *J* = 9.0 Hz, 1H, Ar-H), 6.71 (s, 1H, Ar-H), 6.54 (brs, 1H, NH), 6.45 (d, *J* = 3.0 Hz, 1H, Ar-H), 6.22 (s, 1H, Ar-H), 4.61 (brs, 1H, NH), 3.86 (s, 3H, OCH_3_), 3.68 (s, 3H, OCH_3_), 3.32 (t, *J* = 6.0 Hz, 2H, NH-CH_2_-), 2.96 (t, *J* = 6.0 Hz, 2H, -CH_2_NHSO_2_), 1.65 (p, *J* = 6.0 Hz, 2H, -CH_2_-); ^13^C-NMR (75 MHz, CDCl_3_) δ 159.7, 158.8, 147.5, 142.0, 140.1, 139.6, 139.4, 132.0, 131.1, 130.0, 128.8, 127.6, 125.1, 122.8, 120.1, 115.7, 114.8, 114.1, 112.2, 105.7 (Ar-C), 55.6 (OCH_3_), 55.3 (OCH_3_), 40.2 (CH_2_), 38.4(CH_2_), 30.0 (CH_2_); LC-MS (*m*/*z*) calculated for C_31_H_31_N_5_O_3_S: 569.21, found: 570.0 (M + 1)^+^.

*N-(3-((4-(3-(3-Methoxyphenyl)-1-phenyl-1H-pyrazol-4-yl)pyridin-2-yl)amino)propyl)-4-methylbenzenesulfonamide* (**2f**); Buff solid (72%); mp 94–96 °C; ^1^H-NMR (300 MHz, CDCl_3_) δ 7.95 (s, 1H, Ar-H), 7.91 (d, *J* = 6.0 Hz, 1H, Ar-H), 7.71 (d, *J* = 9.0 Hz, 2H, Ar-H),7.30–7.24 (m, 7H, Ar-H), 6.92 (d, *J* = 6.0 Hz, 1H, Ar-H), 6.79 (d, *J* = 6.0 Hz, 1H, Ar-H), 6.71 (s, 1H, Ar-H), 6.44 (d, *J* = 6.0 Hz, 1H, Ar-H), 6.23 (s, 1H, Ar-H), 4.78 (brs, 1H, NH), 3.67 (s, 3H, OCH_3_), 3.26 (brs, 2H, NH-CH_2_-), 2.94 (d, *J* = 3.0 Hz, 2H, -CH_2_NHSO_2_), 2.60 (s, 3H, CH_3_), 1.62 (d, *J* = 6.0 Hz, 2H, -CH_2_-); ^13^C-NMR (75 MHz, CDCl_3_) δ 160.2, 159.3, 147.9, 143.5, 142.5, 140.6, 140.1, 139.9, 137.8, 131.5, 130.4, 130.1, 129.3, 128.1, 127.5, 125.6, 123.2, 120.6, 116.2, 115.3, 112.5, 106.1 (Ar-C), 55.8 (OCH_3_), 40.7 (CH_2_), 38.9 (CH_2_), 30.2 (CH_2_), 22.0 (CH_3_); LC-MS (*m*/*z*) calculated for C_31_H_31_N_5_O_4_S: 553.21, found: 554.0 (M + 1)^+^.

*N-(3-((4-(3-(3-Methoxyphenyl)-1-phenyl-1H-pyrazol-4-yl)pyridin-2-yl)amino)propyl)-4-(trifluoromethyl)benzenesulfonamide* (**2g**); White solid (71%); mp 150–152 °C; ^1^H-NMR (300 MHz, CDCl_3_) δ 7.84–7.81 (m, 4H, Ar-H), 7.61 (d, *J* = 6.0 Hz, 2H, Ar-H), 7.49 (brs, 1H, NH), 7.21–7.16 (m, 6H, Ar-H), 6.82 (d, *J* = 6.0 Hz, 1H, Ar-H), 6.68 (d, *J* = 5.4, 1H, Ar-H), 6.60 (s, 1H, Ar-H), 6.33 (d, *J* = 3.9 Hz, Ar-H), 6.14 (s, 1H, Ar-H), 4.52 (brs, 1H, NH), 3.56 (s, 3H, OCH_3_), 3.26 (brs, 2H, NH-CH_2_-), 2.89 (brs, 2H, CH_2_NHSO_2_), 1.95 (brs, 2H, -CH_2_-); ^13^C-NMR (75 MHz, CDCl_3_) δ 159.7, 158.8, 147.1, 142.2, 140.1, 139.5, 139.3, 131.0, 129.9, 128.8, 127.6, 127.4, 126.1, 126.0, 125.1, 122.7, 119.9, 115.7, 114.8, 112.2, 106.1 (Ar-C), 55.2 (OCH_3_), 40.1 (CH_2_), 38.3 (CH_2_), 30.1 (CH_2_); LC-MS (*m*/*z*) calculated for C_31_H_28_ F_3_N_5_O_3_S: 607.19, found: 608.0 (M + 1)^+^.

*3-Fluoro-N-(3-((4-(3-(3-methoxyphenyl)-1-phenyl-1H-pyrazol-4-yl)pyridin-2-yl)amino)propyl)benzenesulfonamide* (**2h**); White solid (76%); mp 120–122 °C; ^1^H-NMR (300 MHz, CDCl_3_) δ 7.94 (s, 1H, Ar-H), 7.91 (d, *J* = 6.0 Hz, 1H, Ar-H), 7.30-7.22 (m, 10H, Ar-H), 6.93 (dd, *J* = 2.0 Hz, *J* = 6.0 Hz, 1H, Ar-H), 6.89 (d, *J* = 6.0 Hz, 1H, Ar-H), 6.70 (s, 1H, Ar-H), 6.44 (d, *J* = 6.0 Hz, 1H, Ar-H), 6.24 (s, 1H, Ar-H), 4.74 (s, 1H, NH), 3.67 (s, 3H, OCH_3_), 3.31 (brs, 2H, NH-CH_2_-), 2.98 (t, *J* = 6.0 Hz, 2H, CH_2_NHSO_2_), 1.65 (brs, 2H, -CH_2_-); ^13^C-NMR (75 MHz, CDCl_3_) δ 159.6, 158.8, 147.2, 142.5, 140.1, 139.5, 139.3, 131.0, 130.8, 130.7, 129.9, 128.8, 127.6, 125.1, 122.7, 122.7, 120.0, 119.5, 119.2, 115.7, 114.8, 114.4, 114.1, 112.1, 105.9 (Ar-C), 55.2 (OCH_3_), 40.1 (CH_2_), 38.2(CH_2_), 29.9 (CH_2_); LC-MS (*m*/*z*) calculated for C_30_H_28_ FN_5_O_3_S: 557.19, found: 558.0 (M + 1)^+^.

*N-(3-((4-(3-(3-Methoxyphenyl)-1-phenyl-1H-pyrazol-4-yl)pyridin-2-yl)amino)propyl)naphthalene-1-sulfonamide* (**2i**); White solid (66%); mp 134–136 °C; ^1^H-NMR (300 MHz, CDCl_3_) δ 8.40 (s, 1H, Ar-H), 7.97-7.78 (m, 5H, Ar-H), 7.60 (d, *J* = 6.0 Hz, 2H, Ar-H), 730-7.22 (m, 6H, Ar-H), 6.91 (d, *J* = 9.0 Hz, 1H, Ar-H), 6.77 (d, *J* = 6.0 Hz, 1H, Ar-H), 6.70 (s, 1H, Ar-H), 6.45 (d, *J* = 6.0 Hz, 1H, Ar-H), 6.20 (s, 1H, Ar-H), 4.61 (s, 1H, NH), 3.65 (s, 3H, OCH_3_), 3.31 (brs, 2H, NH-CH_2_-), 3.00 (s, 2H, CH_2_NHSO_2_), 1.64 (brs, 2H, -CH_2_-); ^13^C-NMR (75 MHz, CDCl_3_) δ 159.6, 158.8, 147.5, 142.0, 140.1, 139.6, 139.4, 137.2, 134.6, 132.1, 131.0, 129.9, 129.3, 129.1, 128.8, 128.5, 128.1, 127.8, 127.6, 127.4, 125.1, 122.7, 122.4, 120.0, 115.7, 114.8, 112.1, 105.8 (Ar-C), 55.2 (OCH_3_), 40.1 (CH_2_), 38.2 (CH_2_), 29.9 (CH_2_); LC-MS (*m*/*z*) calculated for C_30_H_28_ FN_5_O_3_S: 589.21, found: 590.0 (M + 1)^+^.

### 3.6. General Procedure for Synthesis of N-(2-((4-(3-(3-hydroxyphenyl)-1-phenyl-1H-pyrazol-4-yl)pyridin-2-yl)amino)ethyl)benzenesulfonamide *(**3a**)*, N-(2-((4-(3-(3-hydroxyphenyl)-1-phenyl-1H-pyrazol-4-yl)pyridin-2-yl)amino)ethyl)benzenesulfonamide *(**3b***–***h**)*, N-(3-((4-(3-(3-hydroxyphenyl)-1-phenyl-1H-pyrazol-4-yl)pyridin-2-yl) amino)propyl)benzenesulfonamide *(**4a**)* and N-(3-((4-(3-(3-hydroxyphenyl) -1-phenyl-1H-pyrazol-4-yl)pyridin-2-yl)amino)propyl) (substituted)benzenesulfonamide *(**4b***–***h**)*

To a mixture of compound (**1a**–**i**) or (**2a**–**i**) (0.1 mmol) in methylene chloride (5 mL), BBr_3_ (0.13 g, 1.0 mmol) was added dropwise at −78 °C under nitrogen, and the reaction mixture was stirred at 0 °C for 24 h. The mixture was quenched with saturated aqueous NaHCO_3_. Ethyl acetate (10 mL) was added and the organic layer was separated. The aqueous layer was extracted with ethyl acetate (3 × 10 mL). The combined organic layer extracts were washed with brine and dried over anhydrous Na_2_SO_4_. The organic solvent was evaporated under reduced pressure and the residue was purified by column chromatography.

*N-(2-((4-(3-(3-Hydroxyphenyl)-1-phenyl-1H-pyrazol-4-yl)pyridin-2-yl)amino)ethyl)benzenesulfonamide* (**3a**); light brown solid (36%); mp 100–102 °C; ^1^H-NMR (400 MHz, CD_3_OD) δ 8.05 (s, 1H, Ar-H), 7.83 (d, *J* = 7.2 Hz, 2H, Ar-H), 7.76 (d, *J* = 5.6 Hz, 1H, Ar-H), 7.54–7.50 (m, 3H, Ar-H), 7.37–7.34 (m, 3H, Ar-H), 7.29–7.27 (m, 2H, Ar-H), 7.18 (t, *J* = 8.0 Hz, 1H, Ar-H), 6.84-6.81 (m, 1H, Ar-H), 6.67–6.64 (m, 2H, Ar-H), 6.46 (dd, *J* = 5.2 Hz, *J* = 1.2 Hz, 1H, Ar-H), 6.38 (s, 1H, Ar-H), 3.26 (t, *J* = 6.0 Hz, 2H, NH-CH_2_-), 2.99 (t, *J* = 6.0 Hz, 2H, -CH_2_NHSO_2_), ^13^C-NMR (100 MHz, CD_3_OD) δ 158.6, 157.6, 149.5, 141.9, 140.3, 139.3, 138.9, 132.1, 130.7, 129.7, 128.7, 128.5, 127.7, 126.5, 121.2, 119.7, 116.8, 115.9, 112.3, 105.7 (Ar-C), 42.2 (CH_2_), 40.9 (CH_2_); LC-MS (*m*/*z*) calculated for C_28_H_25_N_5_O_3_S: 511.17, found: 512.0 (M + 1)^+^.

*4-Bromo-N-(2-((4-(3-(3-hydroxyphenyl)-1-phenyl-1H-pyrazol-4-yl)pyridin-2-yl)amino)ethyl)benzenesulfonamide* (**3b**); Buff solid (30%); mp 178–180 °C; ^1^H-NMR (400 MHz, CDCl_3_) δ 7.88 (s, 1H, Ar-H), 7.56 (d, *J* = 6.0 Hz, 3H, Ar-H), 7.42 (d, *J* = 8.1 Hz, 2H, Ar-H), 7.17–7.08 (m, 6H, Ar-H), 6.80 (d, *J* = 7.6 Hz, 1H, Ar-H), 6.64 (s, 1H, Ar-H), 6.58 (d, *J* = 7.1 Hz, 1H, Ar-H), 6.37 (d, *J* = 4.7 Hz, 1H, Ar-H), 6.17 (s, 1H, Ar-H), 5.08 (brs, 1H, NH), 3.13 (brs, 2H, NH-CH_2_-), 2.93 (brs, 2H, -CH_2_NHSO_2_); ^13^C-NMR (100 MHz, CD_3_OD) δ 158.0, 157.3, 146.6, 146.4, 142.4, 140.6, 139.1, 138.7, 132.3, 132.1, 130.7, 129.0, 128.5, 128.3, 127.4, 125.2, 124.9, 119.6, 117.4, 117.2, 116.2, 112.2, 105.4 (Ar-C), 42.9 (CH_2_), 41.5 (CH_2_); LC-MS (*m*/*z*) calculated for C_28_H_24_BrN_5_O_3_S: 589.17, found: 590.0 (M + 1)^+^.

*4-Chloro-N-(2-((4-(3-(3-hydroxyphenyl)-1-phenyl-1H-pyrazol-4-yl)pyridin-2-yl)amino)ethyl)benzenesulfonamide* (**3c**); Light brown solid (41%); mp 100–102 °C; ^1^H-NMR (400 MHz, CD_3_OD) δ 8.05 (s, 1H, Ar-H), 7.78–7.74 (m, 3H, Ar-H), 7.47 (d, *J* = 8.8 Hz, 2H, Ar-H), 7.35–7.32 (m 3H, Ar-H), 7.28–7.27 (m, 2H, Ar-H), 7.18 (t, *J* = 8.0 Hz, 1H, Ar-H), 6.84–6.81 (m, 1H, Ar-H), 6.67 (s, 1H, Ar-H), 6.66 (t, *J* = 2.0 Hz, 1H, Ar-H), 6.47 (dd, *J* = 5.2 Hz, *J* = 1.2 Hz, 1H, Ar-H), 6.34 (s, 1H, Ar-H), 3.25 (t, *J* = 6.0 Hz, 2H, NH-CH_2_-), 3.01 (t, *J* = 6.0 Hz, 2H, -CH_2_NHSO_2_), ^13^C-NMR (100 MHz, CD_3_OD) δ 158.5, 157.6, 146.5, 1141.9, 14.0.9, 139.3, 139.1, 138.9, 138.2, 130.7, 129.8, 129.2, 129.0, 128.9, 128.5, 128.2, 127.7, 126.2, 121.2, 119.7, 116.9, 115.9, 111.3, 105.6 (Ar-C), 42.2 (CH_2_), 40.8 (CH_2_); LC-MS (*m*/*z*) calculated for C_28_H_24_ClN_5_O_3_S: 545.13, found: 546.0 (M + 1)^+^.

*4-Fluoro-N-(2-((4-(3-(3-hydroxyphenyl)-1-phenyl-1H-pyrazol-4-yl)pyridin-2-yl)amino)ethyl)benzenesulfonamide* (**3d**); Light yellow solid (40%); mp 106–108 °C; ^1^H-NMR (400 MHz, CDCl_3_) δ 7.90 (s, 1H, Ar-H), 7.78–7.75 (m, 2H, Ar-H), 7.60 (d, *J* = 8.0 Hz, 1H, Ar-H), 7.20 (s, 5H, Ar-H), 7.12 (t, *J* = 8.0 Hz, 1H, Ar-H), 7.03 (t, *J* = 8.0 Hz, 2H, Ar-H), 6.83 (d, *J* = 8.0 Hz, 1H, Ar-H), 6.67 (s, 1H, Ar-H), 6.61 (d, *J* = 8.0 Hz, 1H, Ar-H), 6.40 (d, *J* = 8.0 Hz, 1H, Ar-H), 6.22 (s, 1H, Ar-H) 3.18 (s, 2H, NH-CH_2_-), 2.97 (s, 2H, -CH_2_NHSO_2_), ^13^C-NMR (100 MHz, CDCl_3_) δ 166.1, 163.6, 158.1, 157.3, 146.6, 142.4, 140.6, 139.2, 135.7, 130.7, 130.2, 129.7, 129.6, 128.8, 127.8, 125.1, 121.7, 119.6, 116.7, 116.3, 116.1, 112.3, 105.5 (Ar-C), 42.9 (CH_2_), 41.6 (CH_2_); LC-MS (*m*/*z*) calculated for C_28_H_24_FN_5_O_3_S: 529.59, found: 530.0 (M + 1)^+^.

*N-(2-((4-(3-(3-Hydroxyphenyl)-1-phenyl-1H-pyrazol-4-yl)pyridin-2-yl)amino)ethyl)-4-methylbenzenesulfonamide* (**3e**); Yellow solid (38%); mp 114–116 °C; ^1^H-NMR (400 MHz, CDCl_3_) δ 7.91 (s, 1H, Ar-H), 7.69 (d, *J* = 8.0 Hz, 3H, Ar-H), 7.29–7.21 (m, 6H, Ar-H), 7.15 (t, *J* = 8.0 Hz, 1H, Ar-H), 6.87 (d, *J* = 8.0 Hz, 1H, Ar-H), 6.72 (s, 1H, Ar-H), 6.61 (d, *J* = 8.0 Hz, 1H, Ar-H), 6.44 (s, 1H, Ar-H), 6.22 (s, 1H, Ar-H), 5.32 (brs, 1H, NH), 3.20 (brs, 2H, NH-CH_2_-), 3.00–2.98 (m, 2H, -CH_2_NHSO_2_), 2.78 (s, 3H, CH_3_); ^13^C-NMR (100 MHz, CDCl_3_) δ 158.2, 157.1, 146.7, 143.4, 142.4, 140.4, 139.3, 139.2, 136.7, 130.8, 130.2, 129.7, 128.8, 127.6, 127.0, 125.0, 121.8, 119.7, 116.8, 112.3, 105.6 (Ar-C), 42.9 (CH_2_), 41.6 (CH_2_), 21.0(CH_3_); LC-MS (*m*/*z*) calculated for C_29_H_29_N_5_O_3_S: 525.59, found: 526.0 (M + 1)^+^.

*N-(2-((4-(3-(3-Hydroxyphenyl)-1-phenyl-1H-pyrazol-4-yl)pyridin-2-yl)amino)ethyl)-4-(trifluoromethyl)benzenesulfonamide* (**3f**); White solid (40%); mp 138–140 °C; ^1^H-NMR (400 MHz, CDCl_3_) δ 7.86 (d, *J* = 9.0 Hz, 3H, Ar-H), 7.62–7.57 (m, 3H, Ar-H), 7.17 (s, 5H, Ar-H), 7.10 (t, *J* = 7.6 Hz, 1H, Ar-H), 6.80 (d, *J* = 7.1 Hz, 1H, Ar-H), 6.64 (s, 1H, Ar-H), 6.58 (d, *J* = 7.0 Hz, 1H, Ar-H), 6.38 (s, 1H, Ar-H), 5.32 (brs, 1H, NH), 3.20 (brs, 2H, NH-CH_2_-), 3.00–2.98 (m, 2H, -CH_2_NHSO_2_), 2.78 (s, 3H, CH_3_); LC-MS (*m*/*z*) calculated for C_29_H_24_F_3_N_5_O_3_S: 579.59, found: 580.0 (M + 1)^+^.

*3-Fluoro-N-(2-((4-(3-(3-hydroxyphenyl)-1-phenyl-1H-pyrazol-4-yl)pyridin-2-yl)amino)ethyl)benzenesulfonamide* (**3g**); White solid (32%); mp 146–148 °C; ^1^H-NMR (400 MHz, CD_3_OD) δ 8.05 (s, 1H, Ar-H), 7.87 (d, *J* = 5.2 Hz, 1H, Ar-H), 7.64 (d, *J* = 7.6 Hz, 1H, Ar-H), 7.56–7.51 (m, 2H, Ar-H), 7.37–7.28 (m, 6H, Ar-H), 7.19 (t, *J* = 8.0 Hz, 1H, Ar-H), 6.82 (d, *J* = 8.0 Hz, 1H, Ar-H), 6.68–6.65 (m, 2H, Ar-H), 6.47 (d, *J* = 5.6 Hz, 1H, Ar-H), 6.37 (s, 1H, Ar-H), 3.27 (t, *J* = 5.2 Hz, 2H, NH-CH_2_-), 3.02 (t, *J* = 6.0 Hz, 2H, -CH_2_NHSO_2_); ^13^C-NMR (75 MHz, CD_3_OD) δ 163.6, 161.1, 158.6, 157.6, 146.6, 142.6, 141.9, 139.3, 138.9, 130.8, 130.0, 130.7, 129.7, 128.5, 127.7, 125.2, 122.5, 121.1, 119.7, 119.1, 118.8, 116.8, 113.7, 113.5, 111.3, 105.7 (Ar-C), 42.2 (CH_2_), 40.8 (CH_2_); LC-MS (*m*/*z*) calculated for C_28_H_24_ FN_5_O_3_S: 529.16, found: 530.0 (M + 1)^+^.

*N-(2-((4-(3-(3-Hydroxyphenyl)-1-phenyl-1H-pyrazol-4-yl)pyridin-2-yl)amino)ethyl)naphthalene-1-sulfonamide* (**3h**); White solid (33%); mp 146–148 °C; ^1^H-NMR (400 MHz, CDCl_3_) δ 8.37 (s, 1H, Ar-H), 7.87–7.73 (m, 5H, Ar-H), 7.61–7.51 (m, 3H, Ar-H), 7.22 (d, *J* = 4.0 Hz, 5H, Ar-H), 7.11 (t, *J* = 8.0 Hz, 1H, Ar-H), 6.84 (dd, *J* = 8.0 Hz, *J* = 4.0 Hz, 1H, Ar-H), 6.69 (t, *J* = 4.0 Hz, 1H, Ar-H), 6.58 (d, *J* = 8.0 Hz, 1H, Ar-H), 6.38 (dd, *J* = 8.0 Hz, *J* = 4.0 Hz, 1H, Ar-H), 6.15 (s, 1H, Ar-H), 5.03 (brs, 1H, NH), 3.19 (brs, 2H, NH-CH_2_-), 3.02 (t, *J* = 8.0 Hz, 2H, -CH_2_NHSO_2_); ^13^C-NMR (75 MHz, CDCl_3_) δ 158.0, 157.2, 146.5, 142.4, 140.5, 139.2, 136.4, 134.7, 132.0, 130.7, 130.2, 129.4, 129.2, 128.8, 128.3, 127.8, 127.7, 127.4, 125.0, 122.1, 121.7, 119.6, 117.3, 116.8, 112.3, 105.5 (Ar-C), 43.0 (CH_2_), 41.6 (CH_2_); LC-MS (*m*/*z*) calculated for C_32_H_27_N_5_O_3_S: 561.66, found: 562.0 (M + 1)^+^.

*N-(3-((4-(3-(3-Hydroxyphenyl)-1-phenyl-1H-pyrazol-4-yl)pyridin-2-yl)amino)propyl)benzenesulfonamide* (**4a**); White solid (37%); mp 154–156 °C; ^1^H-NMR (400 MHz, CDCl_3_) δ 7.92 (s, 1H, Ar-H), 7.81–7.74 (m, 3H, Ar-H), 7.51 (d, *J* = 7.2 Hz, 1H, Ar-H), 7.44 (t, *J* = 7.6 Hz, 2H, Ar-H), 7.26 (s, 5H, Ar-H), 7.14 (t, *J* = 7.9 Hz, 1H, Ar-H), 6.87 (d, *J* = 7.88 Hz, 1H, Ar-H), 6.74 (s, 1H, Ar-H), 6.60 (d, *J* = 7.4 Hz, 1H, Ar-H), 6.51 (d, *J* = 5.3 Hz, 1H, Ar-H), 6.11(s, 1H, Ar-H), 4.83 (brs, 1H, NH), 3.06 (brs, 2H, NH-CH_2_-), 2.88 (d, *J* = 5.8 Hz, 2H, -CH_2_NHSO_2_), 1.53 (p, *J* = 5.8 Hz, 2H, CH_2_CH_2_CH_2_); LC-MS (*m*/*z*) calculated for C_29_H_27_N_5_O_3_S: 525.63, found: 526.0 (M + 1)^+^.

*4-Bromo-N-(3-((4-(3-(3-hydroxyphenyl)-1-phenyl-1H-pyrazol-4-yl)pyridin-2-yl)amino)propyl)benzenesulfonamide* (**4b**); Light yellow solid (42%); mp 184–186 °C; ^1^H-NMR (400 MHz, CD_3_OD) δ 8.06 (s, 1H, Ar-H), 7.79 (d, *J* = 5.2 Hz, 1H, Ar-H), 7.80–7.67 (m, 4H, Ar-H), 7.37–7.34 (m, 3H, Ar-H), 7.29–7.27 (m, 2H, Ar-H), 7.19 (t, *J* = 8.4 Hz, 1H, Ar-H), 6.84–6.81 (m, 1H, Ar-H), 6.68 (s, 1H, Ar-H), 6.66 (s, 1H, Ar-H), 6.51 (dd, *J* = 5.6 Hz, *J* = 1.2 Hz, 1H, Ar-H), 6.34 (s, 1H, Ar-H), 3.14 (t, *J* = 8.0 Hz, 2H, NH-CH_2_-), 2.93 (t, *J* = 8.0 Hz, 2H, -CH_2_NHSO_2_); 1.61 (d, *J* = 6.0 Hz, 2H, CH_2_CH_2_CH_2_); ^13^C-NMR (100 MHz, CD_3_OD) δ 158.9, 157.6, 146.6, 141.9, 140.9, 139.7, 139.3, 138.9, 132.0, 130.8, 129.8, 128.5, 128.3, 127.7, 126.6, 125.2, 121.2, 119.8, 116.8, 115.9, 110.9, 105.4 (Ar-C), 40.2 (CH_2_), 38.1 (CH_2_), 28.8 (CH_2_); LC-MS (*m*/*z*) calculated for C_29_H_26_BrN_5_O_3_S: 604.10, found: 605.0 (M + 1)^+^.

*4-Chloro-N-(3-((4-(3-(3-hydroxyphenyl)-1-phenyl-1H-pyrazol-4-yl)pyridin-2-yl)amino)propyl)benzenesulfonamide* (**4c**); White solid (52%); mp 166–168 °C; ^1^H-NMR (400 MHz, CD_3_OD) δ 8.06 (s, 1H, Ar-H), 7.87–7.75 (m, 3H, Ar-H), 7.60–7.46 (m, 2H, Ar-H), 7.36 (t, *J* = 5.8 Hz, 3H, Ar-H), 7.32–7.26 (m, 2H, Ar-H), 7.19 (t, *J* = 7.9 Hz, 1H, Ar-H), 6.83 (ddd, *J* = 8.3 Hz, 2.3 Hz, 0.8 Hz, 1H, Ar-H), 6.67 (t, *J* = 5.0 Hz, 2H, Ar-H), 6.51 (dd, *J* = 5.5 Hz, 1.4 Hz, 1H, Ar-H), 6.34 (s, 1H, Ar-H), 3.14 (t, *J* = 6.6 Hz, 2H, NH-CH_2_-), 2.93 (t, *J* = 6.8 Hz, 2H, -CH_2_NHSO_2_), 1.61 (p, *J* = 6.7 Hz, 2H, CH_2_CH_2_CH_2_); ^13^C-NMR (100 MHz, CD_3_OD) δ 158.9, 157.6, 146.7, 141.9, 140.9, 139.3, 139.2, 138.9, 138.3, 130.8, 129.8, 129.0, 128.5, 128.2, 127.7, 125.2, 121.2, 119.8, 116.8, 115.9, 110.9, 105.4 (Ar-C), 40.2 (CH_2_), 38.1 (CH_2_), 28.8 (CH_2_); LC-MS (*m*/*z*) calculated for C_29_H_26_ClN_5_O_3_S: 560.7, found: 561.0 (M + 1)^+^.

*4-Fluoro-N-(3-((4-(3-(3-hydroxyphenyl)-1-phenyl-1H-pyrazol-4-yl)pyridin-2-yl)amino)propyl)benzenesulfonamide* (**4d**); White solid (45%); mp 144–146 °C; ^1^H-NMR (400 MHz, CD_3_OD) δ 8.06 (s, 1H, Ar-H), 7.98–7.86 (m, 2H, Ar-H), 7.80 (d, *J* = 5.5 Hz, 1H, Ar-H), 7.37 (d, *J* = 6.6 Hz, 3H, Ar-H), 7.33–7.24 (m, 4H, Ar-H), 7.20 (t, *J* = 7.8 Hz, 1H, Ar-H), 6.83 (d, *J* = 8.2 Hz, 1H, Ar-H), 6.67 (d, *J* = 8.4 Hz, 2H, Ar-H), 6.51 (d, *J* = 5.5 Hz, 1H, Ar-H), 6.35 (s, 1H, Ar-H), 3.15 (t, *J* = 6.6 Hz, 2H, NH-CH_2_-), 2.92 (t, *J* = 6.7 Hz, 2H,-CH_2_NHSO_2_), 1.74–1.53 (m, 2H, CH_2_CH_2_CH_2_); ^13^C-NMR (100 MHz, CD_3_OD) δ 158.9, 157.6, 146.7, 141.9, 139.3, 138.8, 136.7, 130.8, 129.7, 129.5, 129.4, 128.5, 127.7, 125.2, 121.2, 119.8, 116.8, 115.9, 115.8, 115.6, 110.9, 105.4 (Ar-C), 40.2 (CH_2_), 38.2 (CH_2_), 28.8 (CH_2_); LC-MS (*m*/*z*) calculated for C_29_H_26_FN_5_O_3_S: 543.17, found: 544.0 (M + 1)^+^.

*N-(3-((4-(3-(3-Hydroxyphenyl)-1-phenyl-1H-pyrazol-4-yl)pyridin-2-yl)amino)propyl)-4-methylbenzenesulfonamide* (**4e**); White solid (33%); mp 158–160 °C; ^1^H-NMR (400 MHz, CD_3_OD) δ 8.06 (s, 1H, Ar-H), 7.79 (d, *J* = 5.5 Hz, 1H, Ar-H), 7.72 (d, *J* = 8.3 Hz, 2H, Ar-H), 7.41–7.25 (m, 7H, Ar-H), 7.19 (t, *J* = 7.9 Hz, 1H, Ar-H), 6.83 (ddd, *J* = 8.3 Hz, 2.3 Hz, 1.0 Hz, 1H, Ar-H), 6.71–6.61 (m, 2H, Ar-H), 6.51 (dd, *J* = 5.5, 1.5 Hz, 1H, Ar-H), 6.34 (s, 1H, Ar-H), 3.13 (t, *J* = 6.7 Hz, 2H, NH-CH_2_-), 2.89 (t, *J* = 6.8 Hz, 2H,-CH_2_NHSO_2_), 2.41 (s, 3H, CH_3_), 1.60 (p, *J* = 6.7 Hz, 2H, CH_2_CH_2_CH_2_); ^13^C-NMR (100 MHz, CD_3_OD) δ 158.9, 157.6, 146.7, 143.1, 141.9, 140.9, 139.3, 138.9, 137.4, 130.8, 129.8, 129.3, 128.5, 127.7, 126.6, 125.2, 121.2, 119.8, 116.8, 115.9, 110.9, 105.4 (Ar-C), 40.2 (CH_2_), 38.2 (CH_2_), 28.9 (CH_2_), 20.1 (CH_3_); LC-MS (*m*/*z*) calculated for C_30_H_29_N_5_O_3_S: 539.20, found: 540.0 (M + 1)^+^.

*N-(3-((4-(3-(3-Hydroxyphenyl)-1-phenyl-1H-pyrazol-4-yl)pyridin-2-yl)amino)propyl)-4-(trifluoromethyl)benzenesulfonamide* (**4f**); White solid (39%); mp 150–152 °C; ^1^H-NMR (400 MHz, CD_3_OD) δ 8.09–7.99 (m, 3H, Ar-H), 7.87 (d, *J* = 8.3 Hz, 2H, Ar-H), 7.80 (d, *J* = 5.5 Hz, 1H, Ar-H), 7.41–7.34 (m, 3H, Ar-H), 7.34–7.25 (m, 2H, Ar-H), 7.20 (t, *J* = 7.9 Hz, 1H, Ar-H), 6.83 (ddd, *J* = 8.3 Hz, 2.4 Hz, 0.9 Hz, 1H, Ar-H), 6.71–6.63 (m, 2H, Ar-H), 6.52 (dd, *J* = 5.6 Hz, 1.5 Hz, 1H, Ar-H), 6.36 (d, *J* = 6.0 Hz, 1H, Ar-H), 3.16 (t, *J* = 6.7 Hz, 2H, NH-CH_2_-), 2.96 (t, *J* = 6.8 Hz, 2H, -CH_2_NHSO_2_), 1.64 (p, *J* = 6.7 Hz, 2H, CH_2_CH_2_CH_2_); ^13^C-NMR (100 MHz, CD_3_OD) δ 158.8, 157.6, 146.5, 142.0, 140.9, 139.3, 138.8, 130.7, 129.7, 128.5, 127.7, 127.3, 125.9, 125.2, 121.2, 119.7, 116.8, 115.9, 110.9, 105.5 (Ar-H), 40.2 (CH_2_), 38.1 (CH_2_), 28.9 (CH_2_); LC-MS (*m*/*z*) calculated for C_30_H_26_F_3_N_5_O_3_S: 593.20, found: 594.0 (M + 1)^+^.

*3-Fluoro-N-(3-((4-(3-(3-hydroxyphenyl)-1-phenyl-1H-pyrazol-4-yl)pyridin-2-yl)amino)propyl)benzenesulfonamide* (**4g**); White solid (41%); mp 120–122 °C; ^1^H-NMR (400 MHz, CD_3_OD) δ 8.05 (s, 1H, Ar-H), 7.79 (d, *J* = 5.5 Hz, 1H, Ar-H), 7.67 (d, *J* = 7.9 Hz, 1H, Ar-H), 7.63–7.51 (m, 2H, Ar-H), 7.41–7.25 (m, 6H, Ar-H), 7.19 (t, *J* = 7.9 Hz, 1H, Ar-H), 6.87–6.79 (m, 1H, Ar-H), 6.67 (t, *J* = 4.2 Hz, 2H, Ar-H), 6.50 (dd, *J* = 5.5 Hz, 1.3 Hz, 1H, Ar-H), 6.36 (s, 1H, Ar-H), 3.15 (t, *J* = 6.7 Hz, 2H, NH-CH_2_-), 2.93 (t, *J* = 6.8 Hz, 2H, -CH_2_NHSO_2_), 1.63 (p, *J* = 6.7 Hz, 2H, CH_2_CH_2_CH_2_), ^13^C-NMR (100 MHz, CD_3_OD) δ 163.7, 161.2, 158.8, 157.6, 146.5, 142.7, 141.9, 140.9, 139.3, 138.9, 130.9, 130.8, 130.8, 129.8, 128.5, 127.7, 125.2, 122.5, 122.5, 121.2, 119.8, 119.1, 118.9, 116.8, 115.9, 113.7, 113.5, 110.9, 105.5 (Ar-C), 40.2 (CH_2_), 38.2 (CH_2_), 28.9 (CH_2_); LC-MS (*m*/*z*) calculated for C_29_H_26_FN_5_O_3_S: 543.17, found: 544.0 (M + 1)^+^.

*N-(3-((4-(3-(3-Hydroxyphenyl)-1-phenyl-1H-pyrazol-4-yl)pyridin-2-yl)amino)propyl)naphthalene-1-sulfonamide* (**4h**); White solid (40%); mp 168–70 °C; ^1^H-NMR (400 MHz, CD_3_OD) δ 8.40 (s, 1H, Ar-H), 8.01 (s, 1H, Ar-H), 7.97 (d, *J* = 8.4 Hz, 2H, Ar-H), 7.92 (d, *J* = 8.0 Hz, 1H, Ar-H), 7.81 (dd, *J* = 8.8 Hz, 1.6 Hz, 1H, Ar-H), 7.73 (d, *J* = 5.2 Hz, 1H, Ar-H), 7.65-7.57 (m, 2H, Ar-H), 7.36-7.25 (m, 5H, Ar-H), 7.13 (t, *J* = 8.0 Hz, 1H, Ar-H), 6.79 (dd, *J* = 8.4 Hz, 2.0 Hz, 1H, Ar-H), 6.62 (s, 1H, Ar-H), 6.59 (d, *J* = 6.4 Hz, 1H, Ar-H), 6.46 (d, *J* = 4.8 Hz, 1H, Ar-H), 6.27 (s, 1H, Ar-H), 3.11 (t, *J* = 6.8 Hz, 2H), 2.94 (t, *J* = 6.8 Hz, 2H, Ar-H), 1.60 (t, *J* = 6.8Hz, 2H, Ar-H); ^13^C-NMR (100 MHz, CD_3_OD) δ 158.7, 157.6, 146.4, 141.9, 140.9, 139.3, 138.9, 137.2, 134.7, 132.1, 130.7, 129.7, 129.1, 128.8, 128.5, 128.3, 127.7, 127.6, 127.5, 127.2, 125.2, 122.0, 121.2, 119.7, 116.8, 115.9, 110.9, 105.3 (Ar-C), 40.2 (CH_2_), 38.2 (CH_2_), 28.8 (CH_2_); LC-MS (*m*/*z*) calculated for C_33_H_29_N_5_O_3_S: 575.20, found: 576.0 (M + 1)^+^.

### 3.7. Biological Evaluation

Cell culture and sample treatment were performed as reported in the literature [38,39,40,41]. The 3-(4,5-dimethylthiazol-2-yl)-2,5-diphenyltetrazolium bromide (MTT) assay for cell viability followed the procedure previously described in the literature [38,39,40,41]. Nitrite determination was carried as described in the literature [38,39,40,41], and the PGE_2_ assay was carried as previously described [38,39,40,41].

## 4. Conclusion

In this article, a new series of 1,3,4-triaylpyrazole derivatives were synthesized. The new analogues were divided into four groups **1a**–**i**, **2a**–**i**, **3a**–**h**, and **4a**–**h**. All compounds were tested for their ability to inhibit nitric oxide production in LPS-induced RAW 264.7 macrophages and cell viability to measure their cytotoxic effects. Compounds **1a**–**i** exhibited the highest NO production inhibitor activity with low toxicity profile, followed by **2a**–**I**, then **4a**–**h**, and finally **3a**–**h**. Compounds **3a**–**h** and **4a**–**h** showed high cellular toxicity. Compounds **1b**, **1d**, **1g**, **2a**, and **2c** had the highest activity and lowest toxicity. Compounds **1b**, **1d**, **1g**, **2a**, and **2c** were tested for their PGE_2_ inhibition ability and showed IC_50_ values of 4.72, 5.06, 4.55, 4.87, and 4.68 µM, respectively. Compounds **1b**, **1d**, **1g**, **2a**, and **2c** were assayed for their ability to inhibit iNOS and COX-2 expressions. Compounds **1b**, **1d**, and **1g** exhibited a potential iNOS inhibitory effect at 20 µM and slightly inhibited iNOS enzyme activity in a dose-dependent manner. We concluded that these compounds inhibit NO production by inhibiting iNOS protein expression and by inhibiting iNOS enzyme activity to a lesser extent. These compounds with good activity and relatively low toxicity profiles can be used as promising compounds for future optimization and development of potential anti-inflammatory agents. 

## Figures and Tables

**Table 1:** Structures and yields of the final target compounds.

Compound	n	R^1^	R^2^	Yield	Compound	n	R^1^	R^2^	Yield
**1a**	1	CH_3_	H	65%	**3a**	1	H	H	**36%**
**1b**	1	CH_3_	4-Br	61%	**3b**	1	H	4-Br	**30%**
**1c**	1	CH_3_	4-Cl	60%	**3c**	1	H	4-Cl	**41%**
**1d**	1	CH_3_	4-F	67%	**3d**	1	H	4-F	**40%**
**1e**	1	CH_3_	p-OCH_3_	62%	**3e**	1	H	4-CH_3_	**38%**
**1f**	1	CH_3_	4-CH_3_	69%	**3f**	1	H	4-CF_3_	**40%**
**1g**	1	CH_3_	4-CF_3_	74%	**3g**	1	H	3-F	**32%**
**1h**	1	CH_3_	3-F	66%	**3h**	1	H	Fused benzene	**33%**
**1i**	1	CH_3_	Fused benzene	71%	**4a**	2	H	H	**37%**
**2a**	2	CH_3_	H	60%	**4b**	2	H	4-Br	**42%**
**2b**	2	CH_3_	4-Br	62%	**4c**	2	H	4-Cl	**52%**
**2c**	2	CH_3_	4-Cl	62%	**4d**	2	H	4-F	**43%**
**2d**	2	CH_3_	4-F	75%	**4e**	2	H	4-CH_3_	**33%**
**2e**	2	CH_3_	4-OCH3	71%	**4f**	2	H	4-CF_3_	**39%**
**2f**	2	CH_3_	4-CH_3_	72%	**4g**	2	H	3-F	**41%**
**2g**	2	CH_3_	4-CF_3_	71%	**4h**	2	H	Fused benzene	**40%**
**2h**	2	CH_3_	3-F	76%					
**2i**	2	CH_3_	Fused benzene	66%					

**Table 2:** Nitric oxide production inhibition of the final target compounds at different dose levels.

Compound	Nitric Oxide % Inhibition		
	1 µM	5 µM	10 µM
**1a**	17.35 ± 0.09	35.36 ± 1.14	52.93 ± 3.12
**1b**	12.05 ± 0.11	24.23 ± 0.98	68.66 ± 2.47
**1c**	13.27 ± 0.08	24.76 ± 0.17	49.89 ± 1.25
**1d**	19.79 ± 0.14	29.36 ± 0.77	61.28 ± 1.33
**1e**	13.52 ± 0.11	16.10 ± 0.09	41.47 ± 0.99
**1f**	16.29 ± 0.10	26.54 ± 0.65	53.09 ± 2.10
**1g**	10.98 ± 0.07	23.62 ± 0.54	60.80 ± 1.75
**1h**	11.12 ± 0.02	26.24 ± 0.99	41.25 ± 2.15
**1i**	13.40 ± 0.18	19.90 ± 0.24	51.51 ± 0.14
**2a**	0.17 ± 0.03	30.20 ± 0.27	62.76 ± 3.25
**2b**	0.01 ± 0.03	28.06 ± 0.66	52.44 ± 1.62
**2c**	0.02 ± 0.01	24.69 ± 0.81	59.09 ± 0.93
**2d**	7.81 ± 0.03	23.03 ± 0.89	55.00 ± 4.01
**2e**	2.02 ± 0.01	23.90 ± 1.12	50.20 ± 1.88
**2f**	4.36 ± 0.04	19.76 ± 0.96	46.32 ± 3.21
**2g**	2.91 ± 0.02	26.26 ± 1.01	56.03 ± 0.89
**2h**	4.87 ± 0.05	24.07 ± 0.91	41.54 ± 1.12
**2i**	16.15 ± 0.12	25.75 ± 0.49	51.93 ± 1.93
**3a**	4.78 ± 0.03	21.49 ± 0.37	48.97 ± 1.10
**3b**	15.82 ± 0.16	24.36 ± 0.52	48.69 ± 1.74
**3c**	6.34 ± 0.06	11.68 ± 0.71	33.81 ± 0.89
**3d**	10.58 ± 0.09	18.31 ± 0.26	48.18 ± 0.74
**3e**	7.15 ± 0.04	12.40 ± 0.72	31.43 ± 0.33
**3f**	6.08 ± 0.07	15.21 ± 0.91	36.51 ± 0.59
**3g**	16.19 ± 0.17	19.06 ± 0.89	37.68 ± 0.85
**3h**	10.33 ± 0.08	14.72 ± 0.42	16.07 ± 0.48
**4a**	11.11 ± 0.10	24.13 ± 0.56	59.25 ± 1.31
**4b**	8.19 ± 0.06	22.18 ± 0.72	63.82 ± 2.14
**4c**	0.86 ± 0.09	22.02 ± 0.42	46.95 ± 1.34
**4d**	9.02 ± 0.03	20.84 ± 0.36	48.49 ± 1.79
**4e**	0.69 ± 0.01	18.57 ± 0.98	59.69 ± 0.70
**4f**	4.87 ± 0.14	18.87 ± 1.02	53.82 ± 1.87
**4g**	ND	29.49 ± 0.86	51.15 ± 1.45
**4h**	0.97± 0.01	21.04 ± 0.22	50.21 ± 2.01
**l-NIL (40 μM)**	77.89 ± 4.25		

ND: Not determined.

**Table 3:** IC_50_ (μM) for nitric oxide production and cell viability of final target compounds.

Compound	NO( IC_50_) ^a^	Cytotoxicity (IC_50_) ^a^
**1a**	9.17 ± 0.52	245.78 ± 1.91
**1b**	7.90 ± 0.41	254.15 ± 2.54
**1c**	10.10 ± 0.63	219.39 ± 0.14
**1d**	8.23 ± 0.32	169.15 ± 1.64
**1e**	13.40 ± 0.72	285.41 ± 4.18
**1f**	9.42 ± 0.12	291.01 ± 1.12
**1g**	8.55 ± 0.14	261.57 ± 1.57
**1h**	12.62 ± 0.29	244.21 ± 2.23
**1i**	9.76 ± 0.21	>400
**2a**	8.04 ± 0.09	346.2 ± 4.21
**2b**	9.50 ± 0.34	384.69 ± 1.29
**2c**	8.68 ± 0.22	289.92 ± 1.87
**2d**	12.42 ± 0.40	260.32 ± 2.24
**2e**	9.96 ± 0.18	>400
**2f**	9.16 ± 0.27	322.54 ± 3.35
**2g**	10.45 ± 0.44	252.64 ± 1.87
**2h**	9.19 ± 0.25	245.78 ± 2.71
**2i**	9.63 ± 0.48	>400
**3a**	10.29 ± 0.23	29.75 ± 1.91
**3b**	10.55 ± 0.51	24.31 ± 0.41
**3c**	15.21 ± 0.17	22.69 ± 0.30
**3d**	10.72 ± 0.52	26.58 ± 0.47
**3e**	16.14 ± 0.72	32.52 ± 0.75
**3f**	22.21 ± 0.31	24.1 5 ± 1.61
**3g**	21.42 ± 0.17	28.74 ± 0.74
**3h**	>30	100.21 ± 1.21
**4a**	8.86 ± 0.36	16.58 ± 0.91
**4b**	8.34 ± 0.11	19.54 ± 0.49
**4c**	13.25 ± 0.52	18.79 ± 0.68
**4d**	11.32 ± 0.16	12.58 ± 0.22
**4e**	8.82 ± 0.32	22.96 ± 0.63
**4f**	9.45 ± 0.15	17.73 ± 0.61
**4g**	8.24 ± 0.41	9.35 ± 0.32
**4h**	9.96 ± 0.48	10.92 ± 0.22
**l-NIL**	29.32 ± 0.15	ND

^a^ Values represent means ± SD of three independent experiments; ND: Not determined.

**Table 4:** Inhibitory effect and IC_50_ values of compounds **1b**, **1d**, **1g**, **2a**, and **2c** on prostaglandin E2 (PGE_2_) production.

Compound	PGE_2_ Inhibition (%) ^a^			
	1 μM	5 μM	10 μM	IC_50_ (μM)
**1b**	16.24 ± 0.79	52.56 ± 2.21	76.92 ± 1.34	4.75 ± 0.25
**1d**	10.26 ± 0.81	49.57 ± 1.25	85.75 ± 2.64	5.06 ± 0.21
**1g**	19.94 ± 0.89	53.85 ± 1.91	79.34 ± 2.19	4.55 ± 0.39
**2a**	31.09 ± 1.14	50.70 ± 0.99	76.89 ± 4.99	4.87 ± 0.44
**2c**	17.51 ± 1.51	52.80 ± 2.01	81.37 ± 2.94	4.68 ± 0.37
**NS-398**	90.53 ± 0.53	95.78 ± 0.67	98.25 ± 1.50	6.25 × 10^−3^ ± 0.41 × 10^−3^

^a^ Values represent means ± SD of three independent experiments.

## Supplementary Materials

Spectral data of the new synthesized compounds and intermediates are available online.
