# Supplementary material for: Synthesis of New Triarylpyrazole Derivatives Possessing Terminal Sulfonamide Moiety and Their Inhibitory Effects on PGE2 and Nitric Oxide Productions in Lipopolysaccharide-Induced RAW 264.7 Macrophages
Source: Molecules. 2018 Oct 7;23(10):2556. doi: 10.3390/molecules23102556 (PMC6222820; doi:10.3390/molecules23102556)

Synthesis of new triarylpyrazole derivatives possessing terminal sulfonamide moiety and their inhibitory effects on PGE_2 and nitric oxide productions in LPS-induced RAW 264.7 macrophages

Mohammed S. Abdel-Maksoud[1], Mohammed I. El-Gamal[2,3,4], Mahmoud M. Gamal El-Din[1],Yunji Choi[5], Jungseung Choi[5], Ji-Sun Shin[6,7], Shin-Young Kang[6,7], Kyung Ho Yoo[8], Kyung-Tae Lee[6,7], Daejin Baek[5*], and Chang-Hyun Oh[9,10*]

[1] Medicinal & Pharmaceutical Chemistry Department, Pharmaceutical and Drug Industries Research Division, National Research Centre (NRC), Dokki, Giza, 12622, Egypt. E-mail: ph_ss@hotmail.com

[2] Department of Medicinal Chemistry, College of Pharmacy, University of Sharjah, Sharjah 27272, United Arab Emirates. E-mail: drmelgamal2002@gmail.com

[3] Sharjah Institute for Medical Research, University of Sharjah, Sharjah 27272, United Arab Emirates.

[4] Department of Medicinal Chemistry, Faculty of Pharmacy, University of Mansoura, Mansoura 35516, Egypt.

[5] Department of Chemistry, Hanseo University, Seosan, Republic of Korea. E-mail: djbaek@hanseo.ac.kr

[6] Department of Pharmaceutical Biochemistry, College of Pharmacy, Kyung Hee University, Seoul, Republic of Korea.

[7] Department of Life and Nanopharmaceutical Science, College of Pharmacy, Kyung Hee University, Seoul, Republic of Korea.

[8] Chemical Kinomics Research Center, Korea Institute of Science and Technology, Seoul, Republic of Korea.

[9] Center for Biomaterials, Korea Institute of Science and Technology (KIST), Cheongryang, Seoul 130-650, Republic of Korea.

[10] Department of Biomolecular Science, University of Science and Technology (UST), Daejeon, Yuseong-gu, 34113, Republic of Korea.

[*] Correspondence: Dr. Chang-Hyun Oh, Center for Biomaterials, Korea Institute of Science and Technology, PO Box 131, Cheongryang, Seoul 130-650, Republic of Korea.

E-mail: choh@kist.re.kr

Dr. Daejin Baek, Department of Chemistry, Hanseo University, Seosan 356-706, Republic of Korea.

E-mail: djbaek@hanseo.ac.kr Fax: +82-41-660-1119.

Received: date; Accepted: date; Published: date

Table of contents

Title page	1s
Table of contents	2s
Experimental	3s-5s
Representative NMR charts	6s-68s

Experimental

Synthesis of methyl benzoate

A solution of 3-methoxybenzoic (304 mg, 2.0 mmol) in methanol (5 ml) were heated under reflux until the acid was completely dissolved in methanol then few drops of concentrated sulphuric acid was added to the mixture and refluxed for 8 hr. The resulting mixture was cooled to room temperature, diluted with water and a saturated solution of sodium bicarbonate was added to the mixture to neutralize the benzoic acid, extracted with ethyl acetate, dried and evaporated to get the required ester (300 mg, 90.3%) as yellow liquid; ^{1}H NMR (400 MHz, $CDCl_3$) δ 8.15 (dd, *J* = 9.0 Hz, 2H, Ar-H), 7.49 (t, *J* = 9.0 Hz, 1H, Ar-H), 7.37 (t, *J* = 9.0 Hz, 2H, Ar-H), 3.85 (s, 3H, OCH_3 ester), 3.76 (s, 3H, OCH_3); ^{13}C NMR (100 MHz, $CDCl_3$) δ 166.8, 132.8, 130.1, 129.5, 128.2 (Ar-C), 55.2 (OCH_3), 51.8 (OCH_3 ester).

Synthesis of 2-(2-bromopyridin-4-yl)-1-(3-methoxyphenyl)ethan-1-one

To a solution of methyl benzoate (775 mg, 5.0 mmol) and 2-bromo-4-picoline (0.5 mL, 5.6 mmol) in anhydrous THF (5 mL) in a cooled bath at -25 ℃, LiHMDS (3.7 mL, 1.0M solution in THF, 19.9 mmol) was slowly added to maintain the temperature at -25 ℃. The resulting mixture was stirred overnight at room temperature. The mixture was quenched with saturated aqueous NH_4Cl. Ethyl acetate was added and the organic layer was separated. The aqueous layer was extracted with ethyl acetate (3 x10 mL). The combined organic layer extracts were washed with brine and dried over anhydrous Na_2SO_4. The organic solvent was evaporated under reduced pressure and the residue was purified by flash column chromatography (silica gel, hexane ethyl acetate 12:1 v/v then switching to hexane-ethyl acetate 10:1 v/v) to yield 2-(2-Bromopyridin-4-yl)-1-(3-methoxyphenyl) ethan-1-one (**17**) (1.0 g, 69.9 %) as light yellow solid; m.p.85-88 ℃; ^{1}H

NMR (400 MHz, $CDCl_3$) δ 8.29 (d, *J* = 5.2 Hz, 1H, Ar-H), 7.52 (d, *J* = 7.2 Hz, 1H, Ar-H), 7.56-7.48 (m, 1H, Ar-H), 7.40 (t, *J* = 8.0 Hz, 2H, Ar-H), 7.15 (m, 2H, Ar-H), 4.25 (s, 2H, CH_2), 3.84 (s, 3H, OCH_3); ^{13}C NMR (100 MHz, $CDCl_3$) δ 160.0 (C=O), 150.0, 146.5, 137.3, 129.9, 129.2, 124.2, 121.0, 120.2, 112.8 (Ar-C), 55.5 (OCH_3), 44.0 (CH_2). LC-MS (m/z) calculated for $C_{14}H_{12}BrNO_2$: 306.16 found 307.20 $(M+1)^+$.

Synthesis of 2-bromo-4-(3-(3-methoxyphenyl)-1-phenyl-1H-pyrazol-4-yl)pyridine

A solution of (1.16 g, 3.8 mmol) of 2-(2-bromopyridin-4-yl)-1-(3-methoxyphenyl)ethan-1-one in DMF-DMA (5.14 mL, 38.2 mmol) was refluxed for 18 h. The solution was cooled down and concentrated under reduced pressure. The residue was dissolved in 5 mL of anhydrous ethanol. Phenyl hydrazine (0.394 mL, 4 mmol) was added to the ethanolic solution and the mixture was stirred overnight at room temperature. Water (5 mL) was added to the reaction mixture and the organics were extracted with ethyl acetate (3 x 15 mL). The combined organic layer extracts were washed with brine and dried over anhydrous Na_2SO_4. After evaporation of the organic solvent, the residue was purified by column chromatography (silica gel, hexane-ethyl acetate 100:1 v/v) to yield the title compound 2-bromo-4-(3-(3-methoxyphenyl)-1-phenyl-1*H*-pyrazol-4-yl)pyridine (729 mg, 48 %) yellow solid ; mp 96-98 ℃; IR (KBr, Cm^{-1}): 3078, 2964, 1593, 1262; ^{1}H NMR (400 MHz, $CDCl_3$) δ 8.16 (d, *J* = 5.2 Hz, 1H, Ar-H), 7.98 (s, 1H, ar-H), 7.38-7.37 (m, 1H, Ar-H), 7.31-7.23 (m, 5H, Ar-H), 7.00 (dd, *J* = 5.2, *J* = 1.2 Hz, 1H, Ar-H), 6.94-6.91 (m, 1H, Ar-H), 6.77-6.75 (m, 1H, Ar-H), 6.68-6.66 (m, 1H, Ar-H), 3.66 (s, 3H, OCH_3); ^{13}C NMR (100 MHz, $CDCl_3$) δ 159.9, 150.0, 143.6, 142.6, 140.7, 139.3, 130.3, 128.9, 127.9, 125.7, 125.1, 122.5, 120.9, 118.3, 115.5, 115.2 (Ar-C), 55.3 (OCH_3); LC-MS(m/z) calculated for $C_{21}H_{16}BrN_3O$ (m/z):406.05 found: 407.0 $(M + 1)^+$.

Synthesis of N^1-(4-(3-(3-Methoxyphenyl)-1-phenyl-1H-pyrazol-4-yl)pyridin-2-yl)ethane1,2-diamine and N^1-(4-(3-(3-Methoxyphenyl)-1-phenyl-1H-pyrazol-4-yl)pyridin-2-yl)propane-1,3-diamine

A mixture of 2-bromo-4-(3-(3-methoxyphenyl)-1-phenyl-1*H*-pyrazol-4-yl)pyridine (17.09 g, 42.2 mmol) and Copper Iodide (0.95g, 5mmol) in 50 ml of ethylene diamine or 1,3-diaminopropane was heated at 100 degree for 24 h. The reaction mixture was treated with water (150 mL) and ethyl acetate (150 ml). The organic layer was collected and washed with additional water (100 mL) then dried over Na_2SO_4 and evaporated to get the required product as grayish white solid, which was dried and used in the next step without further purification.

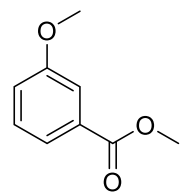
O
O
O

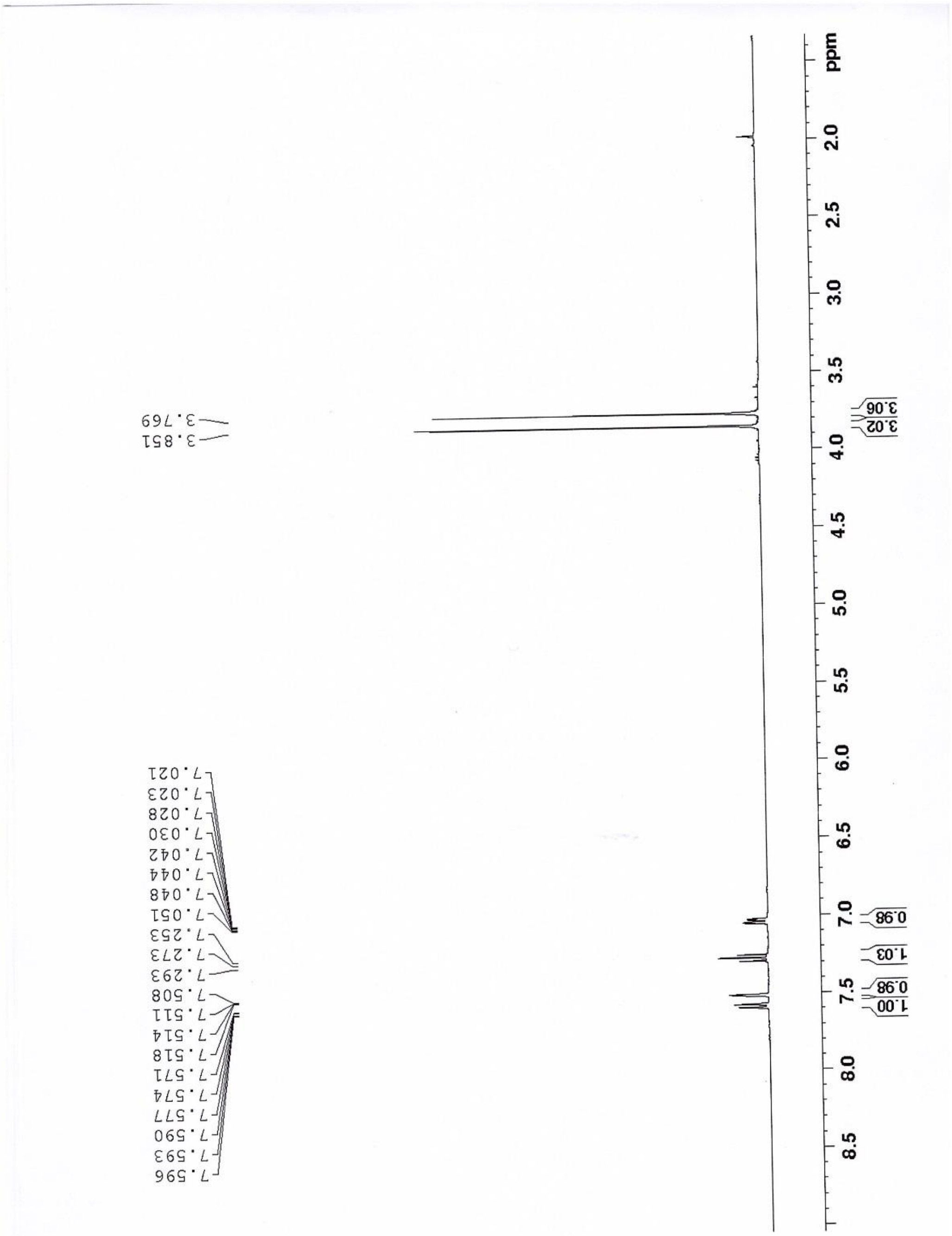
3.769
3.851
7.021
7.023
7.028
7.030
7.042
7.044
7.048
7.051
7.253
7.273
7.293
7.508
7.511
7.514
7.518
7.571
7.574
7.577
7.590
7.593
7.596
3.06
3.02
0.98
1.03
0.98
1.00
ppm
2.0
2.5
3.0
3.5
4.0
4.5
5.0
5.5
6.0
6.5
7.0
7.5
8.0
8.5

6s

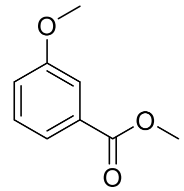
O
O
O

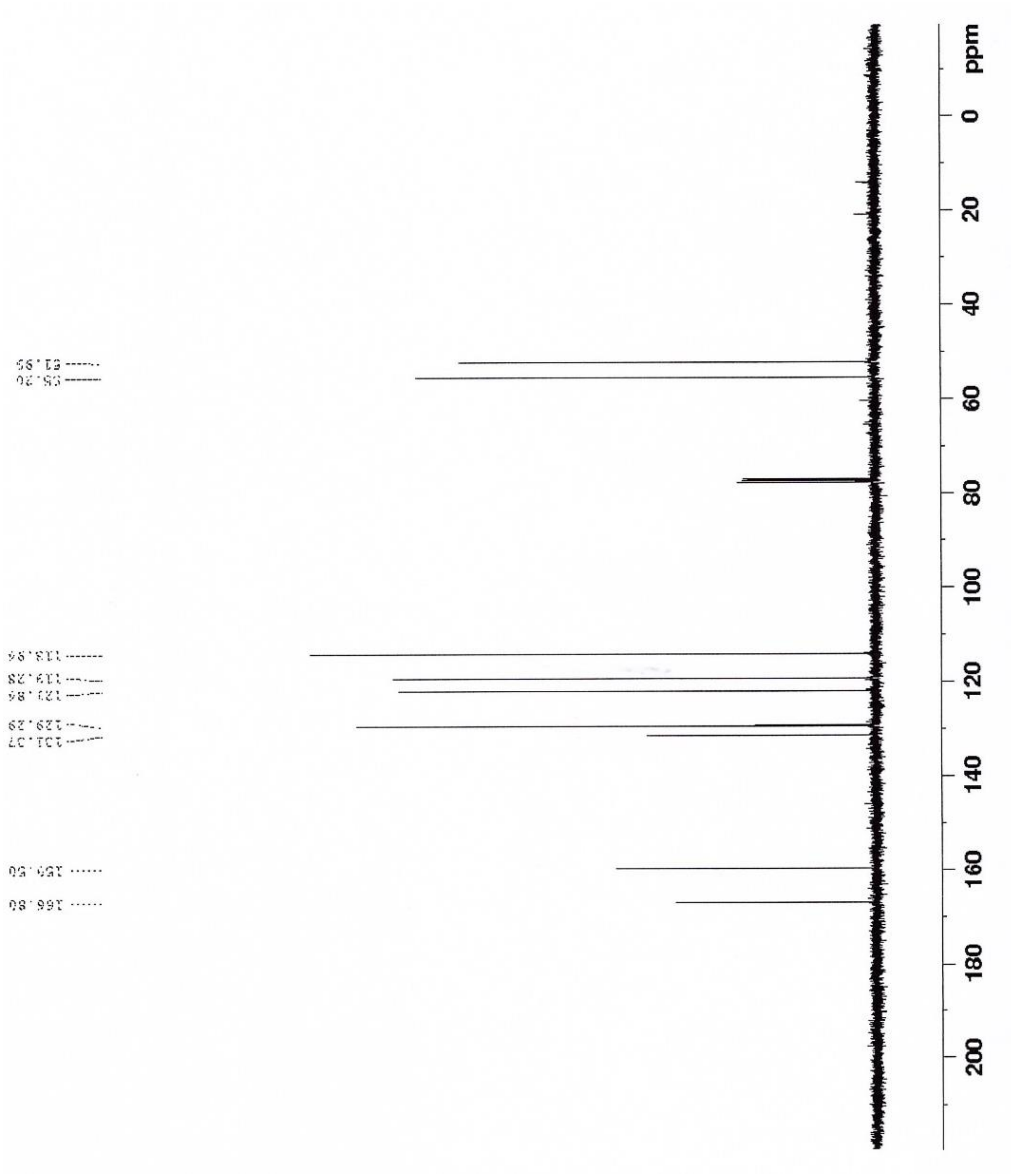
51.95
55.20
113.86
119.28
121.86
129.29
131.07
159.50
166.80
ppm
0
20
40
60
80
100
120
140
160
180
200

7s

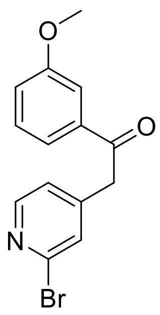
O
O
N
Br

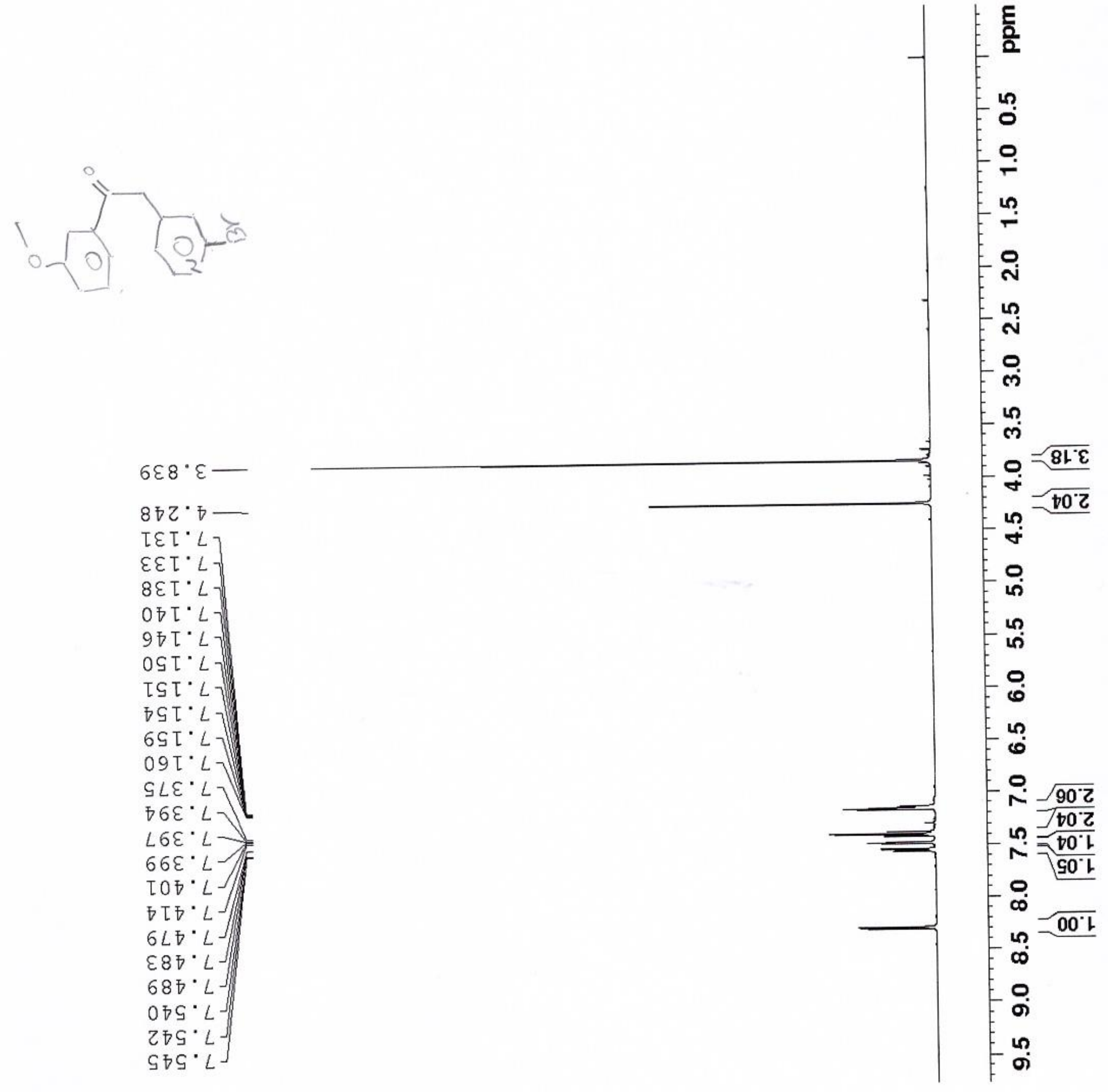
3.839
4.248
7.131
7.133
7.138
7.140
7.146
7.150
7.151
7.154
7.159
7.160
7.375
7.394
7.397
7.399
7.401
7.414
7.479
7.483
7.489
7.540
7.542
7.545
3.18
2.04
2.06
2.04
1.04
1.05
1.00
ppm
0.5
1.0
1.5
2.0
2.5
3.0
3.5
4.0
4.5
5.0
5.5
6.0
6.5
7.0
7.5
8.0
8.5
9.0
9.5

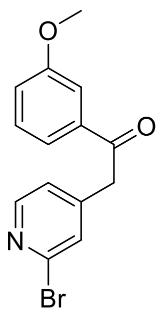
O
O
N
Br

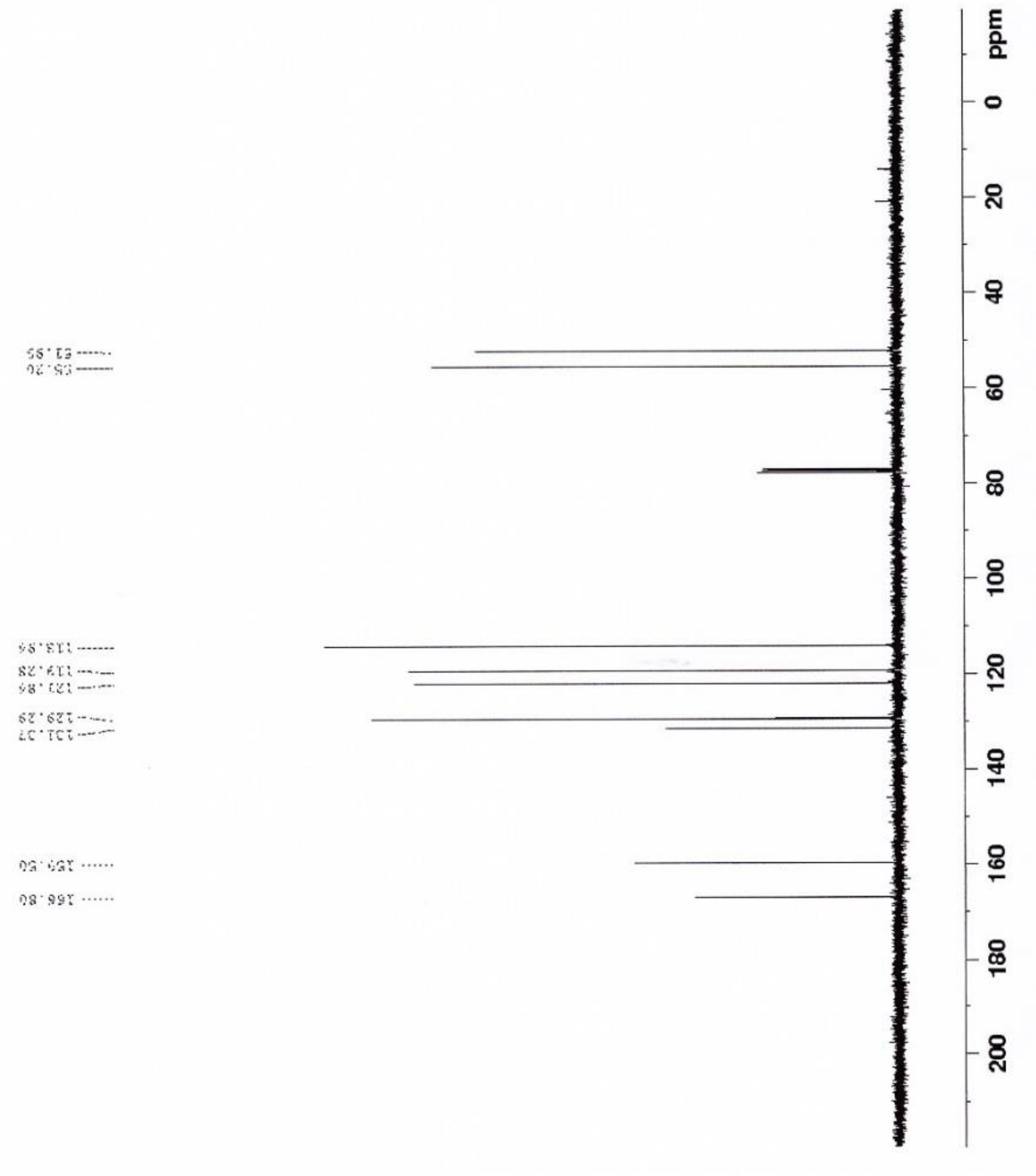

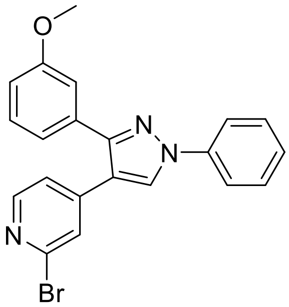

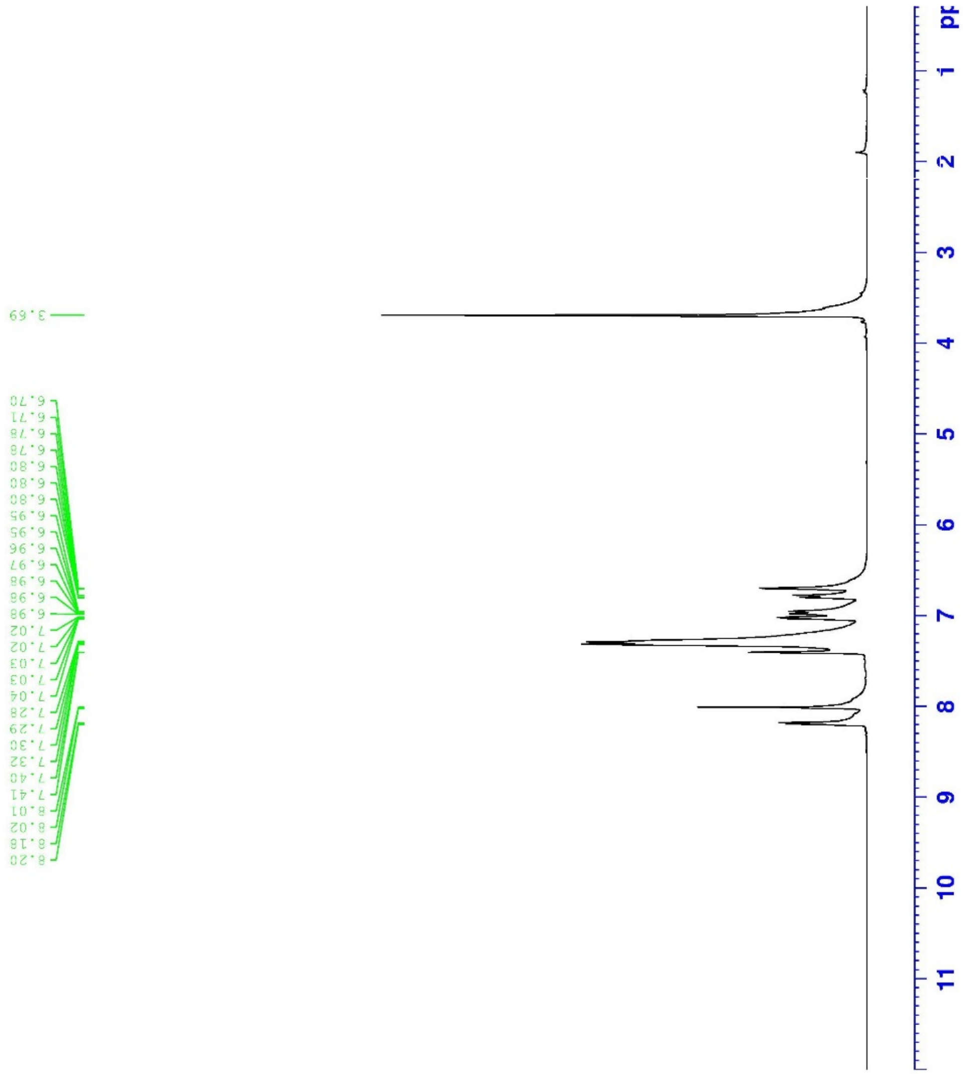

10s

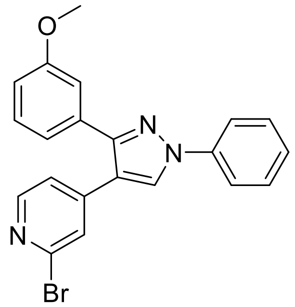
O
N
N
N
Br

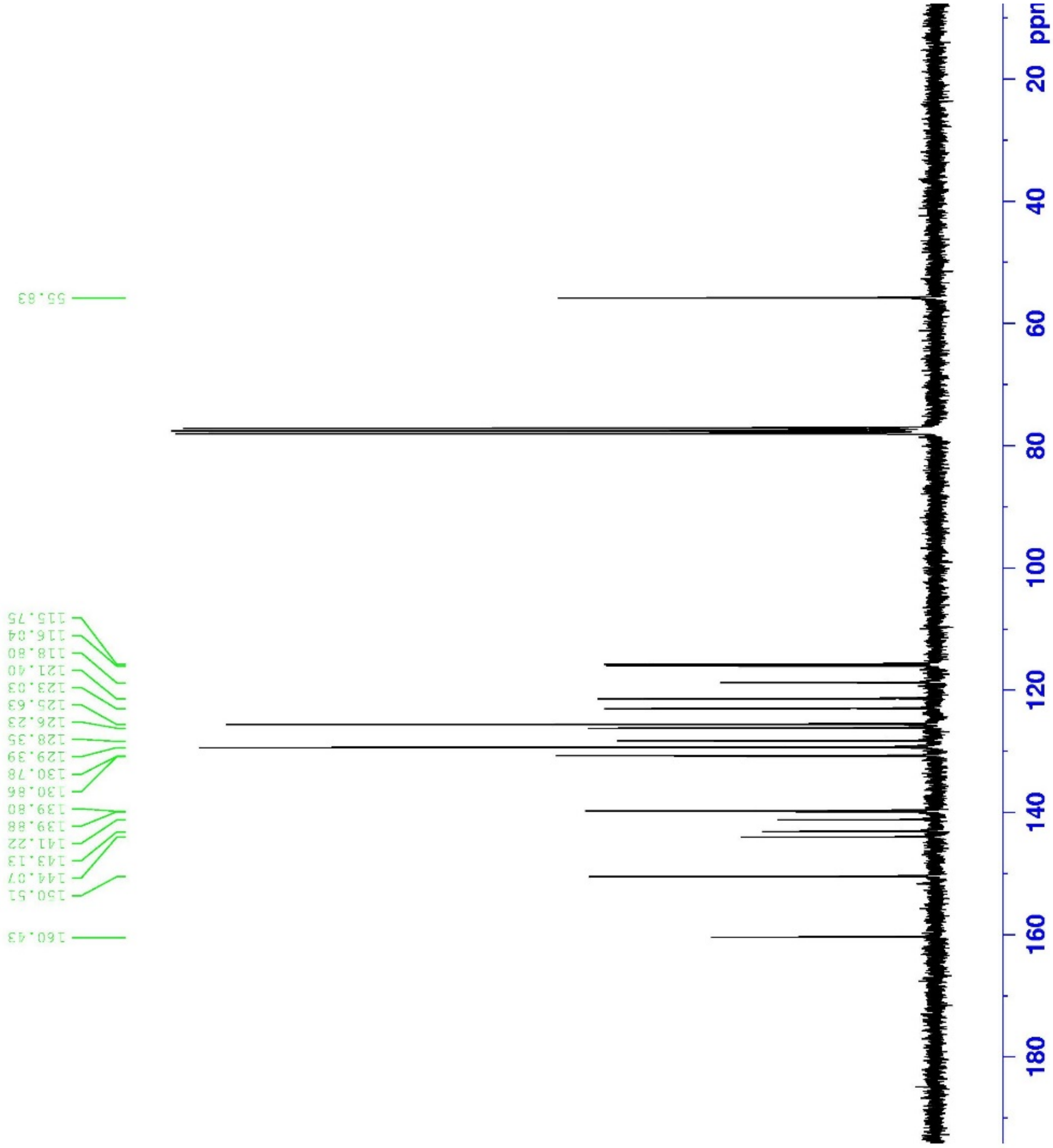
55.83
115.75
116.04
118.80
121.40
123.03
125.63
126.23
128.35
129.39
130.78
130.86
139.80
139.88
141.22
143.13
144.07
150.51
160.43
20
40
60
80
100
120
140
160
180
ppm

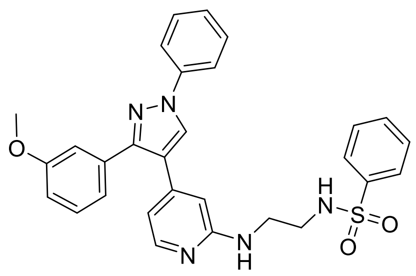

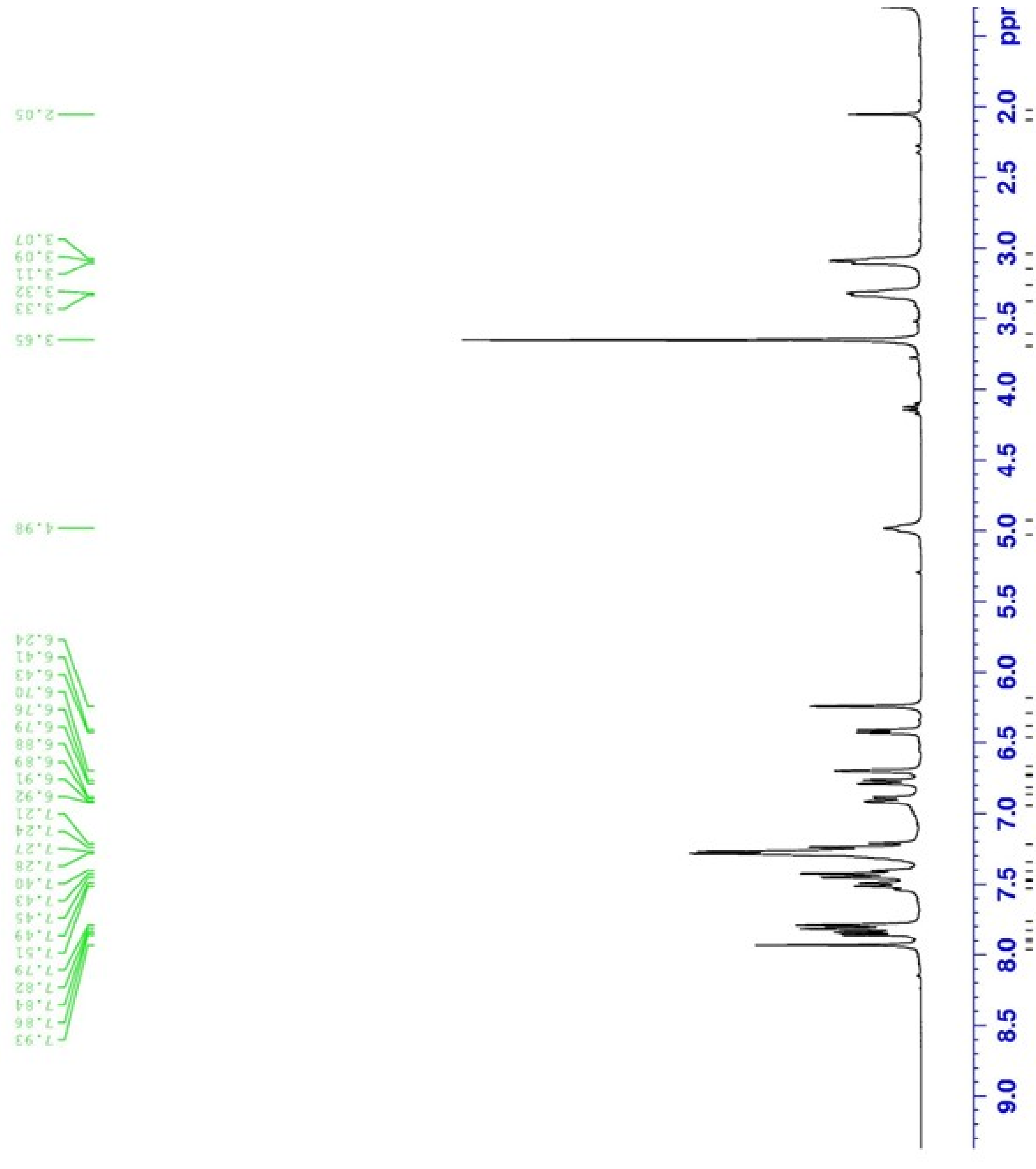

12s

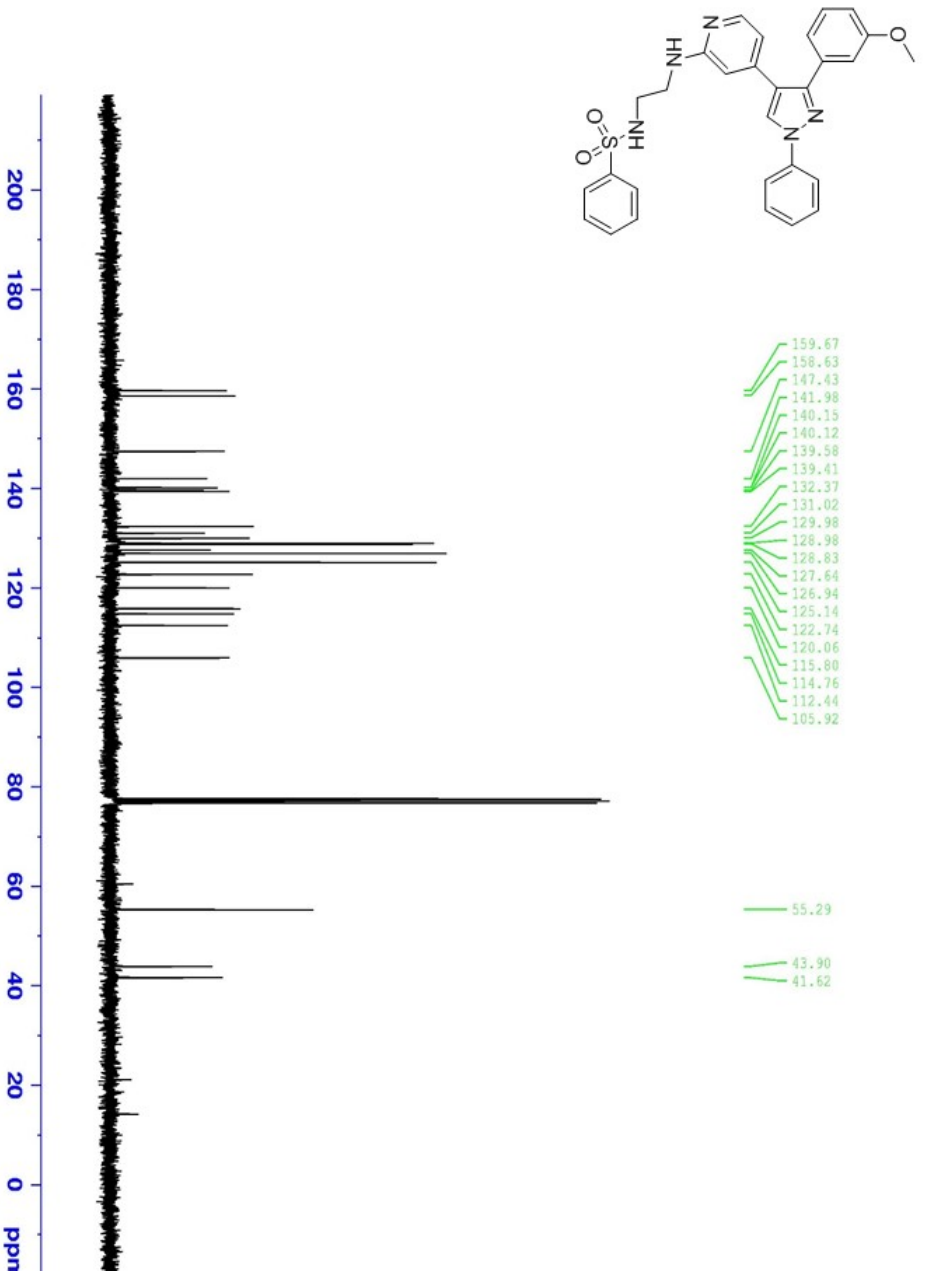
159.67
158.63
147.43
141.98
140.15
140.12
139.58
139.41
132.37
131.02
129.98
128.98
128.83
127.64
126.94
125.14
122.74
120.06
115.80
114.76
112.44
105.92
55.29
43.90
41.62
200
180
160
140
120
100
80
60
40
20
0
ppm

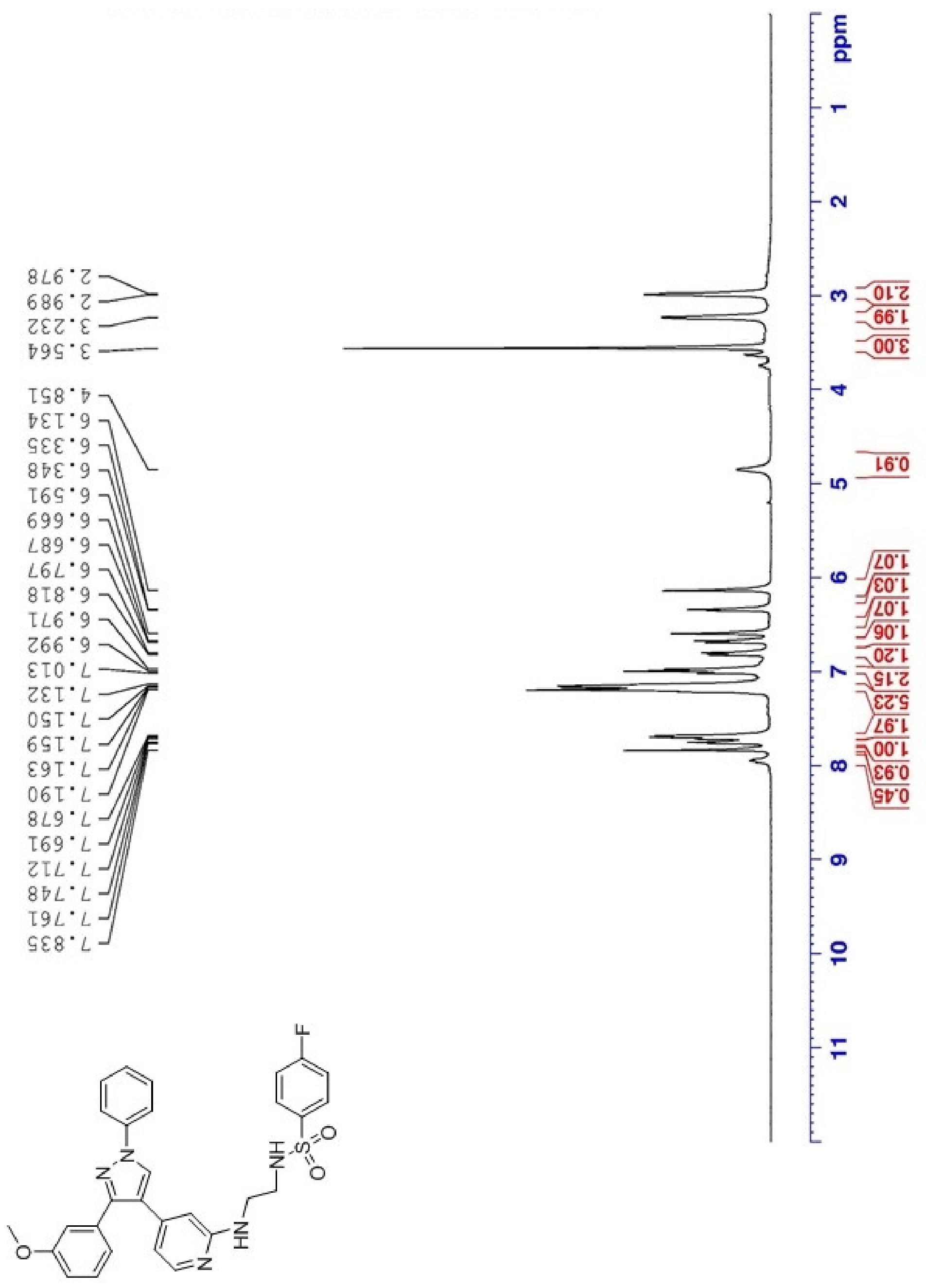
2.978
2.989
3.232
3.564
4.851
6.134
6.335
6.348
6.591
6.669
6.687
6.797
6.818
6.971
6.992
7.013
7.132
7.150
7.159
7.163
7.190
7.678
7.691
7.712
7.748
7.761
7.835
2.10
1.99
3.00
0.91
1.07
1.03
1.07
1.06
1.20
2.15
5.23
1.97
1.00
0.93
0.45
ppm
1
2
3
4
5
6
7
8
9
10
11

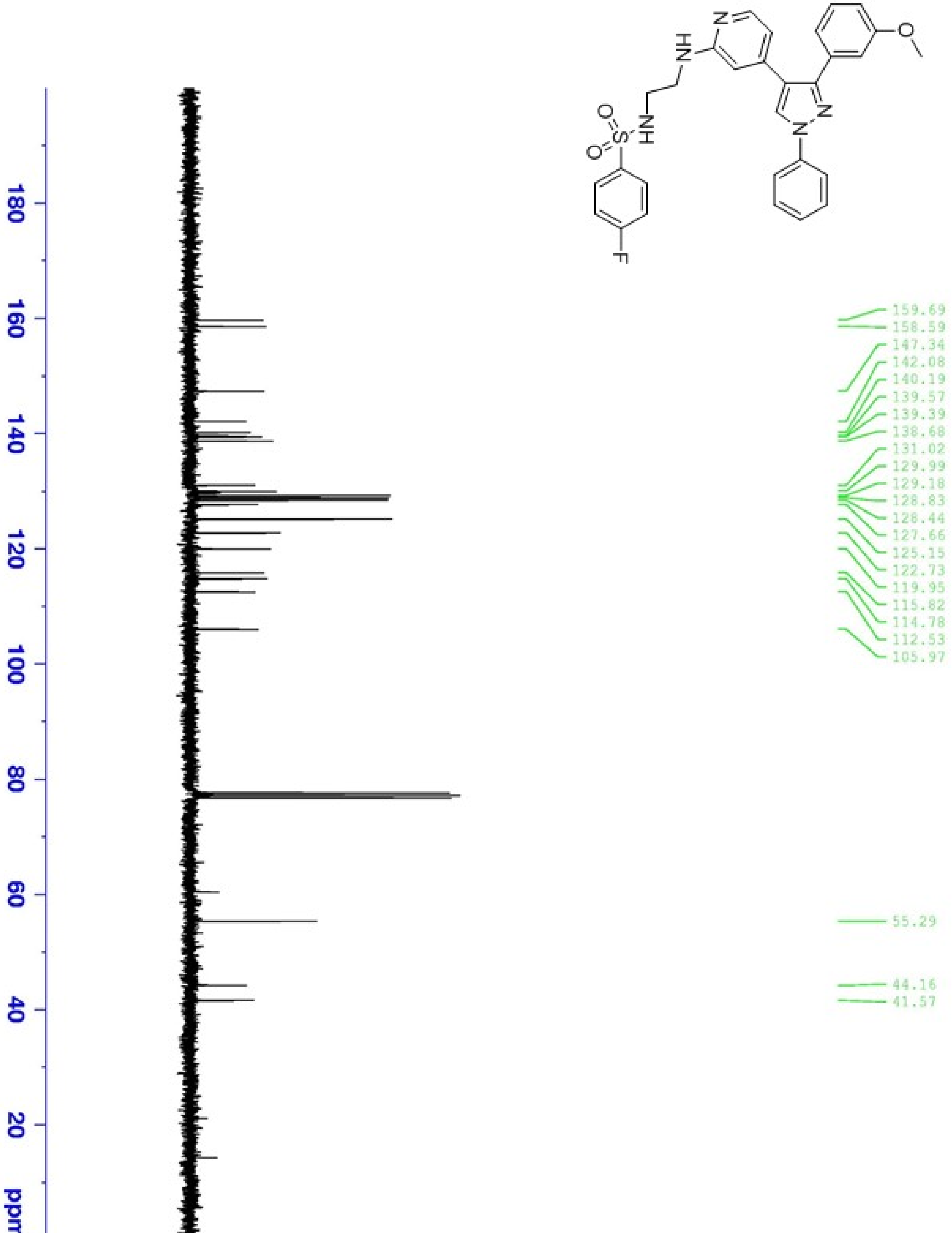

159.69
158.59
147.34
142.08
140.19
139.57
139.39
138.68
131.02
129.99
129.18
128.83
128.44
127.66
125.15
122.73
119.95
115.82
114.78
112.53
105.97
55.29
44.16
41.57
180
160
140
120
100
80
60
40
20
ppm

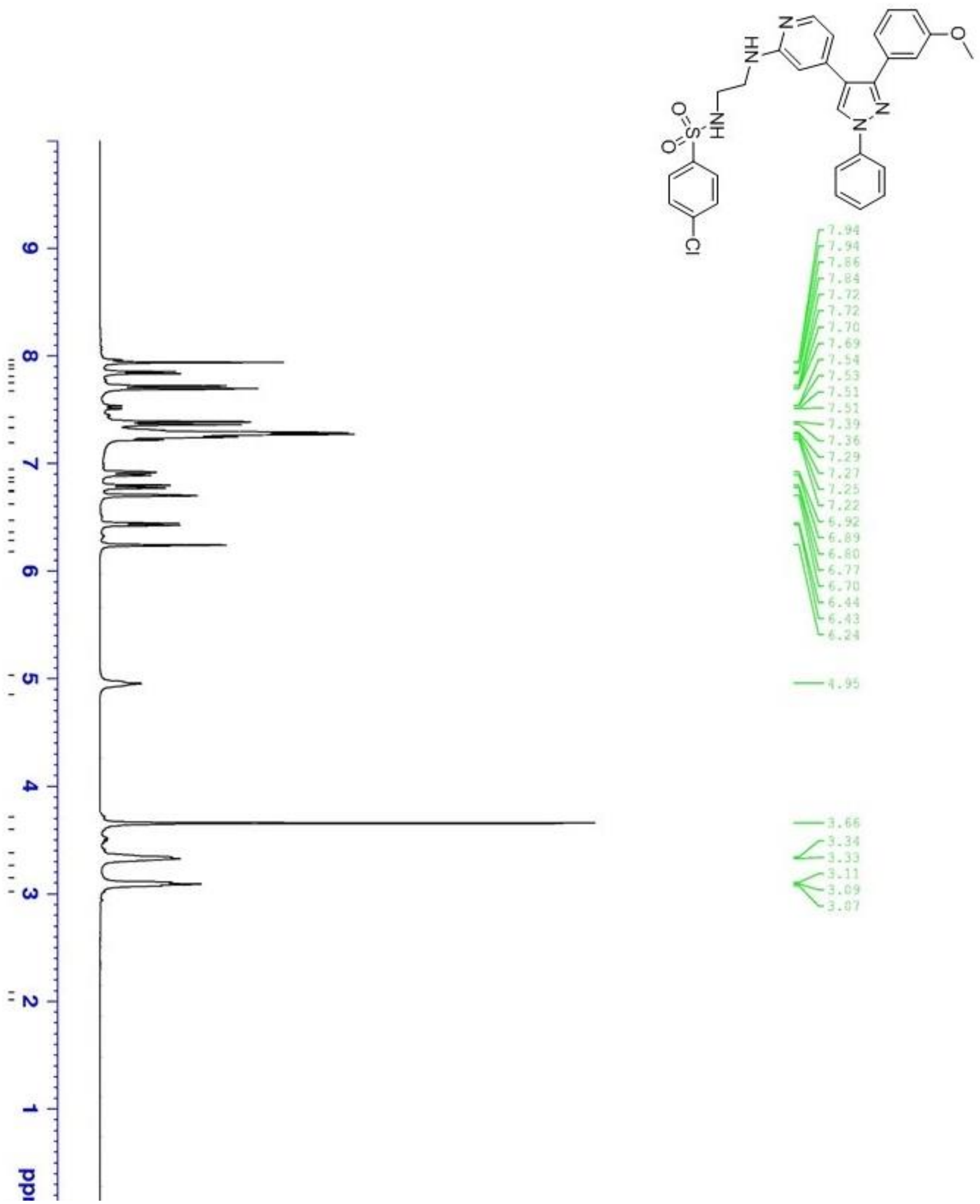

7.94
7.94
7.86
7.84
7.72
7.72
7.70
7.69
7.54
7.53
7.51
7.51
7.39
7.36
7.29
7.27
7.25
7.22
6.92
6.89
6.80
6.77
6.70
6.44
6.43
6.24
4.95
3.66
3.34
3.33
3.11
3.09
3.07
9
8
7
6
5
4
3
2
1
ppm
Cl
O
S
O
NH
HN
N
N
N
O

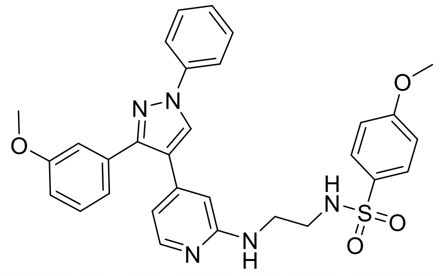

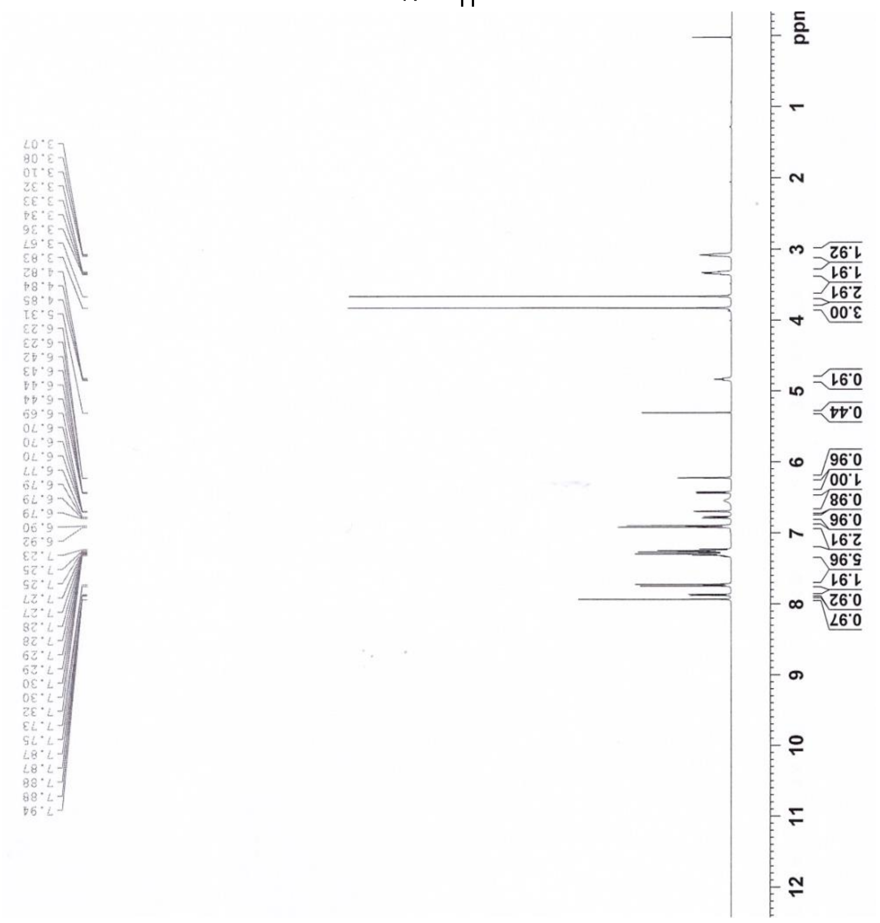

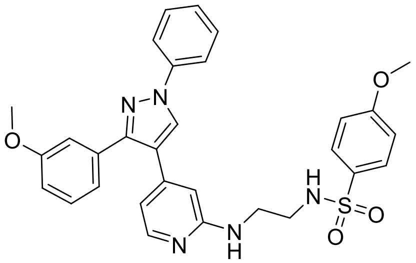

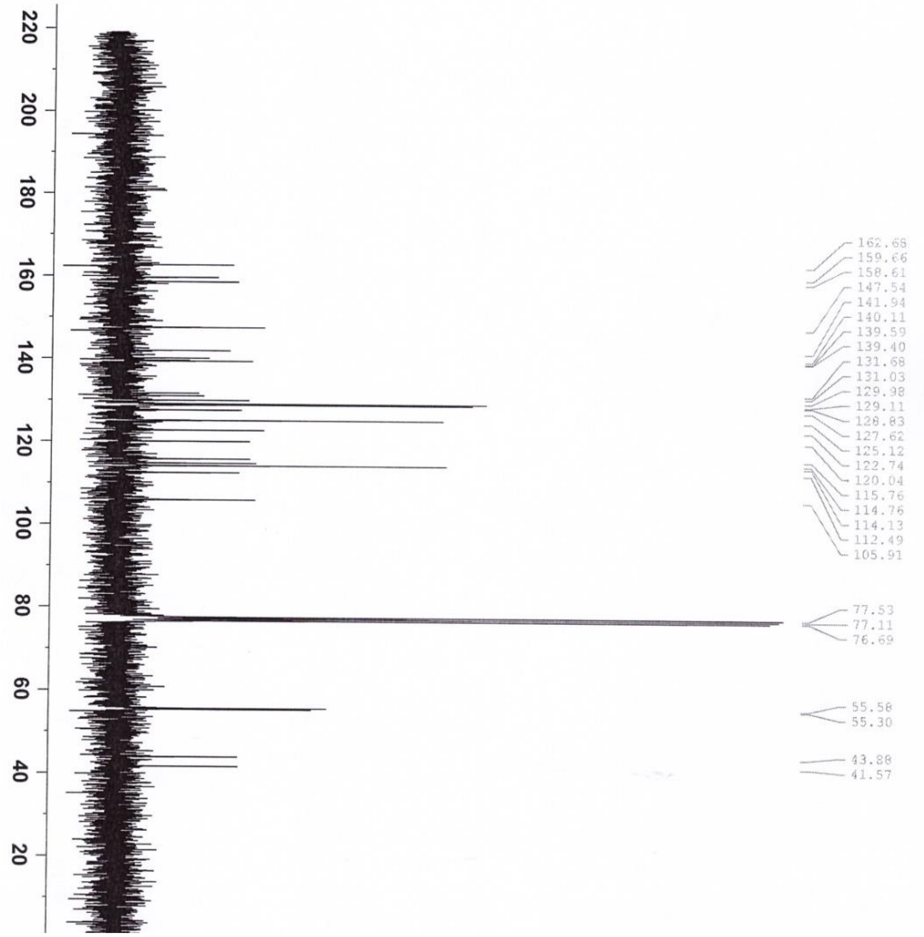
220
200
180
160
140
120
100
80
60
40
20
162.68
159.66
158.61
147.54
141.94
140.11
139.59
139.40
131.68
131.03
129.98
129.11
128.83
127.62
125.12
122.74
120.04
115.76
114.76
114.13
112.49
105.91
77.53
77.11
76.69
55.58
55.30
43.88
41.57

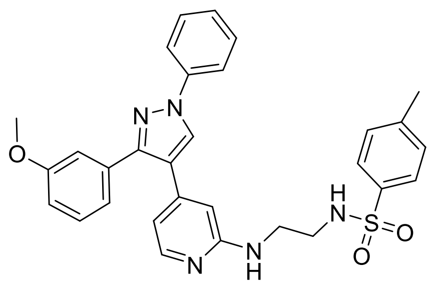
N
N
O
H
N
S
O
O
N
H
N

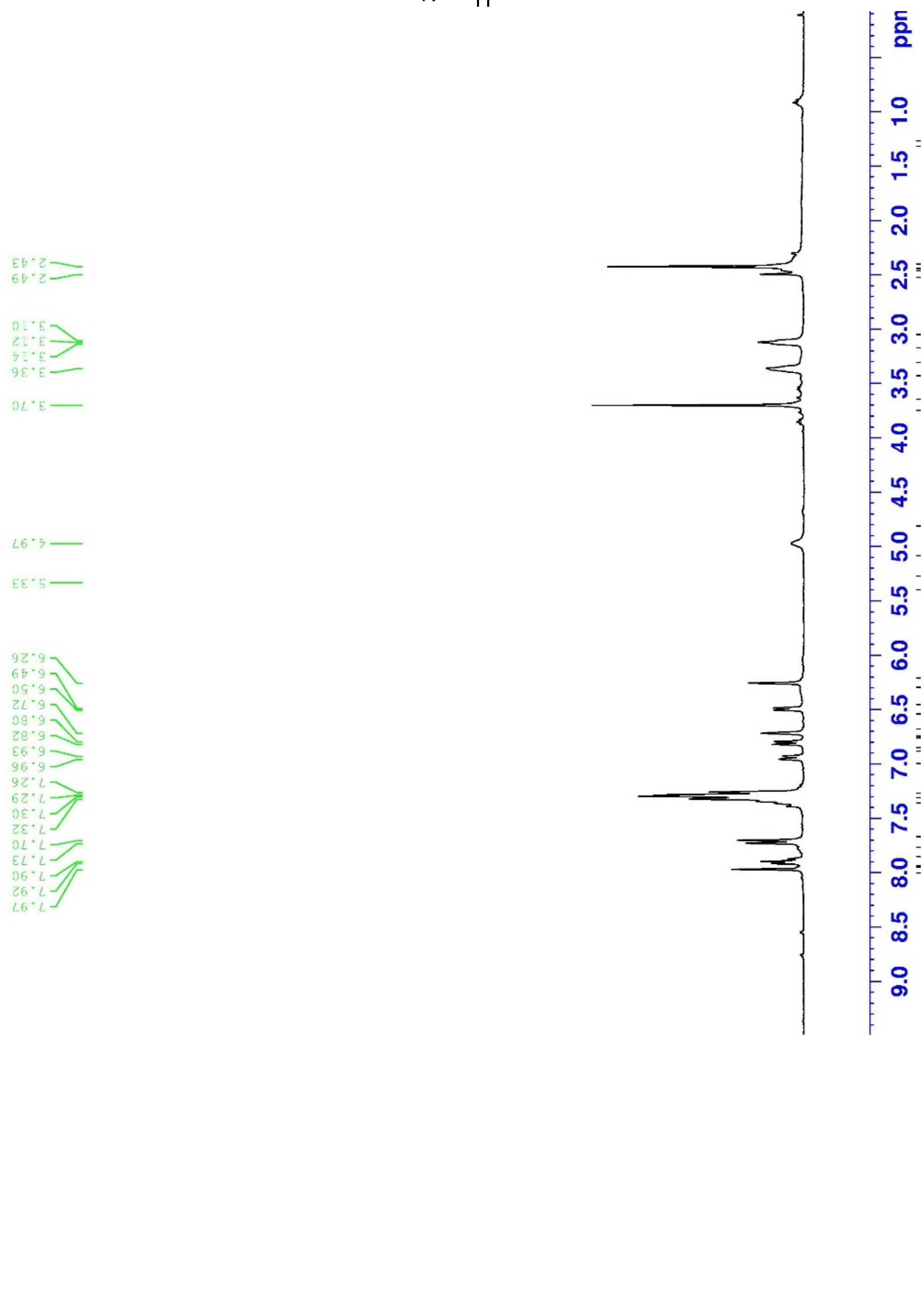
2.43
2.49
3.10
3.12
3.14
3.36
3.70
4.97
5.33
6.26
6.49
6.50
6.72
6.80
6.82
6.93
6.96
7.26
7.29
7.30
7.32
7.70
7.73
7.90
7.92
7.97
ppm
1.0
1.5
2.0
2.5
3.0
3.5
4.0
4.5
5.0
5.5
6.0
6.5
7.0
7.5
8.0
8.5
9.0

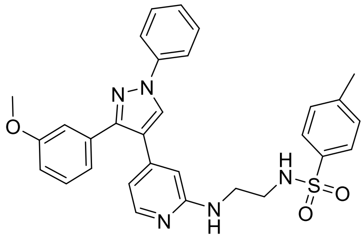

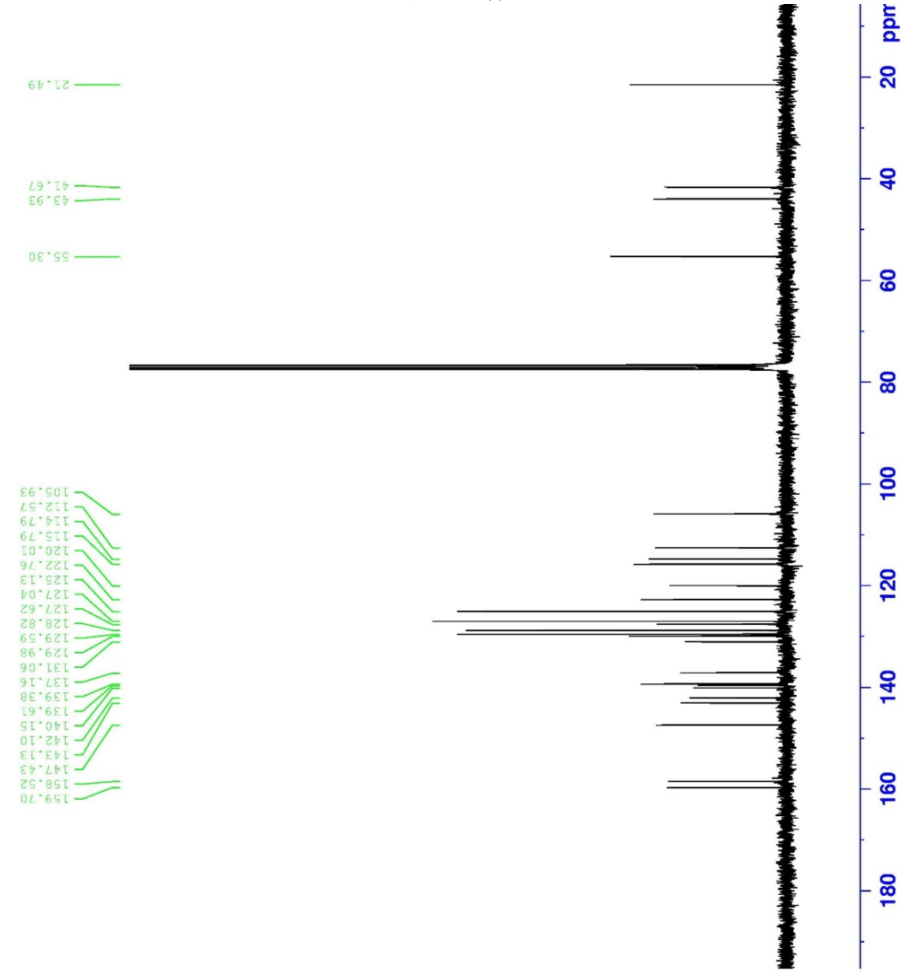
21.49
41.67
43.93
55.30
105.93
112.57
114.79
115.79
120.01
122.76
125.13
127.04
127.62
128.82
129.59
129.98
131.06
137.16
139.38
139.61
140.15
142.10
143.13
147.43
158.52
159.70
ppm
20
40
60
80
100
120
140
160
180

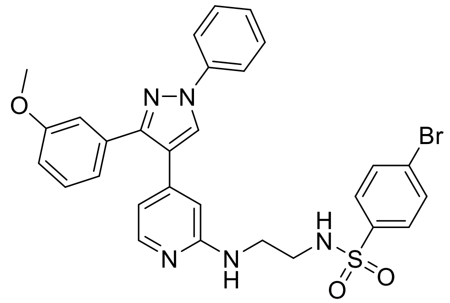

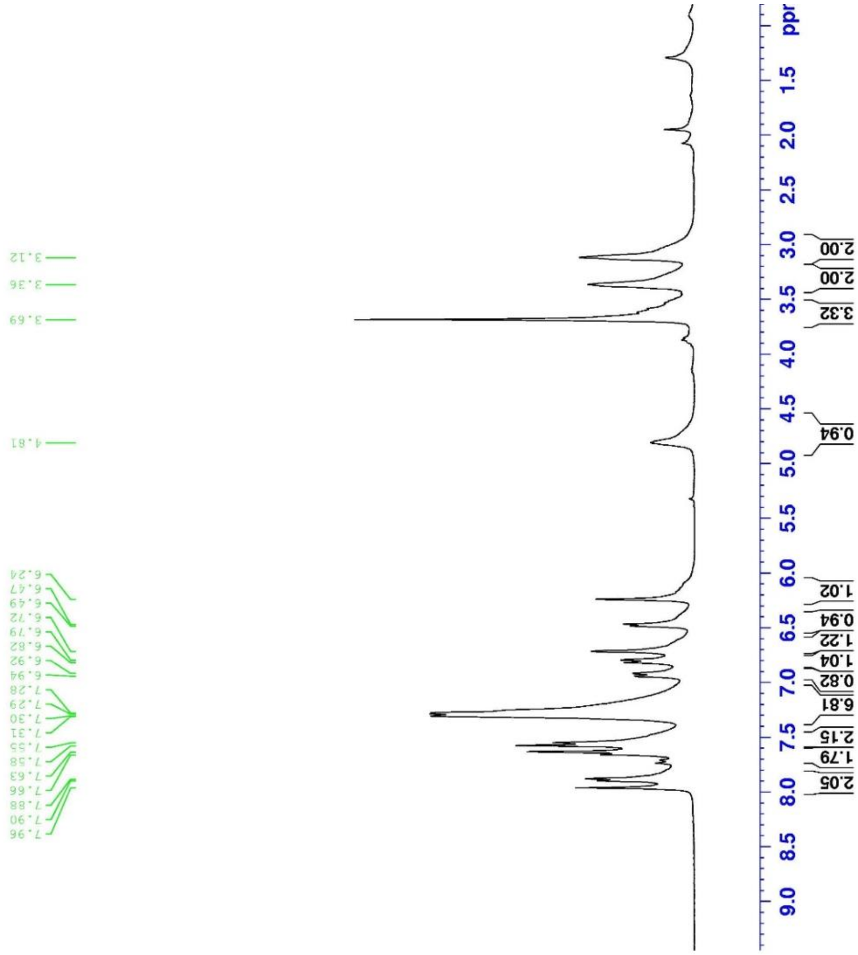

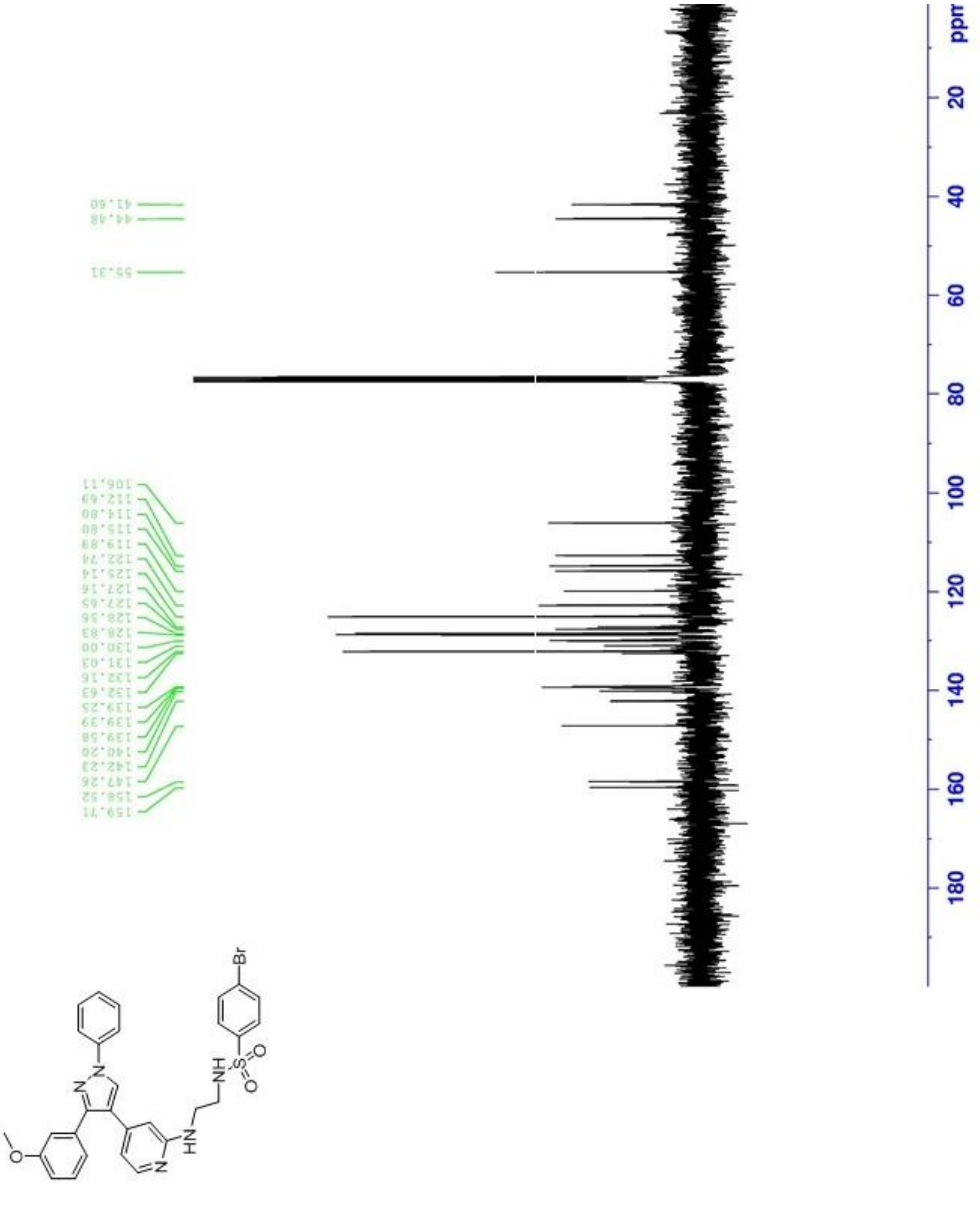
41.60
44.48
55.31
106.11
112.69
114.80
115.80
119.89
122.74
125.14
127.16
127.65
128.56
128.83
130.00
131.03
132.16
132.63
139.25
139.39
139.58
140.20
142.23
147.26
158.52
159.71
ppm
20
40
60
80
100
120
140
160
180
Br
NH
S
O
N
HN

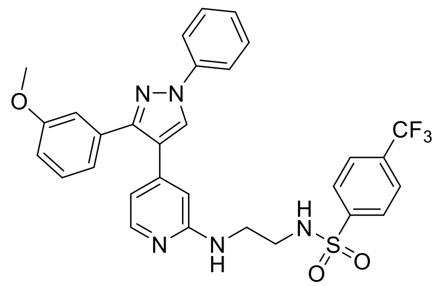

CF3
N
N
O
H
N
S
O
O
N
H
N

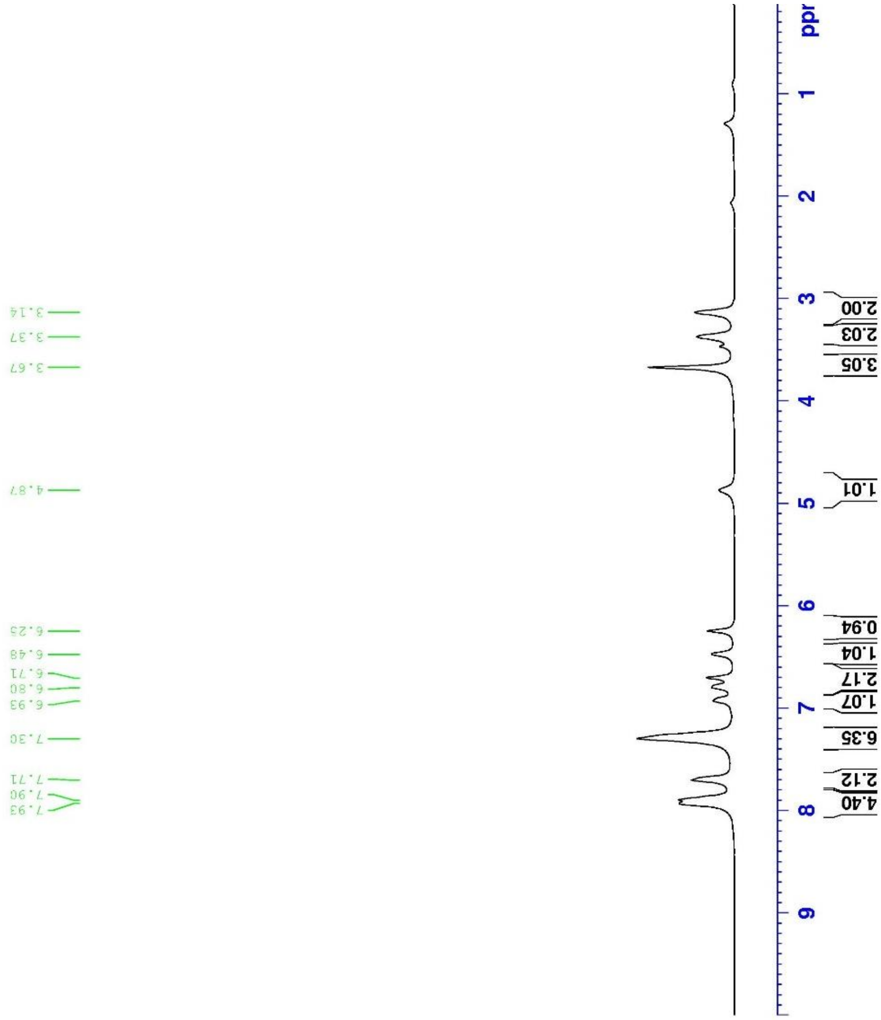

ppm
1
2
3
4
5
6
7
8
9
3.14
3.37
3.67
4.87
6.23
6.48
6.71
6.80
6.93
7.30
7.71
7.90
7.93
2.00
2.03
3.05
1.01
0.94
1.04
2.17
1.07
6.35
2.12
4.40

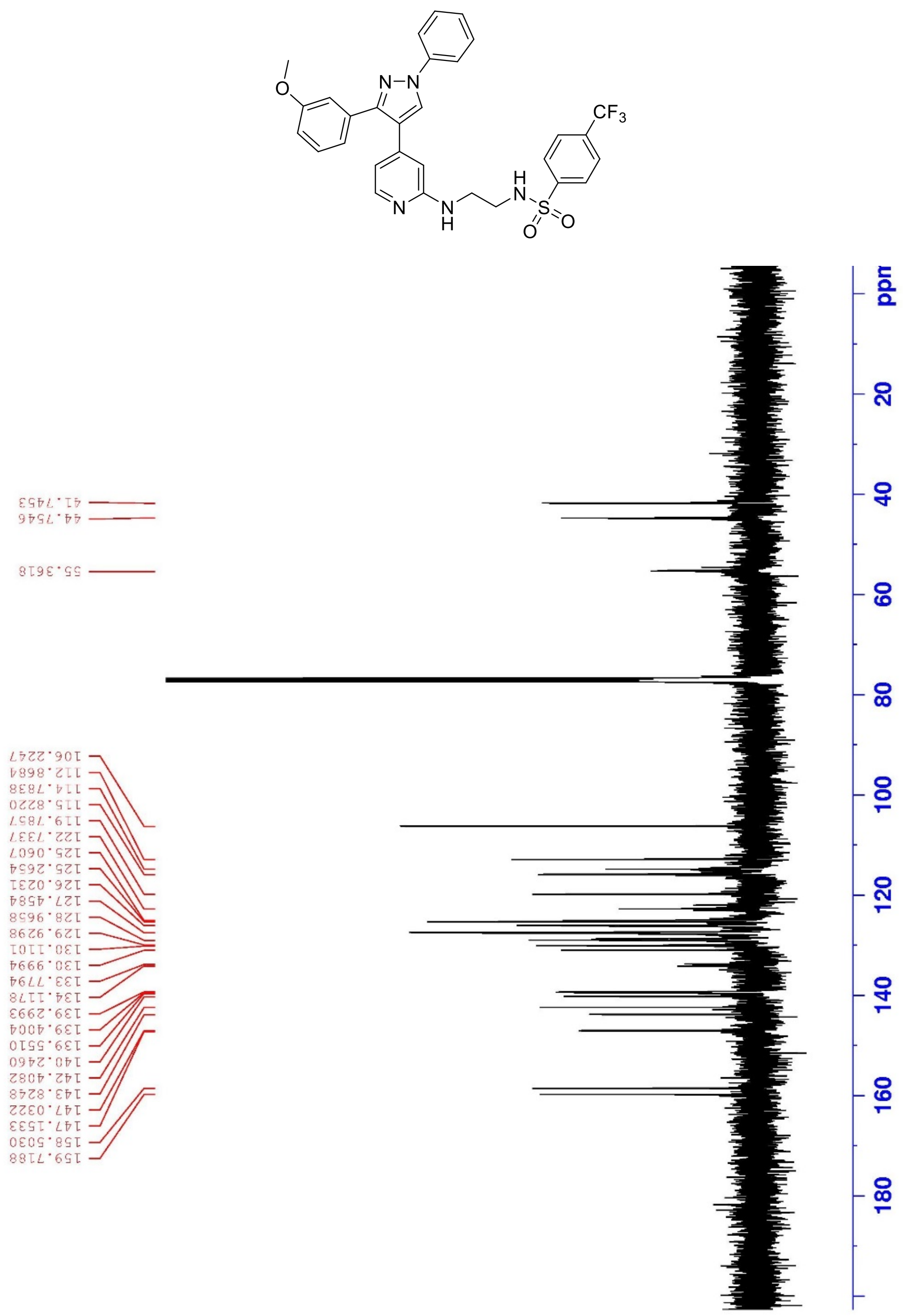

O
N
N
N
N
H
N
H
S
O
O
CF_3
41.7453
44.7546
55.3618
106.2247
112.8684
114.7838
115.8220
119.7857
122.7337
125.0607
125.2654
126.0231
127.4584
128.9658
129.9298
130.1101
130.9994
133.7794
134.1178
139.2993
139.4004
139.5510
140.2460
142.4082
143.8248
147.0322
147.1533
158.5030
159.7188
ppm
20
40
60
80
100
120
140
160
180

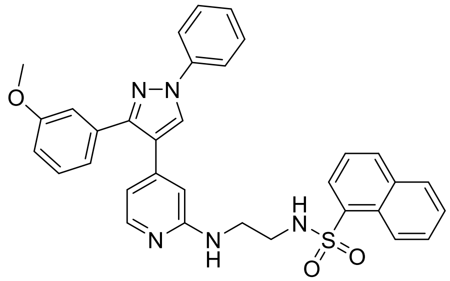

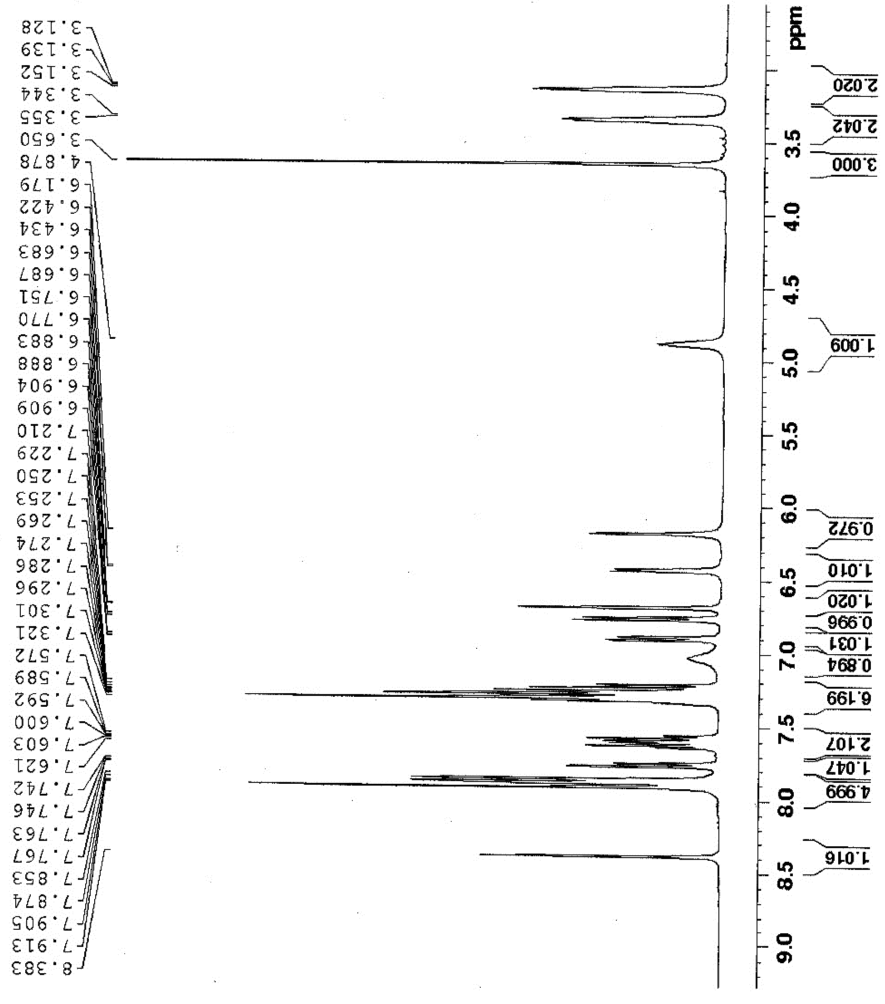

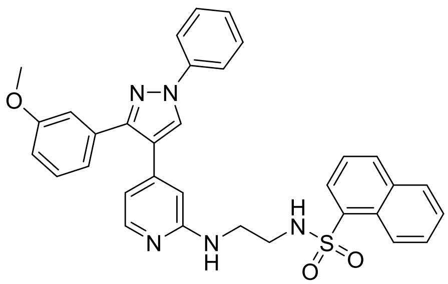

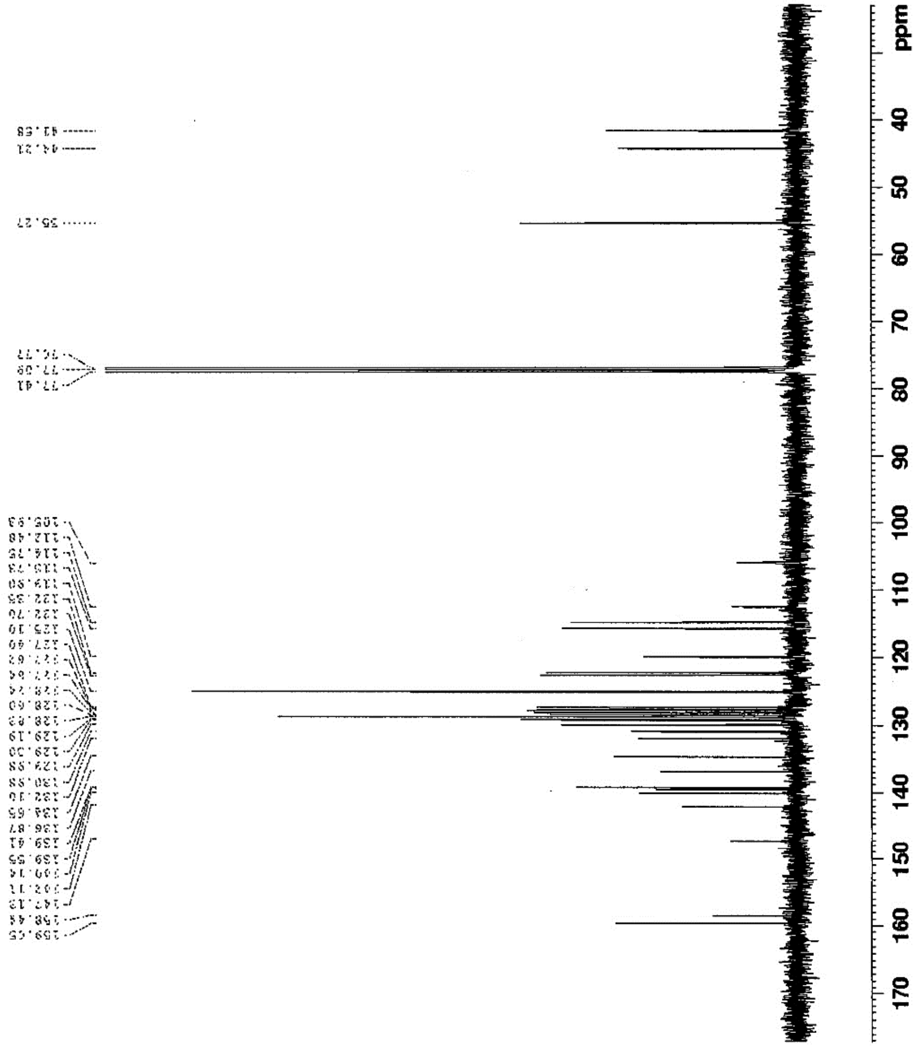

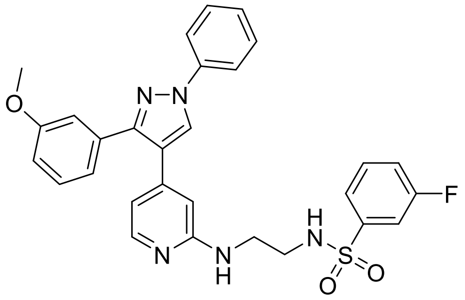

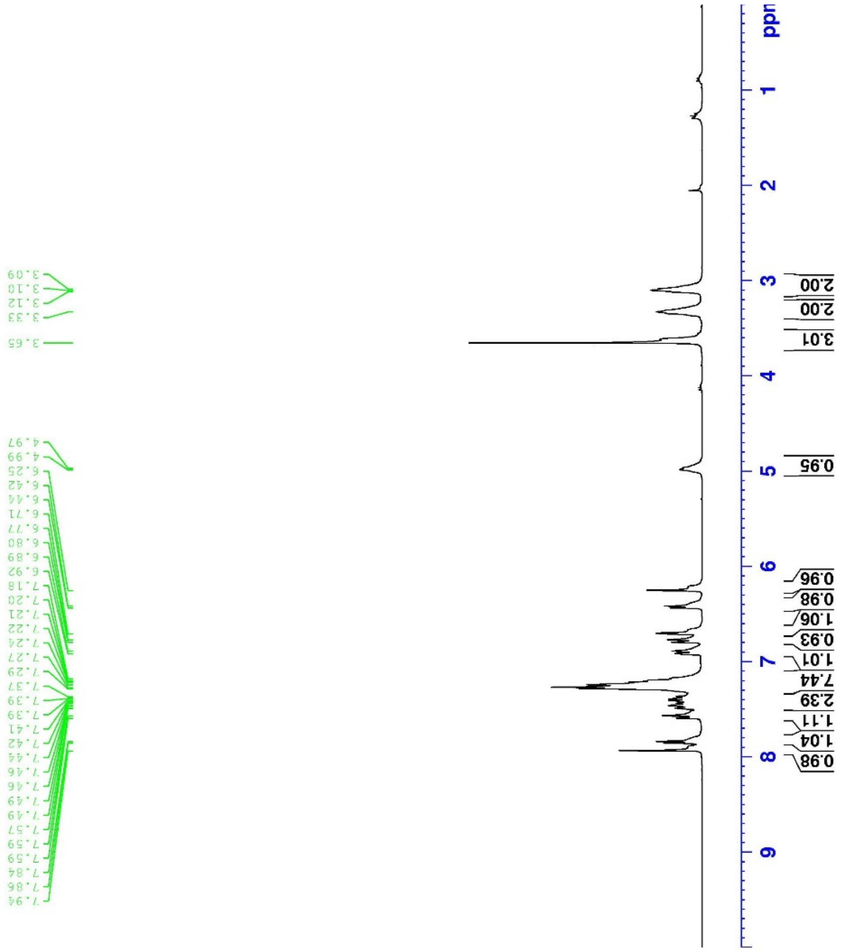

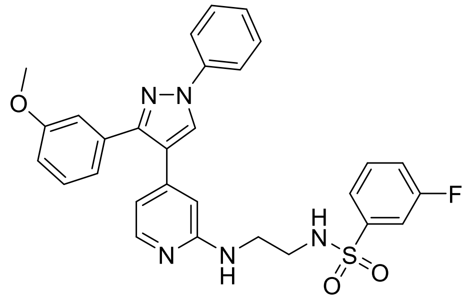
O
N
N
N
H
N
H
N
S
O
O
F

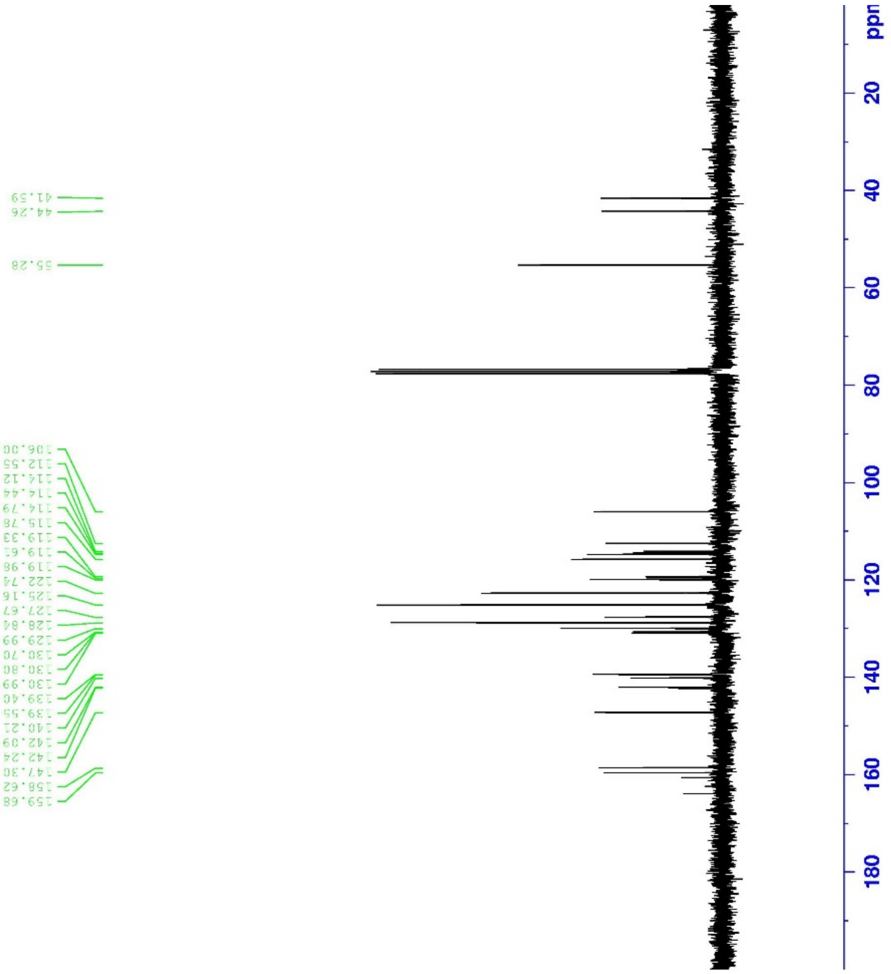
41.59
44.26
55.28
106.00
112.55
114.12
114.44
114.79
115.78
119.33
119.61
119.98
122.74
125.16
127.67
128.84
129.99
130.70
130.80
130.99
139.40
139.55
140.21
142.09
142.24
147.30
158.62
159.68
ppm
20
40
60
80
100
120
140
160
180

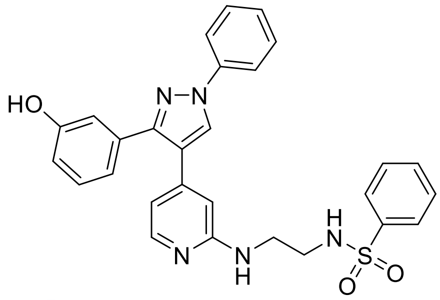

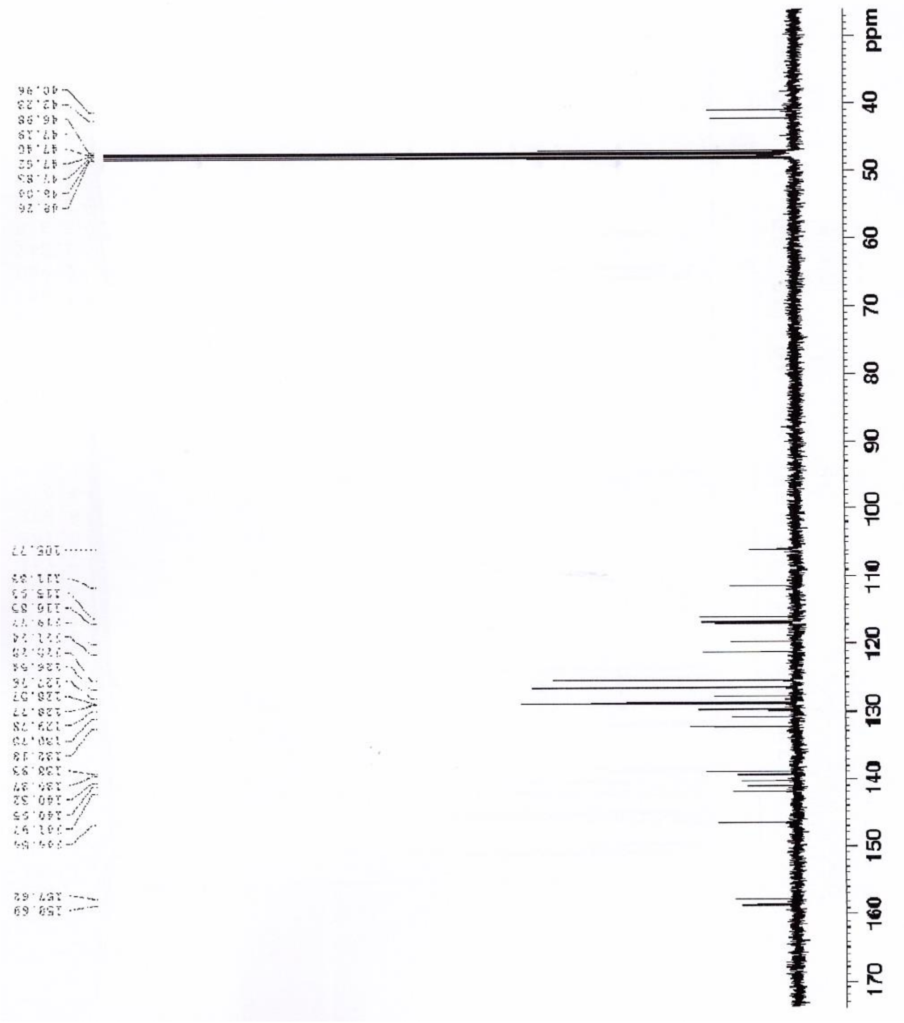

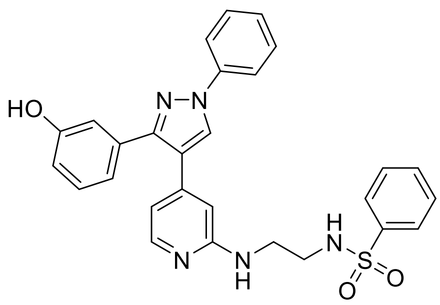
HO
N
N
N
H
N
H
N
S
O
O

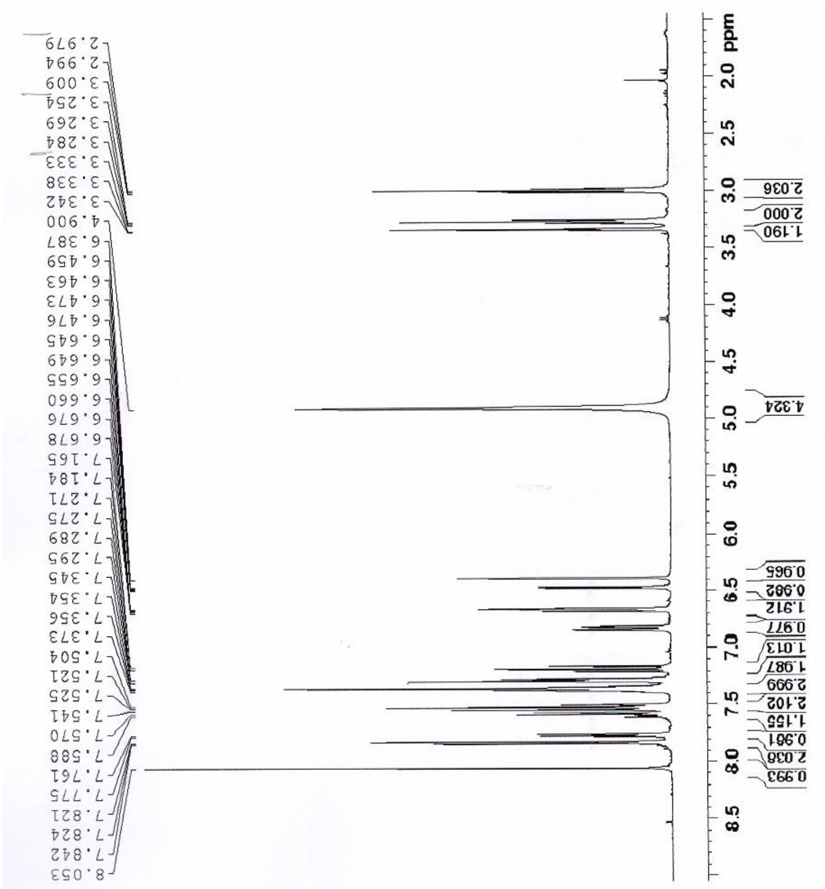
2.979
2.994
3.009
3.254
3.269
3.284
3.333
3.338
3.342
4.900
6.387
6.459
6.463
6.473
6.476
6.645
6.649
6.655
6.660
6.676
6.678
7.165
7.184
7.271
7.275
7.289
7.295
7.345
7.354
7.356
7.373
7.504
7.521
7.525
7.541
7.570
7.588
7.761
7.775
7.821
7.824
7.842
8.053
2.036
2.000
1.190
4.324
0.965
0.982
1.912
0.977
1.013
1.087
2.999
2.102
1.155
0.961
2.038
0.993
ppm
2.0
2.5
3.0
3.5
4.0
4.5
5.0
5.5
6.0
6.5
7.0
7.5
8.0
8.5

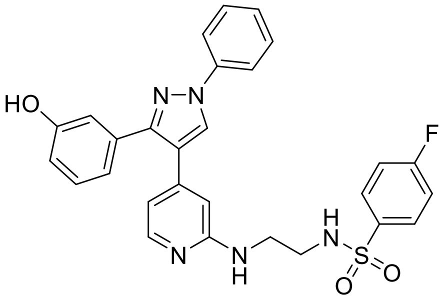

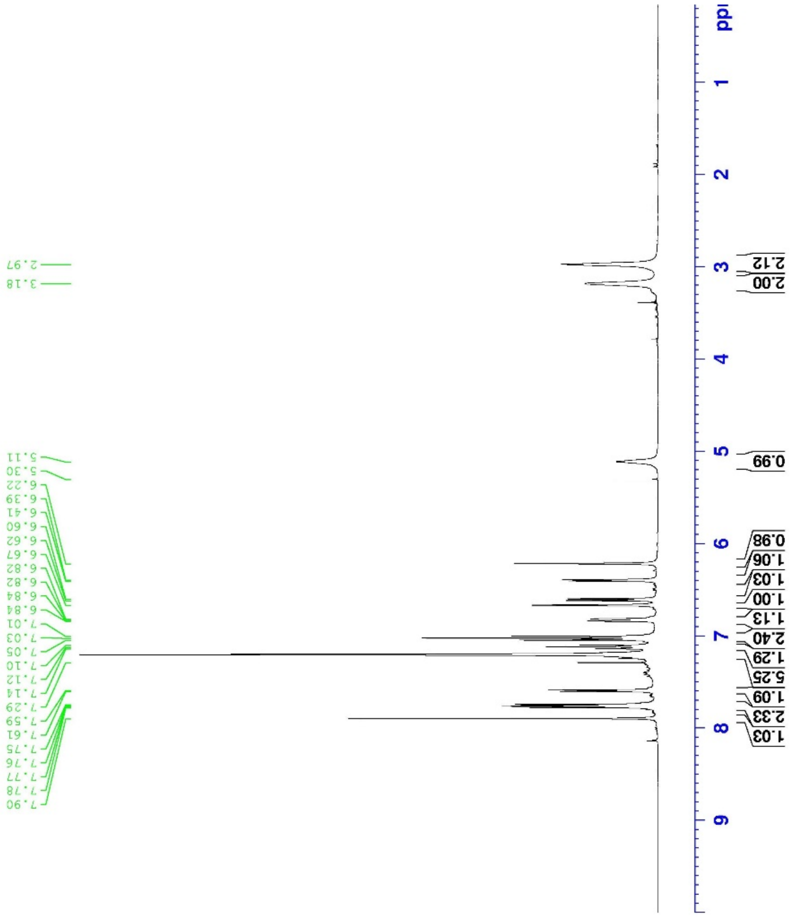

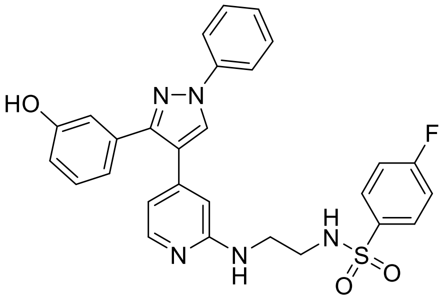
HO
N
N
F
N
H
N
S
O
O
N
H

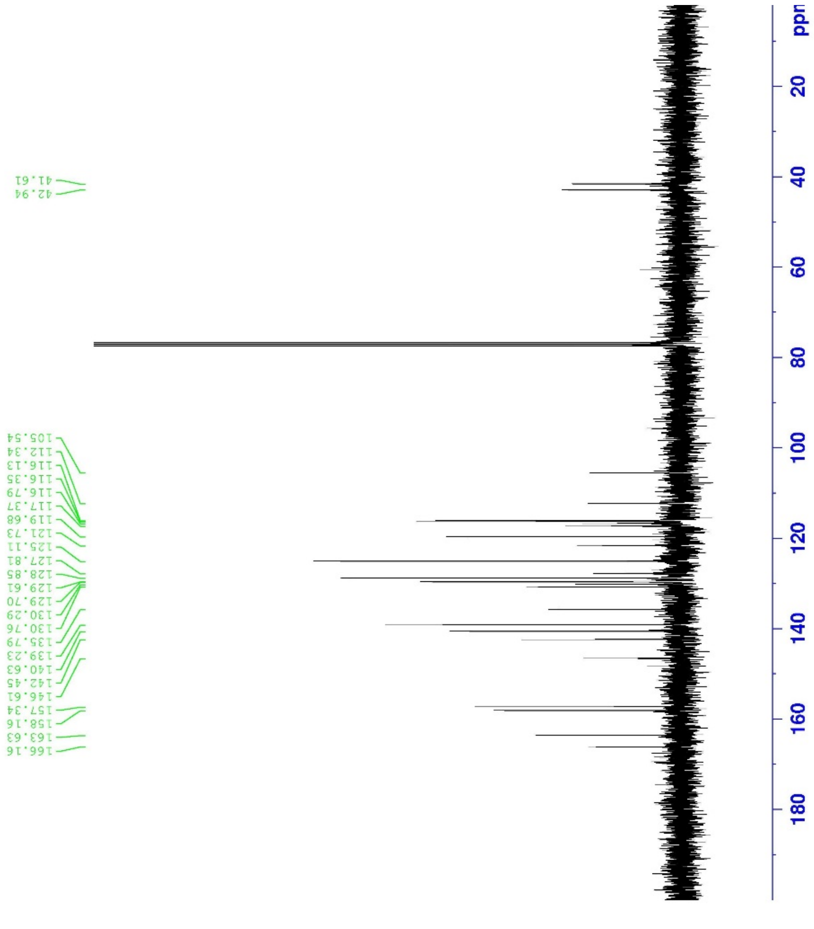
ppm
20
40
60
80
100
120
140
160
180
41.61
42.94
105.54
112.34
116.13
116.35
116.79
117.37
119.68
121.73
125.11
127.81
128.85
129.61
129.70
130.29
130.76
135.79
139.23
140.63
142.45
146.61
157.34
158.16
163.63
166.16

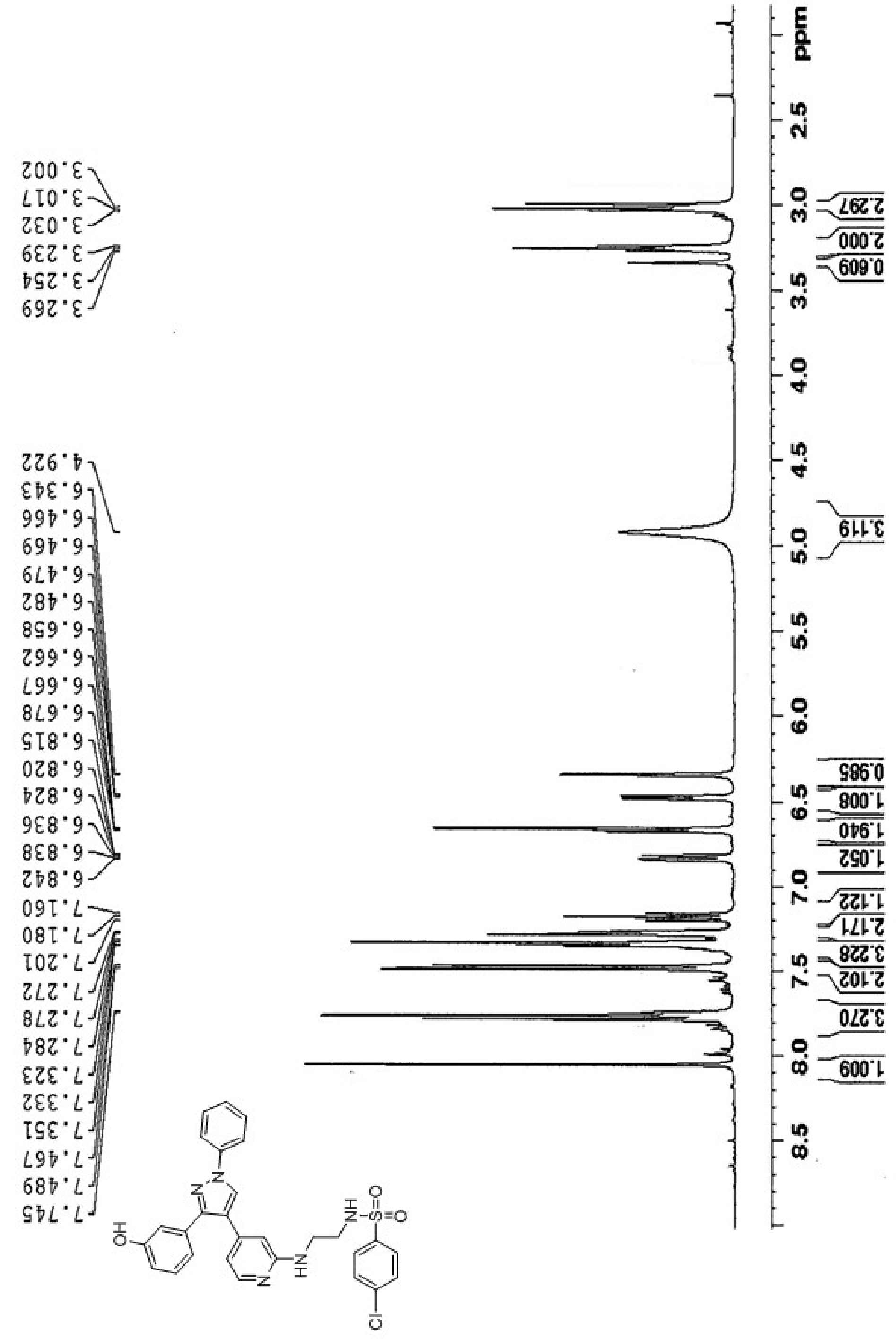
3.002
3.017
3.032
3.239
3.254
3.269
4.922
6.343
6.466
6.469
6.479
6.482
6.658
6.662
6.667
6.678
6.815
6.820
6.824
6.836
6.838
6.842
7.160
7.180
7.201
7.272
7.278
7.284
7.323
7.332
7.351
7.467
7.489
7.745
2.297
2.000
0.609
3.119
0.985
1.008
1.940
1.052
1.122
2.171
3.228
2.102
3.270
1.009
ppm
2.5
3.0
3.5
4.0
4.5
5.0
5.5
6.0
6.5
7.0
7.5
8.0
8.5
OH
N
N
N
HN
NH
S
O
O
Cl

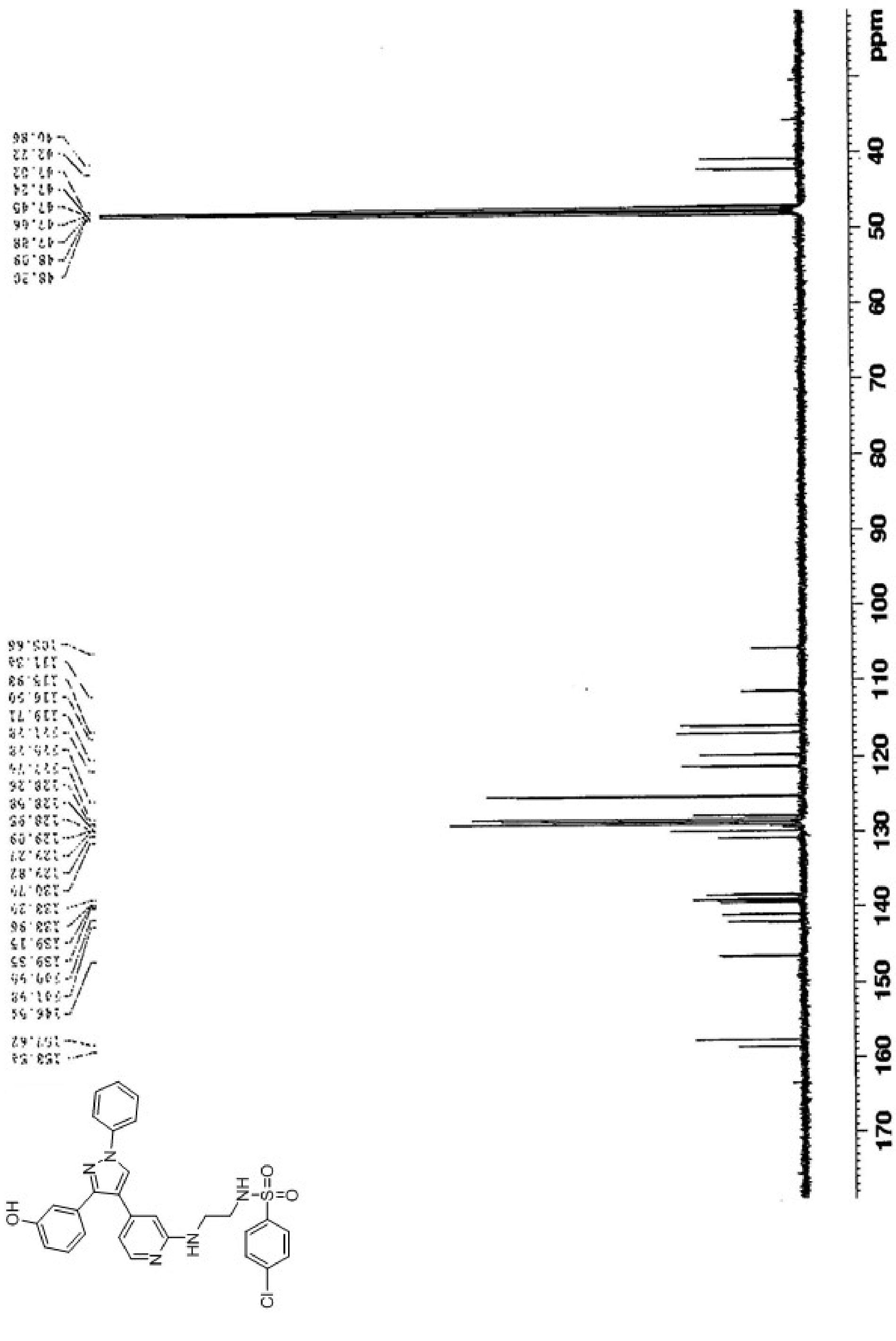
ppm
40
50
60
70
80
90
100
110
120
130
140
150
160
170

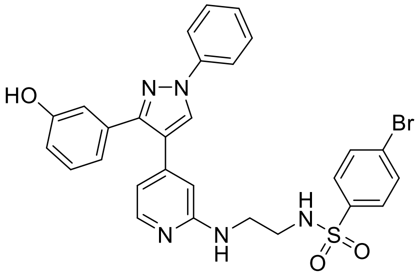
HO
N
N
Br
N
H
N
H
N
S
O
O

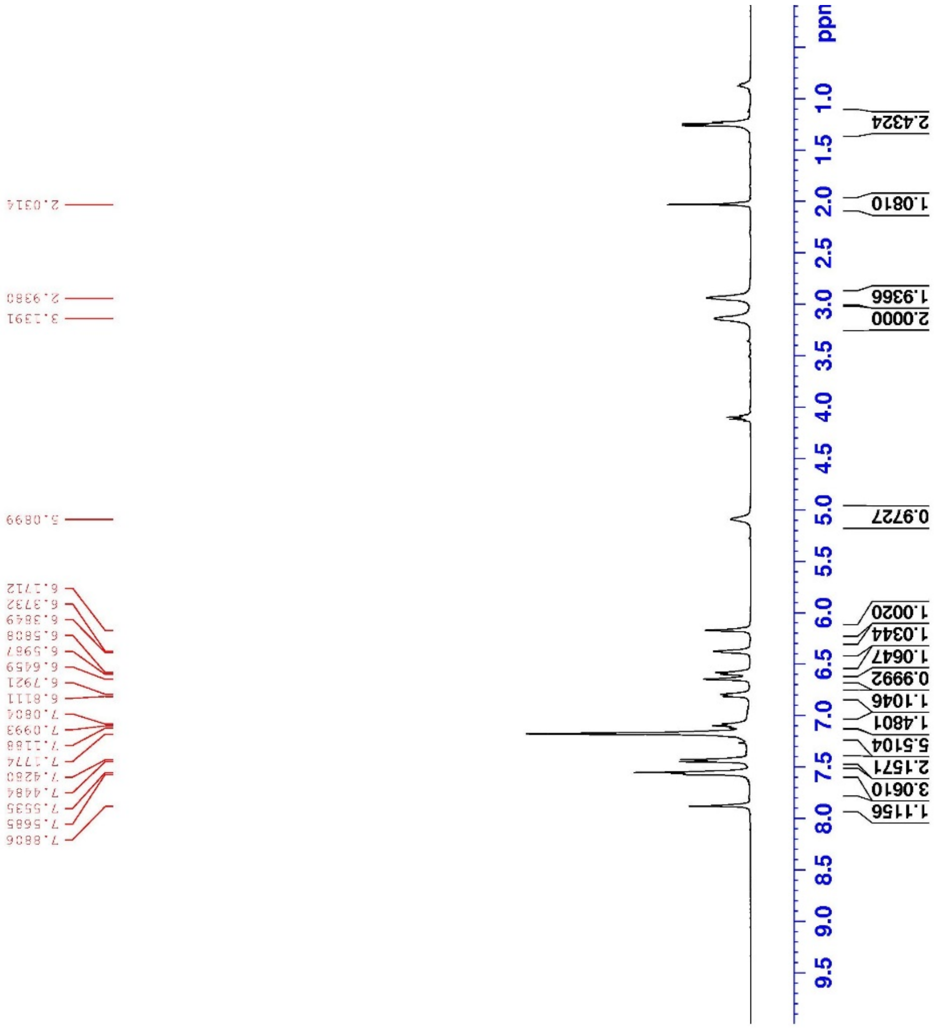

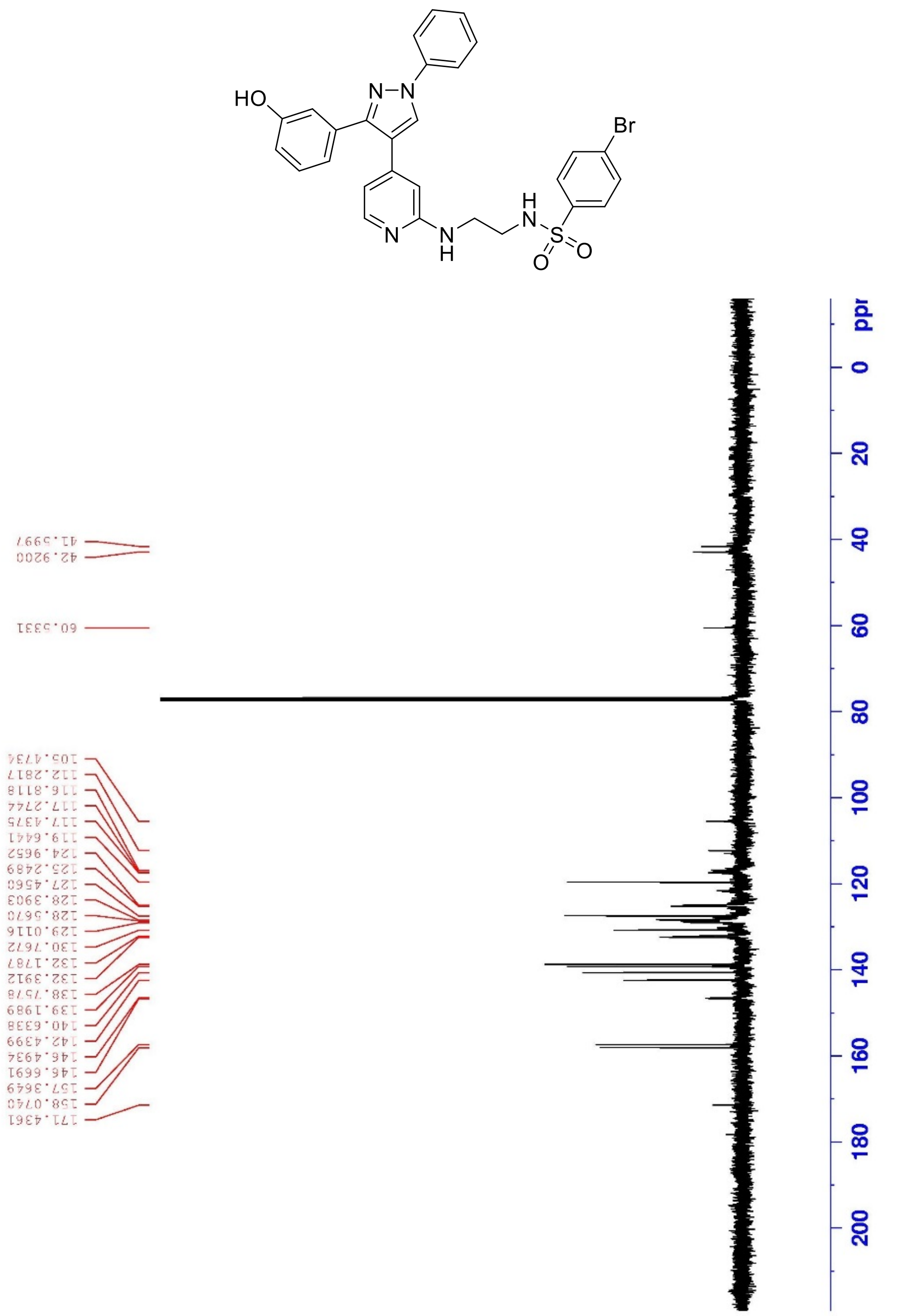

HO
N
N
Br
H
N
N
H
S
O
O
41.5997
42.9200
60.5331
105.4734
112.2817
116.8118
117.2744
117.4375
119.6441
124.9652
125.2489
127.4560
128.3903
128.5670
129.0116
130.7672
132.1787
132.3912
138.7578
139.1989
140.6338
142.4399
146.4934
146.6691
157.3649
158.0740
171.4361
ppm
0
20
40
60
80
100
120
140
160
180
200

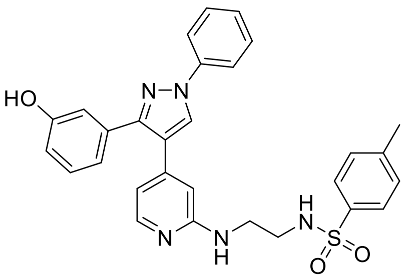
HO
N
N
N
N
H
H
N
S
O
O

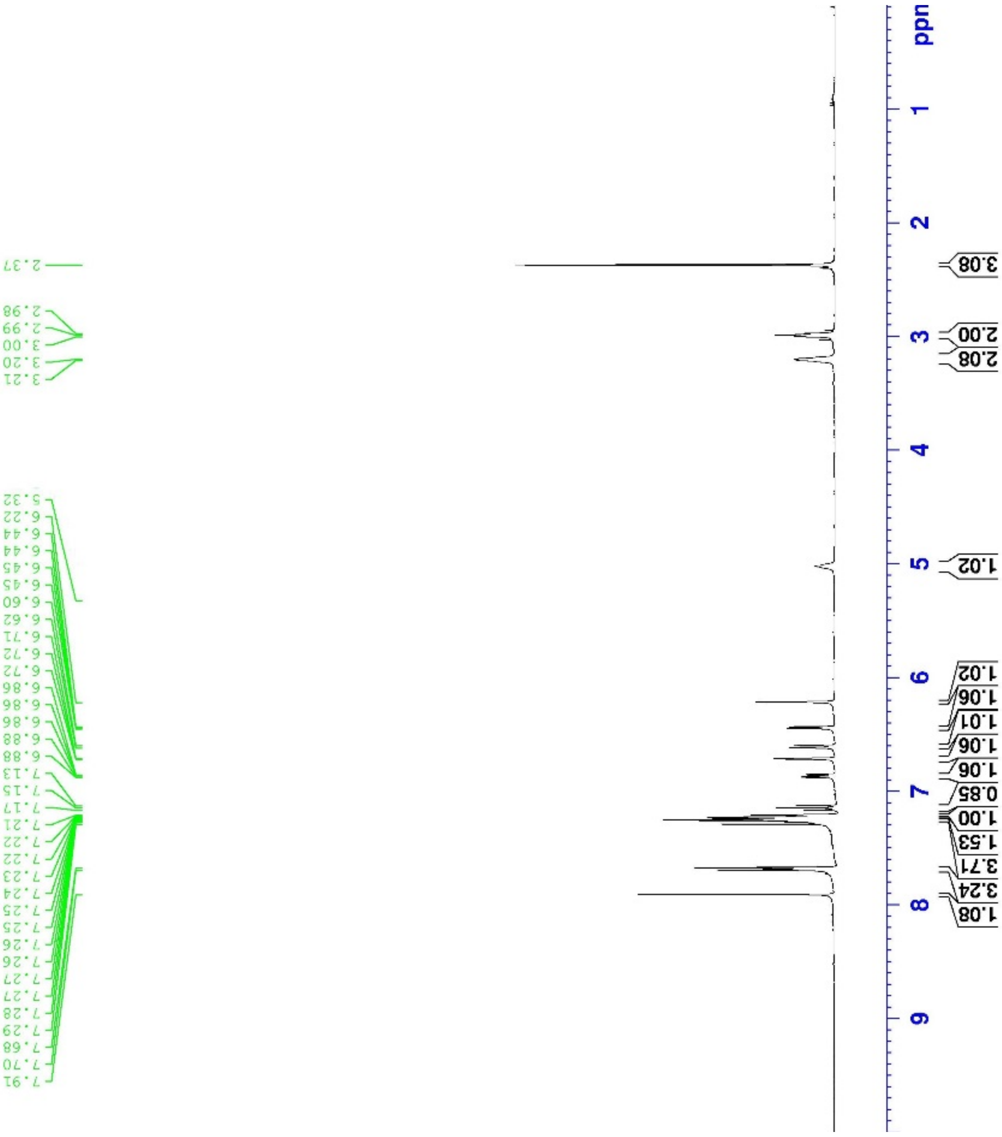
2.37
2.98
2.99
3.00
3.20
3.21
5.32
6.22
6.44
6.44
6.45
6.45
6.60
6.62
6.71
6.72
6.72
6.86
6.86
6.86
6.88
6.88
7.13
7.15
7.17
7.21
7.22
7.22
7.23
7.24
7.25
7.25
7.26
7.26
7.27
7.27
7.28
7.29
7.68
7.70
7.91
ppm
1
2
3
4
5
6
7
8
9
3.08
2.00
2.08
1.02
1.02
1.06
1.01
1.06
1.06
0.85
1.00
1.53
3.71
3.24
1.08

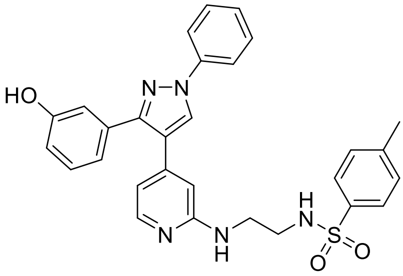
HO
N
N
N
H
N
H
N
S
O
O

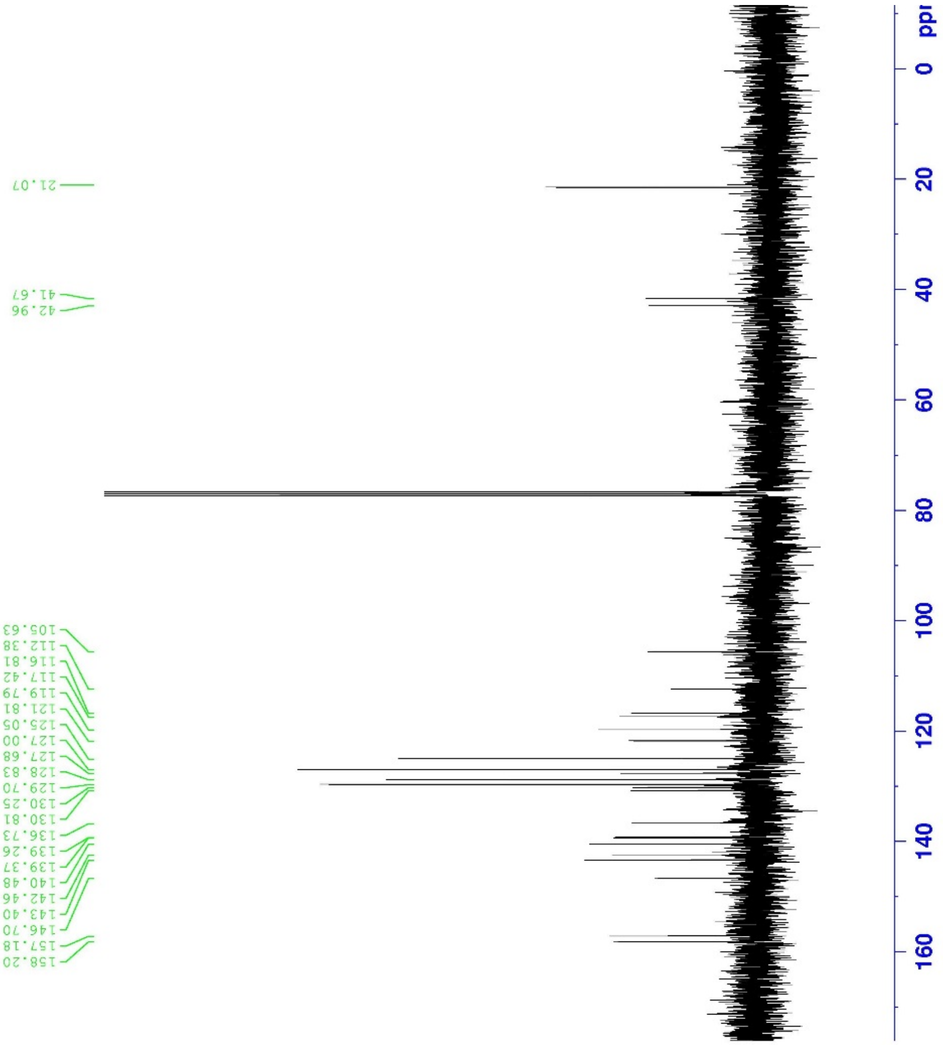
21.07
41.67
42.96
105.63
112.38
116.81
117.42
119.79
121.81
125.05
127.00
127.68
128.83
129.70
130.25
130.81
136.73
139.26
139.37
140.48
142.46
143.40
146.70
157.18
158.20
ppm
0
20
40
60
80
100
120
140
160

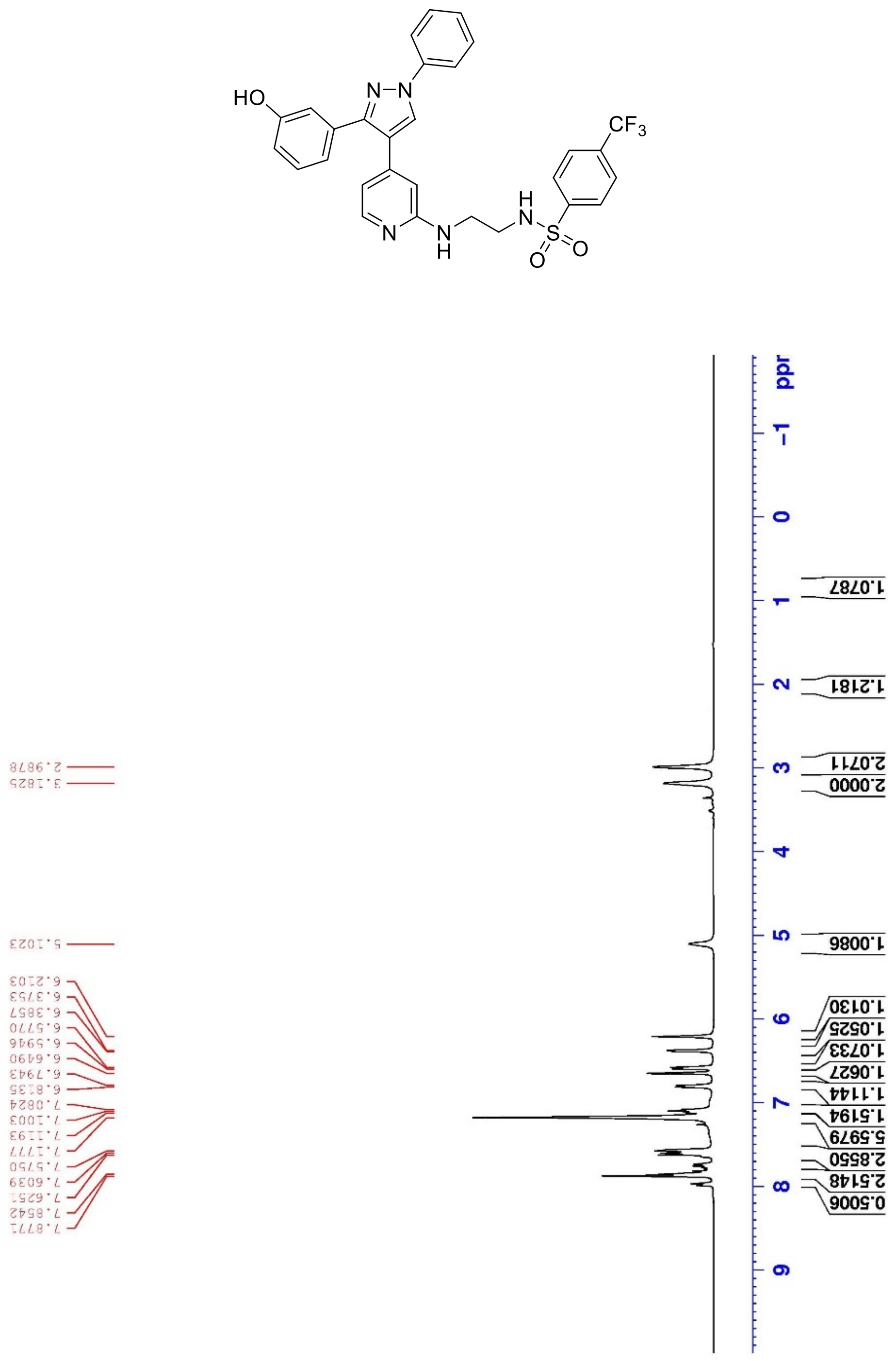
HO
N
N
CF3
N
H
N
H
S
O
O
ppm

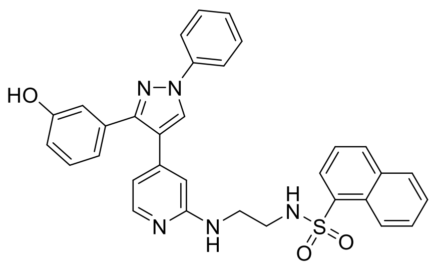

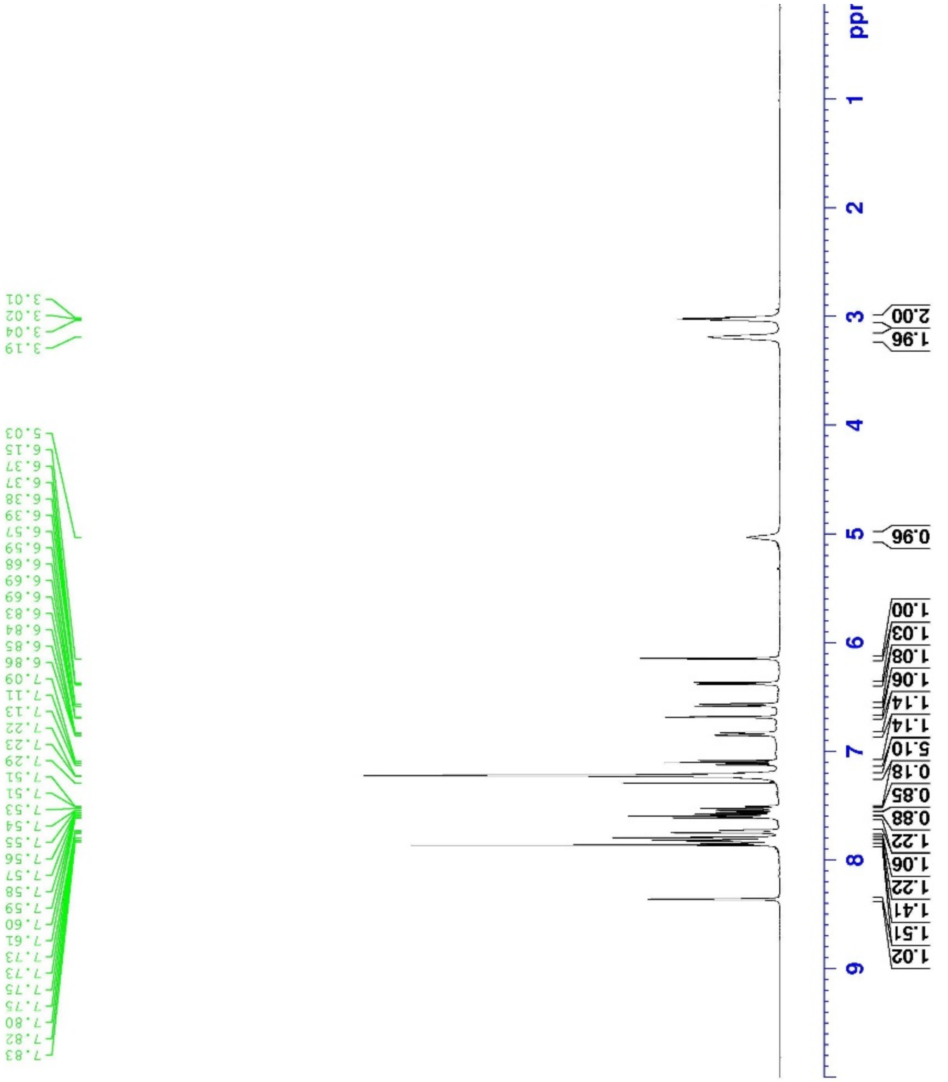

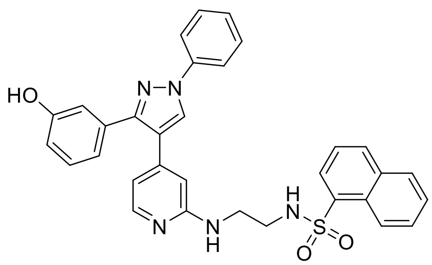
HO
N
N
N
H
N
H
S
O
O

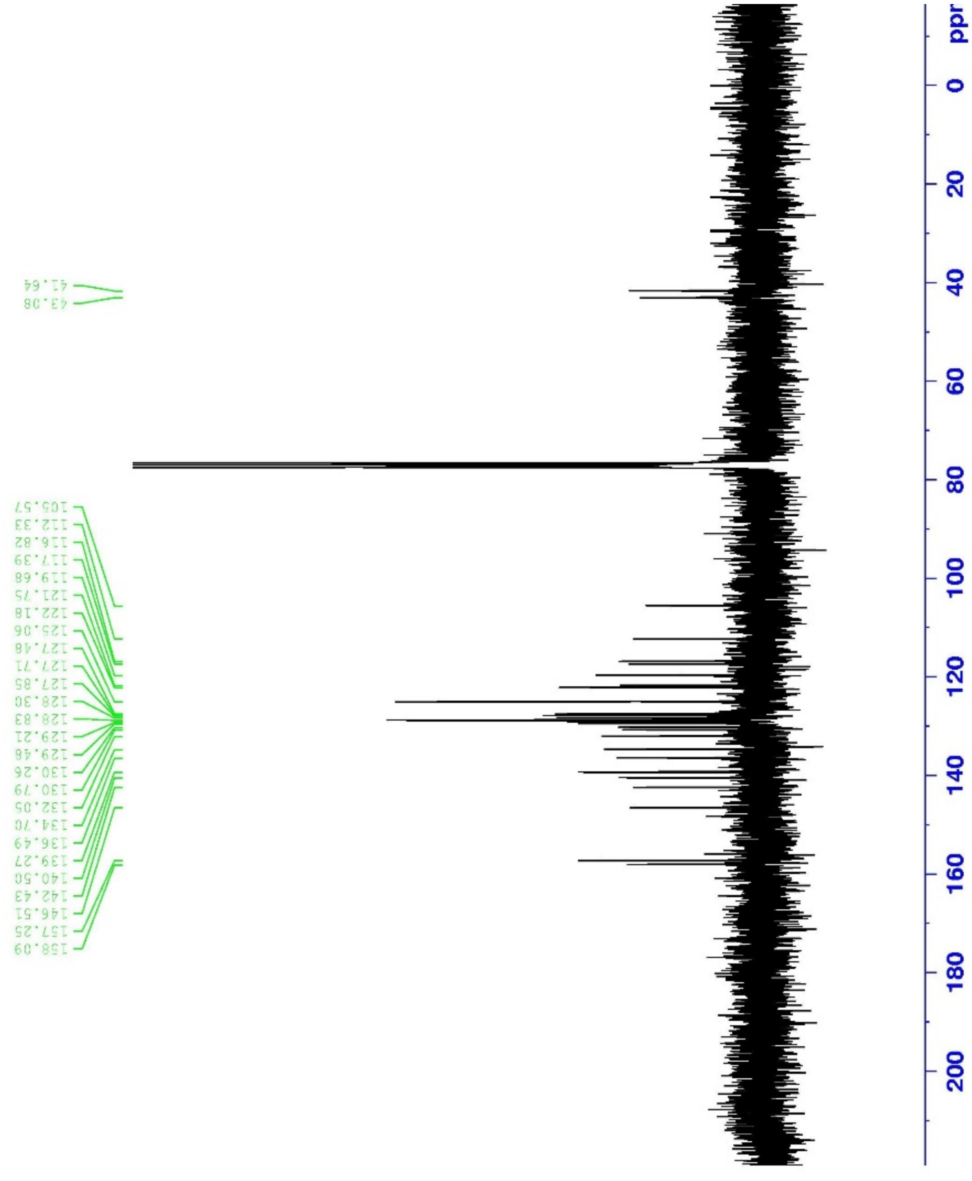
41.64
43.08
105.57
112.33
116.82
117.39
119.68
121.75
122.18
125.06
127.48
127.71
127.85
128.30
128.83
129.21
129.48
130.26
130.79
132.05
134.70
136.49
139.27
140.50
142.43
146.51
157.25
158.09
ppm
0
20
40
60
80
100
120
140
160
180
200

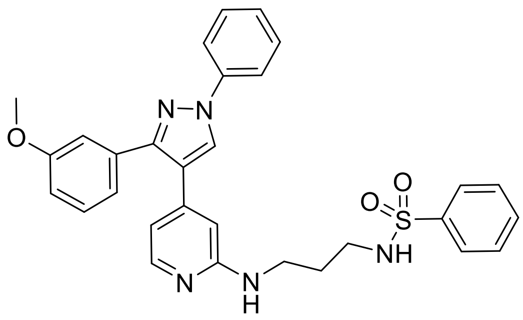

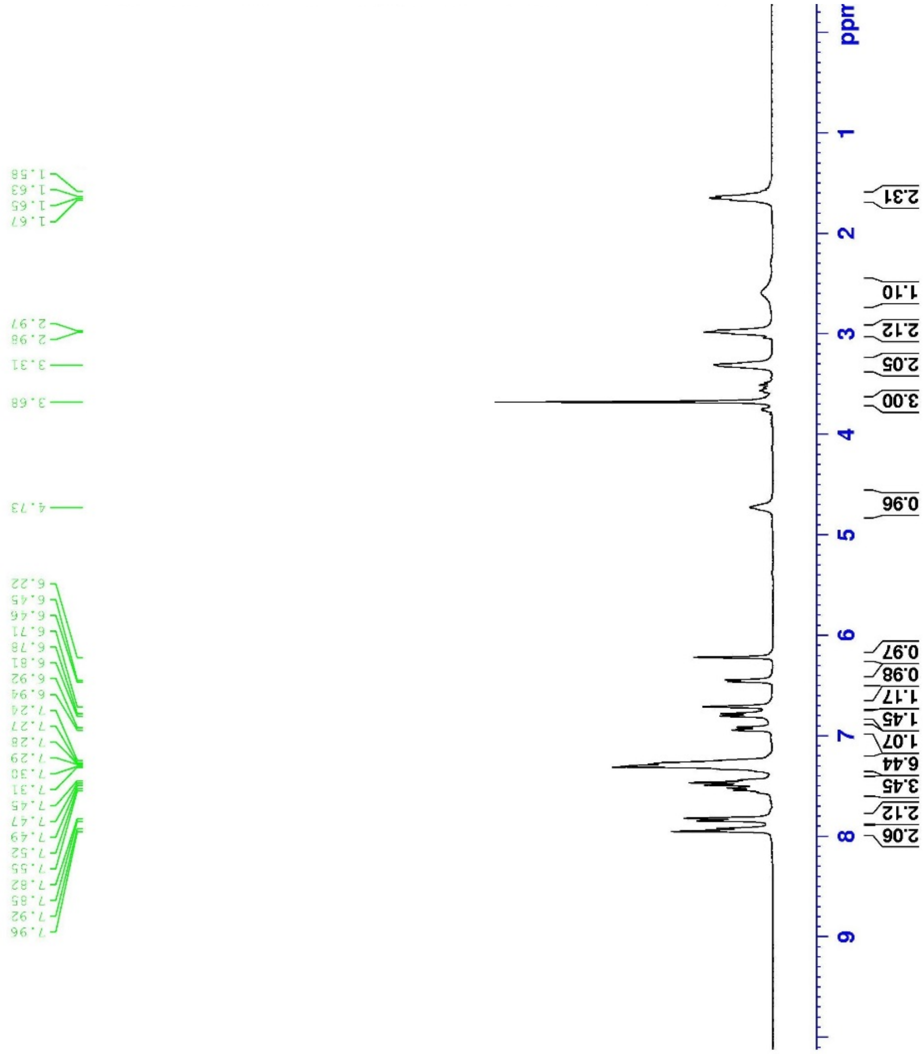

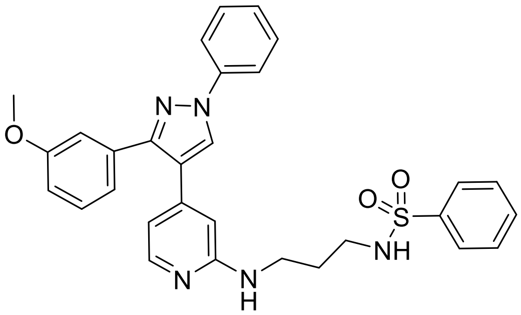

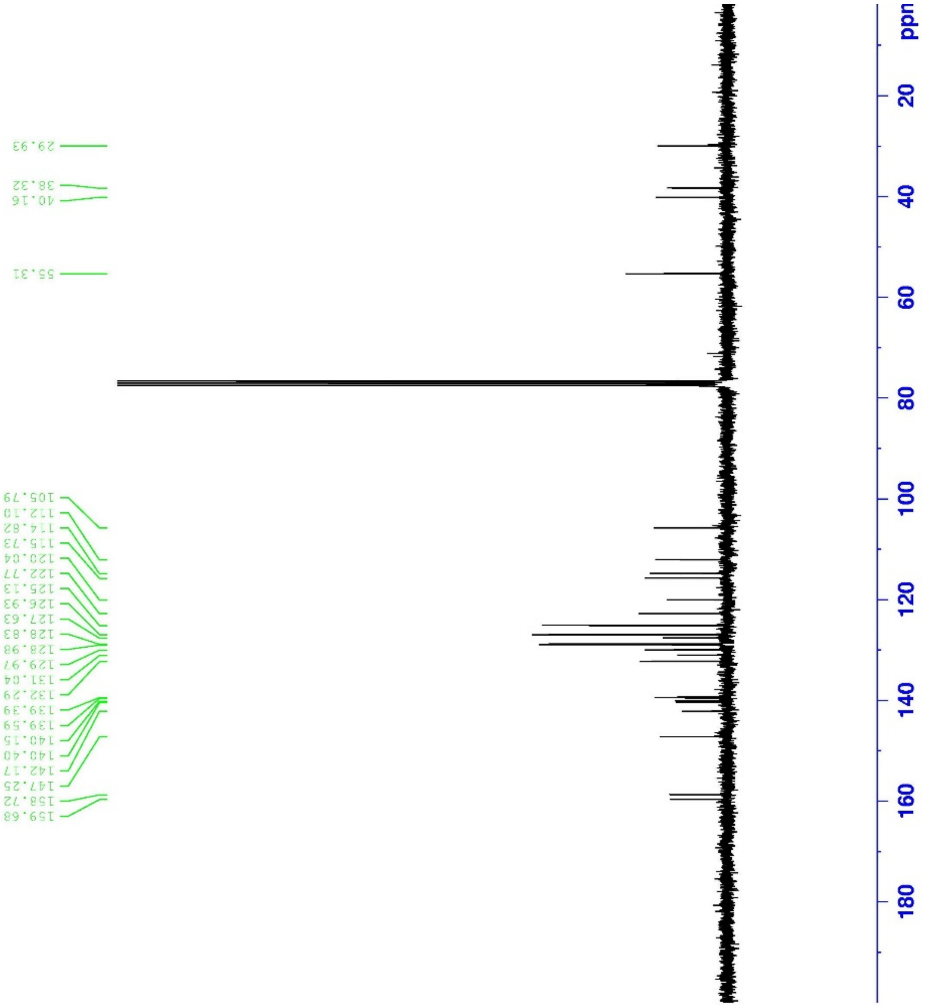
29.93
38.32
40.16
55.31
105.79
112.10
114.82
115.73
120.04
122.77
125.13
126.93
127.63
128.83
128.98
129.97
131.04
132.29
139.39
139.59
140.15
140.40
142.17
147.25
158.72
159.68
ppm
20
40
60
80
100
120
140
160
180

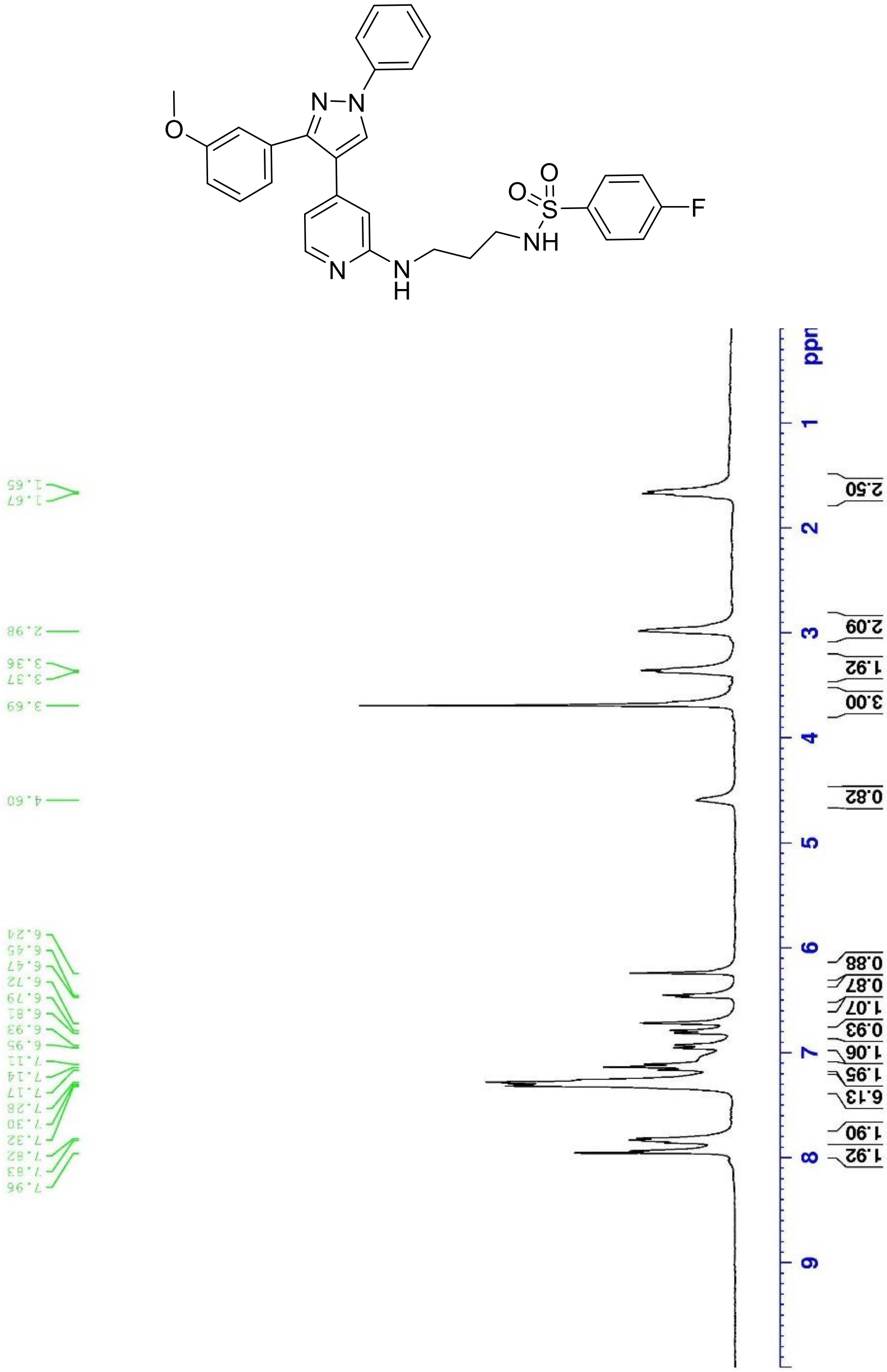

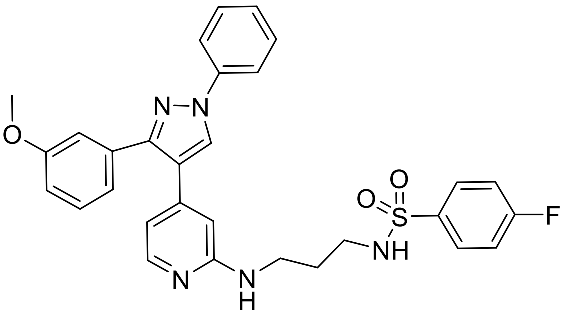

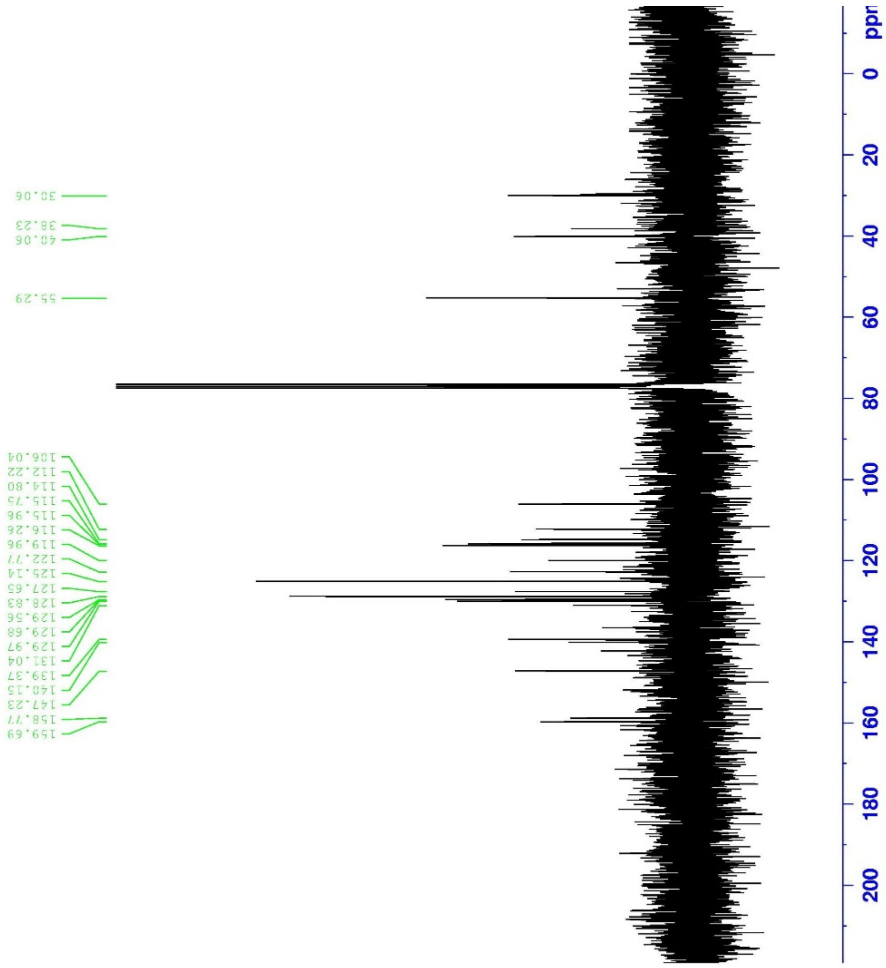
30.06
38.23
40.06
55.29
106.04
112.22
114.80
115.75
115.96
116.26
119.96
122.77
125.14
127.65
128.83
129.56
129.68
129.97
131.04
139.37
140.15
147.23
158.77
159.69
ppm
0
20
40
60
80
100
120
140
160
180
200

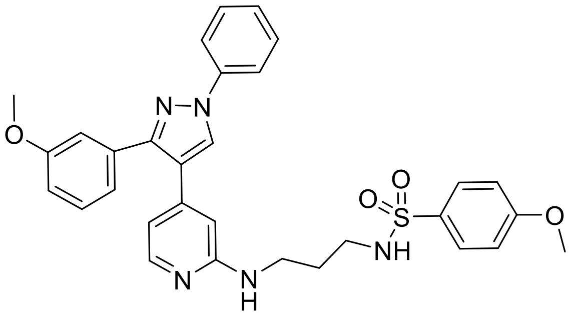

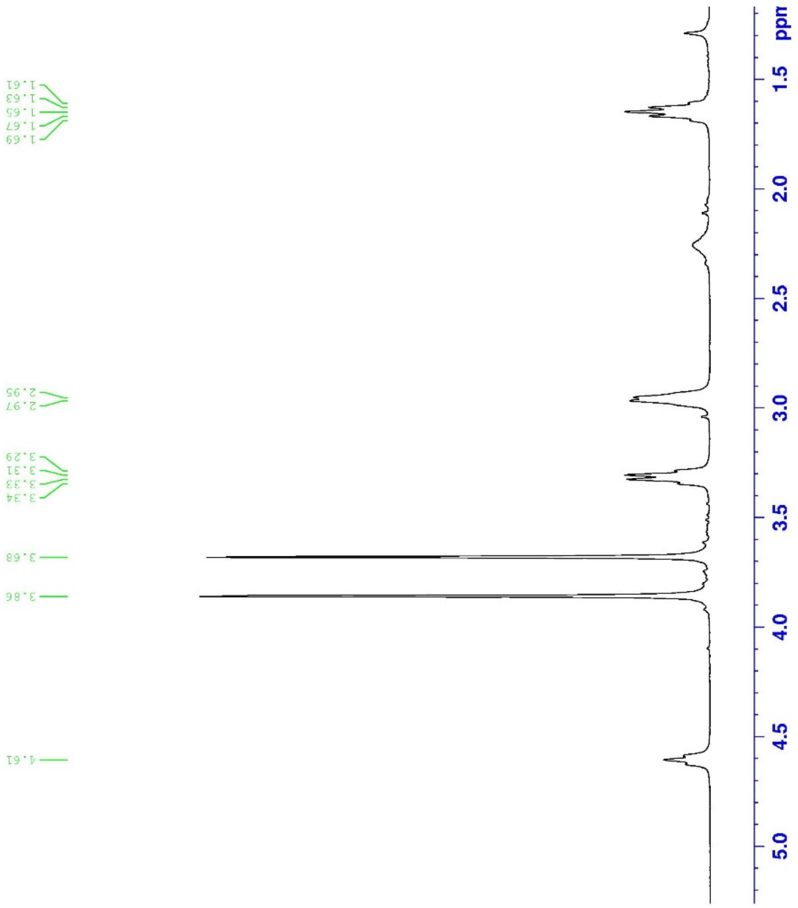
ppm
1.5
2.0
2.5
3.0
3.5
4.0
4.5
5.0
1.61
1.63
1.65
1.67
1.69
2.95
2.97
3.29
3.31
3.33
3.34
3.68
3.86
4.61

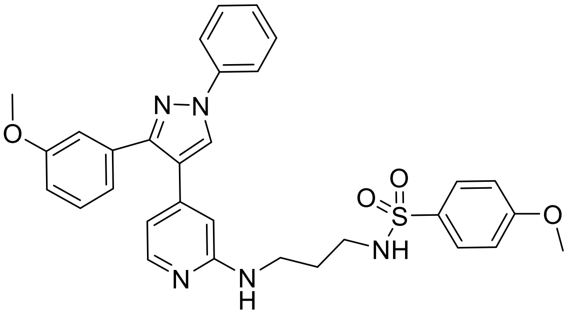

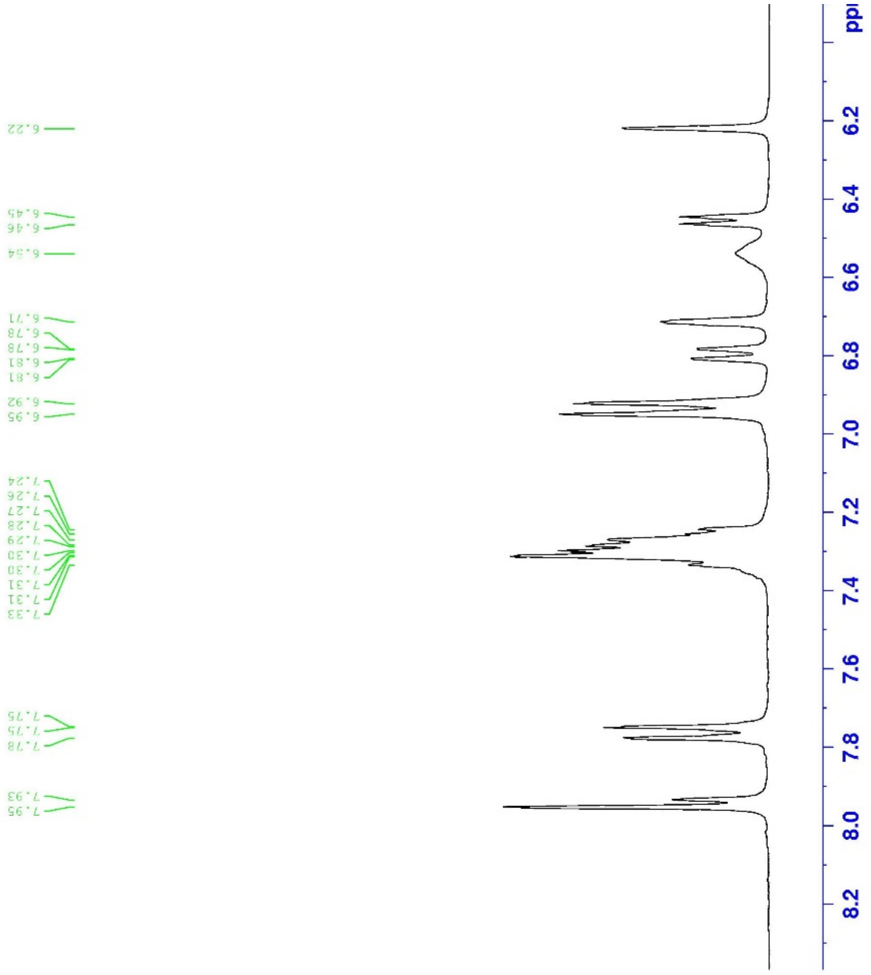
ppm
6.2
6.4
6.6
6.8
7.0
7.2
7.4
7.6
7.8
8.0
8.2

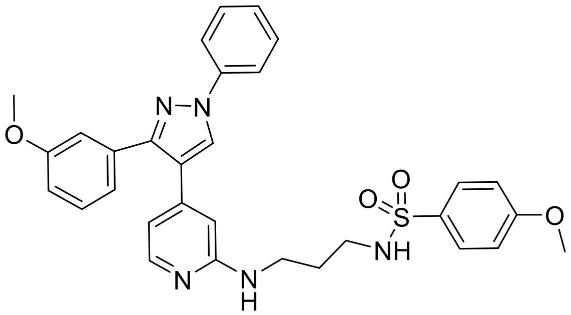

N
N
O
O
O
S
NH
O
N
N
H

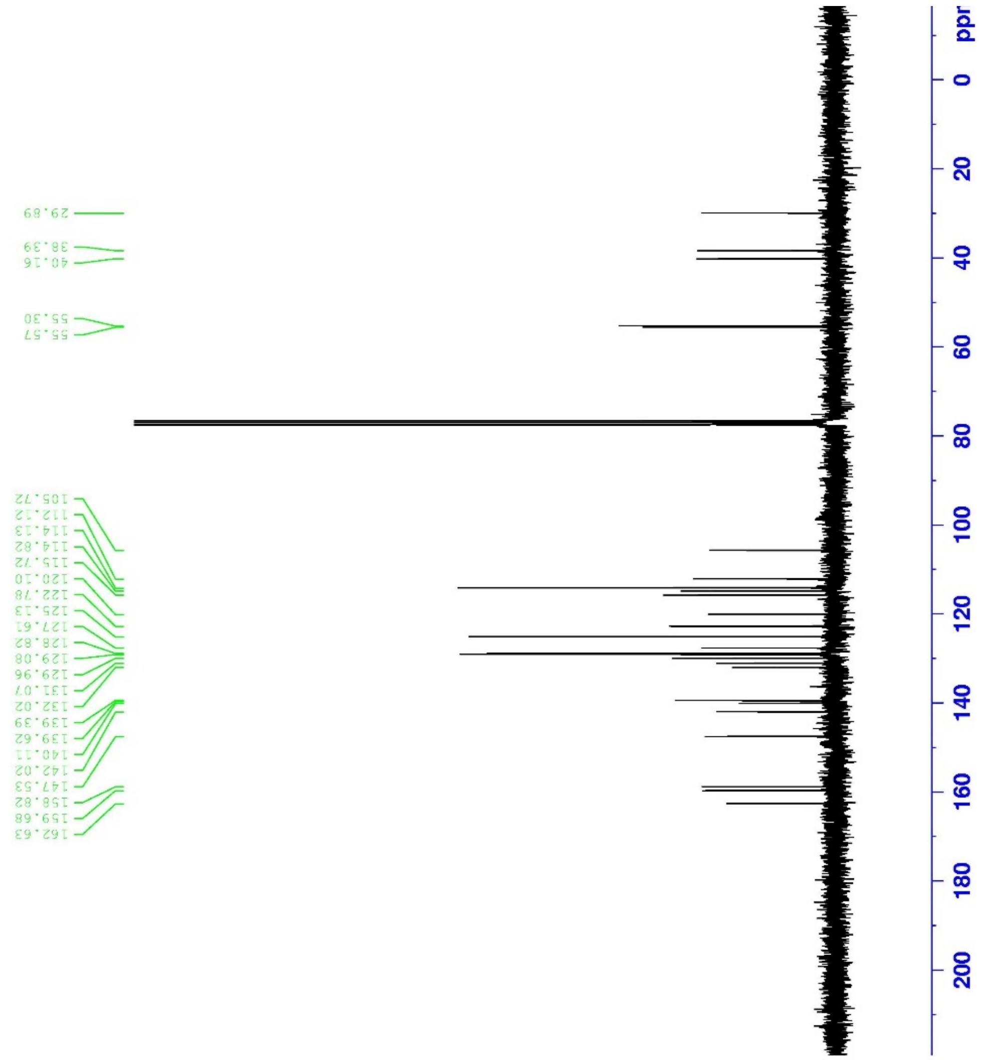

29.89
38.39
40.16
55.30
55.57
105.72
112.12
114.13
114.82
115.72
120.10
122.78
125.13
127.61
128.82
129.08
129.96
131.07
132.02
139.39
139.62
140.11
142.02
147.53
158.82
159.68
162.63
ppm
0
20
40
60
80
100
120
140
160
180
200

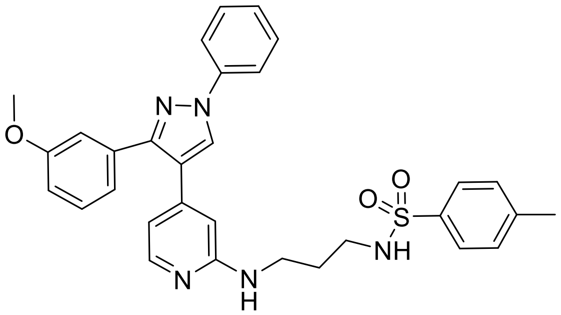
O
N
N
O
O
S
NH
N
N
H

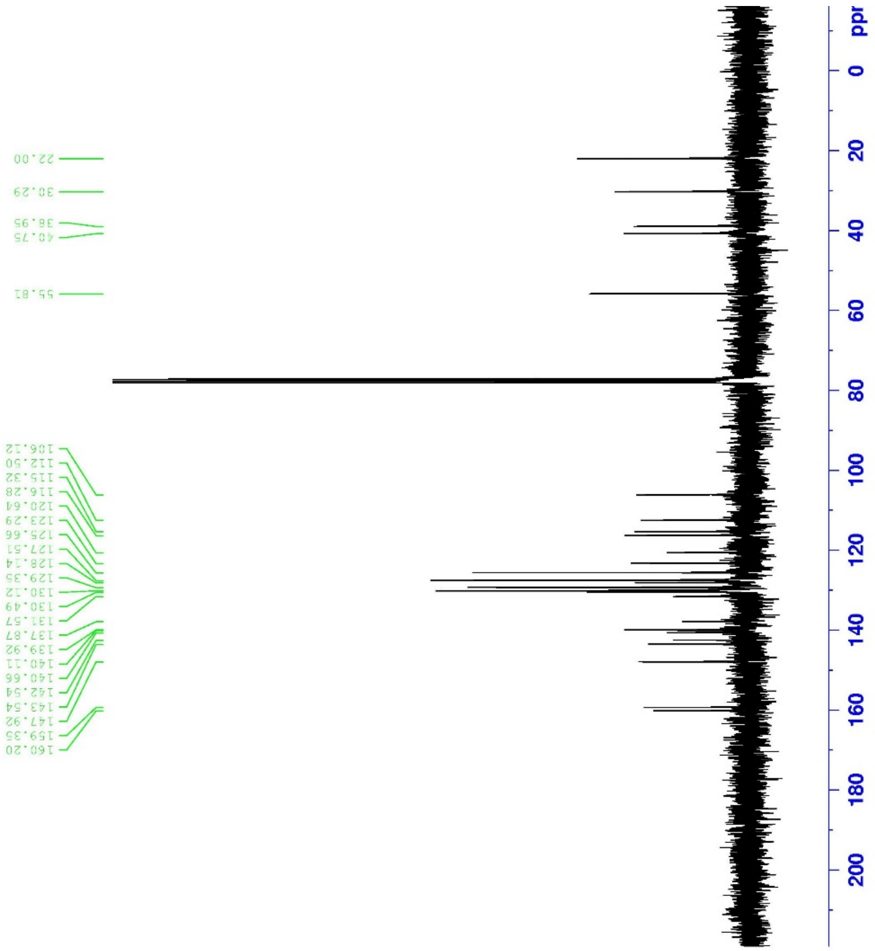
22.00
30.29
38.95
40.75
55.81
106.12
112.50
115.32
116.28
120.61
123.29
125.66
127.51
128.14
129.35
130.12
130.49
131.57
137.87
139.92
140.11
140.66
142.54
143.54
147.92
159.35
160.20
ppm
0
20
40
60
80
100
120
140
160
180
200

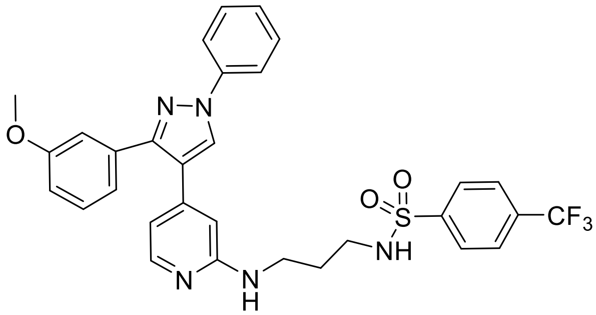
O
N
N
O
O
S
CF_3
NH
N
H

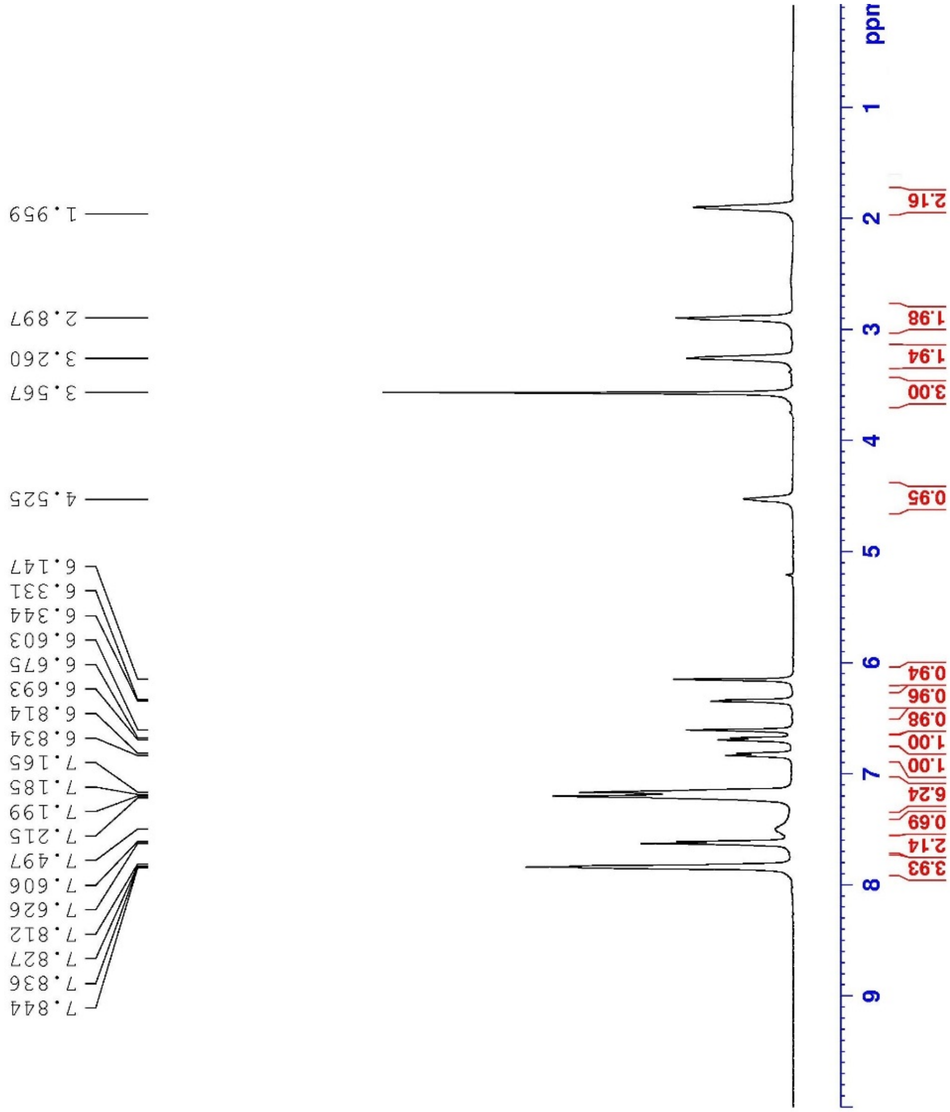
1.959
2.897
3.260
3.567
4.525
6.147
6.331
6.344
6.603
6.675
6.693
6.814
6.834
7.165
7.185
7.199
7.215
7.497
7.606
7.626
7.812
7.827
7.836
7.844
ppm
1
2
3
4
5
6
7
8
9
2.16
1.98
1.94
3.00
0.95
0.94
0.96
0.98
1.00
1.00
6.24
0.69
2.14
3.93

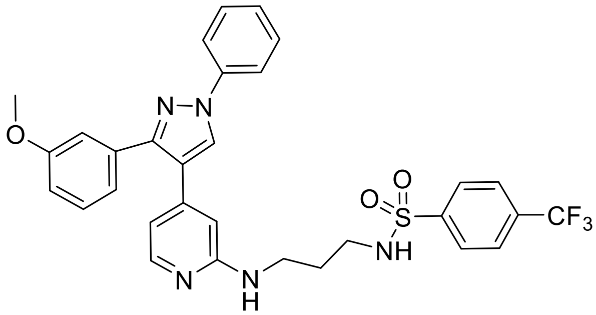
O
N
N
O
O
S
CF3
NH
N
H
N

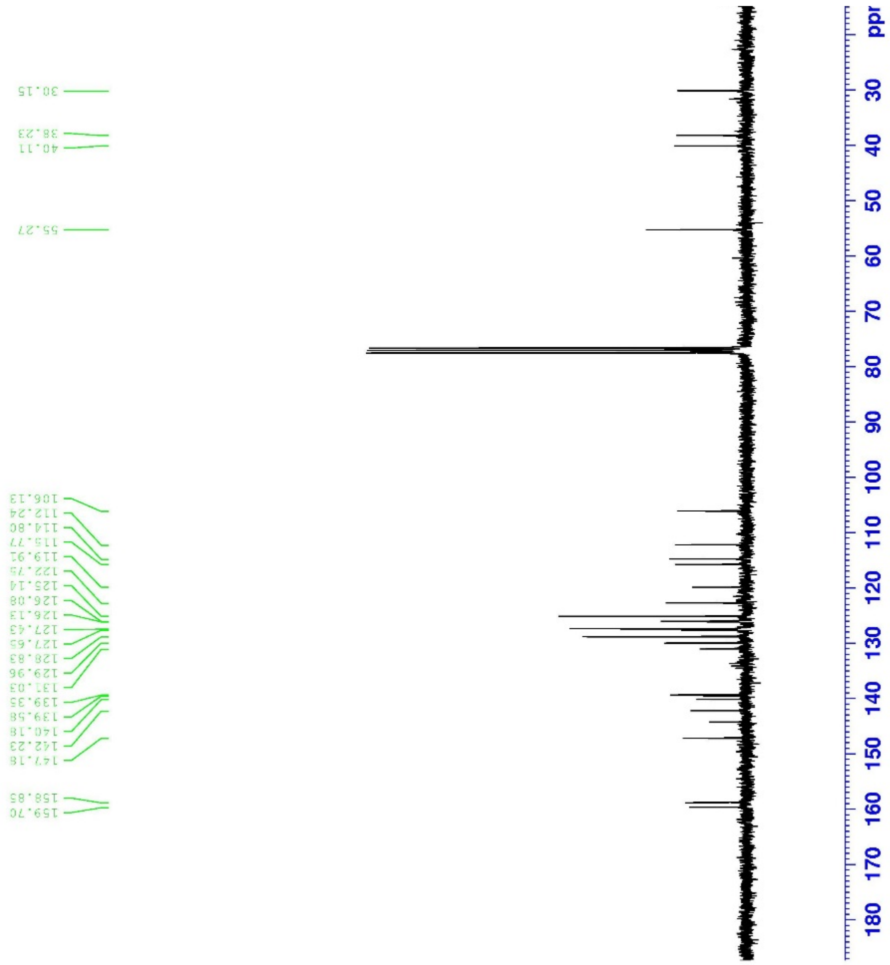
30.15
38.23
40.11
55.27
106.13
112.24
114.80
115.77
119.91
122.75
125.14
126.08
126.13
127.43
127.65
128.83
129.96
131.03
139.35
139.58
140.18
142.23
147.18
158.85
159.70
ppm
30
40
50
60
70
80
90
100
110
120
130
140
150
160
170
180

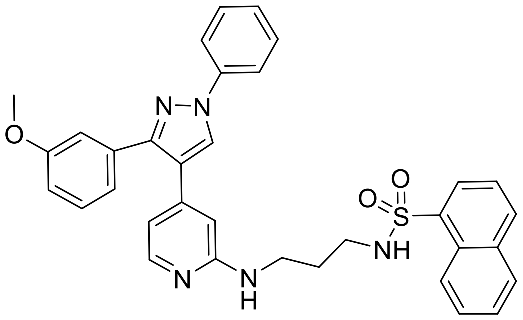

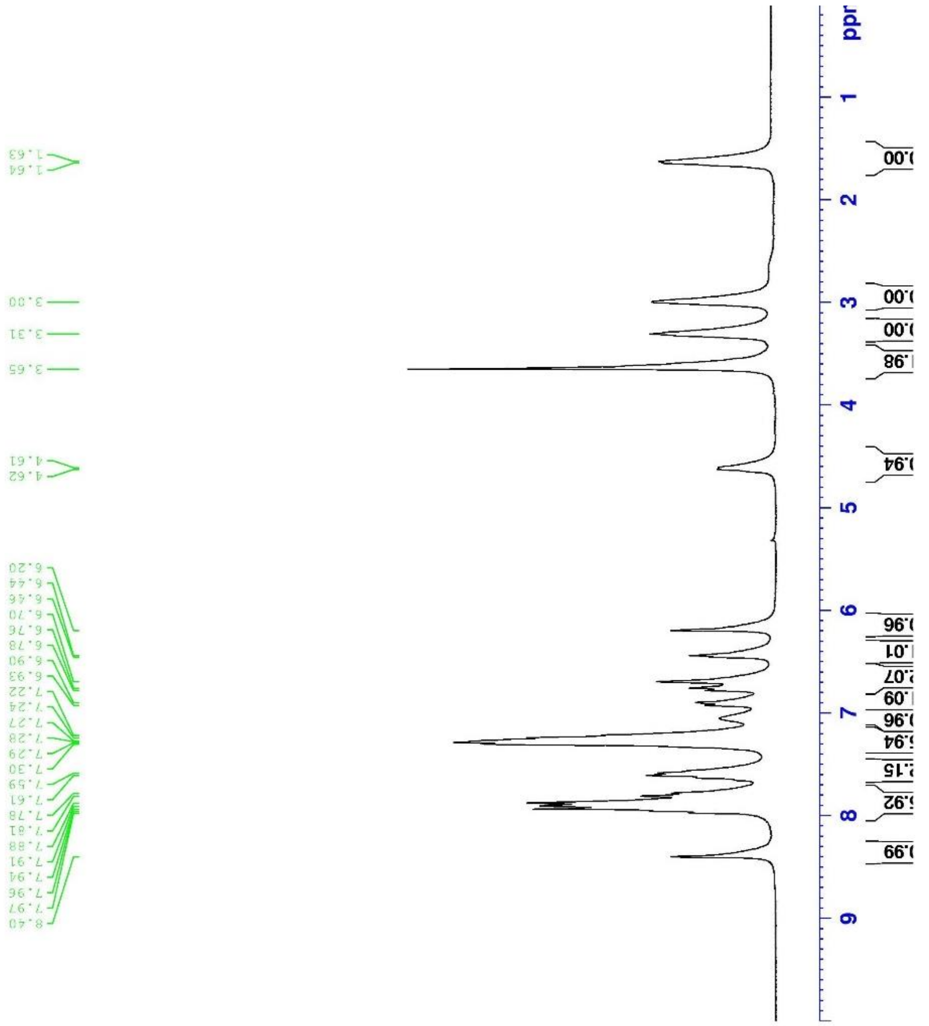

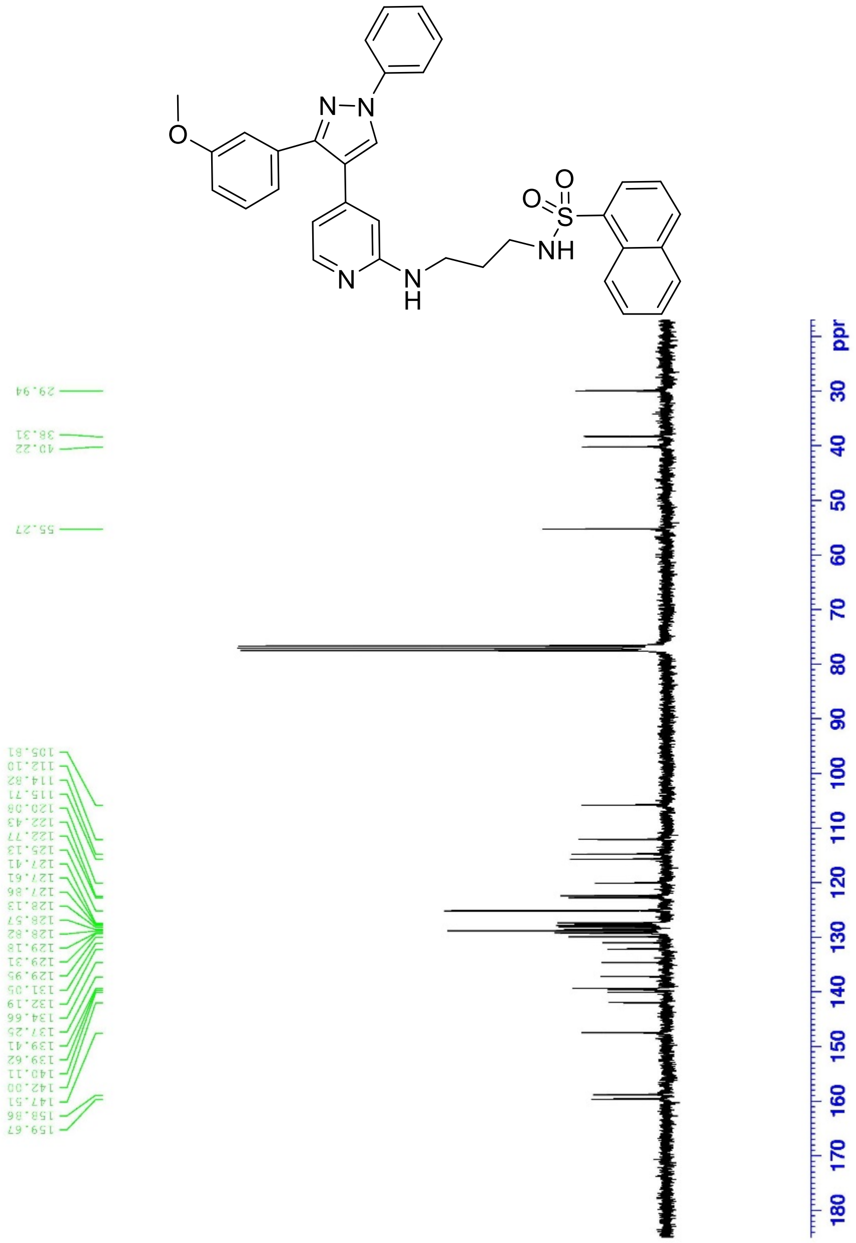
O
N
N
N
H
NH
S
O
O
29.94
38.31
40.22
55.27
105.81
112.10
114.82
115.71
120.08
122.43
122.77
125.13
127.41
127.61
127.86
128.13
128.57
128.82
129.18
129.31
129.95
131.05
132.19
134.66
137.25
139.41
139.62
140.11
142.00
147.51
158.86
159.67
ppm

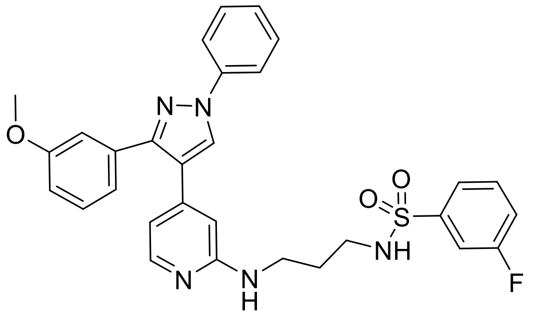
O
N
N
O
O
S
NH
N
N
H
F

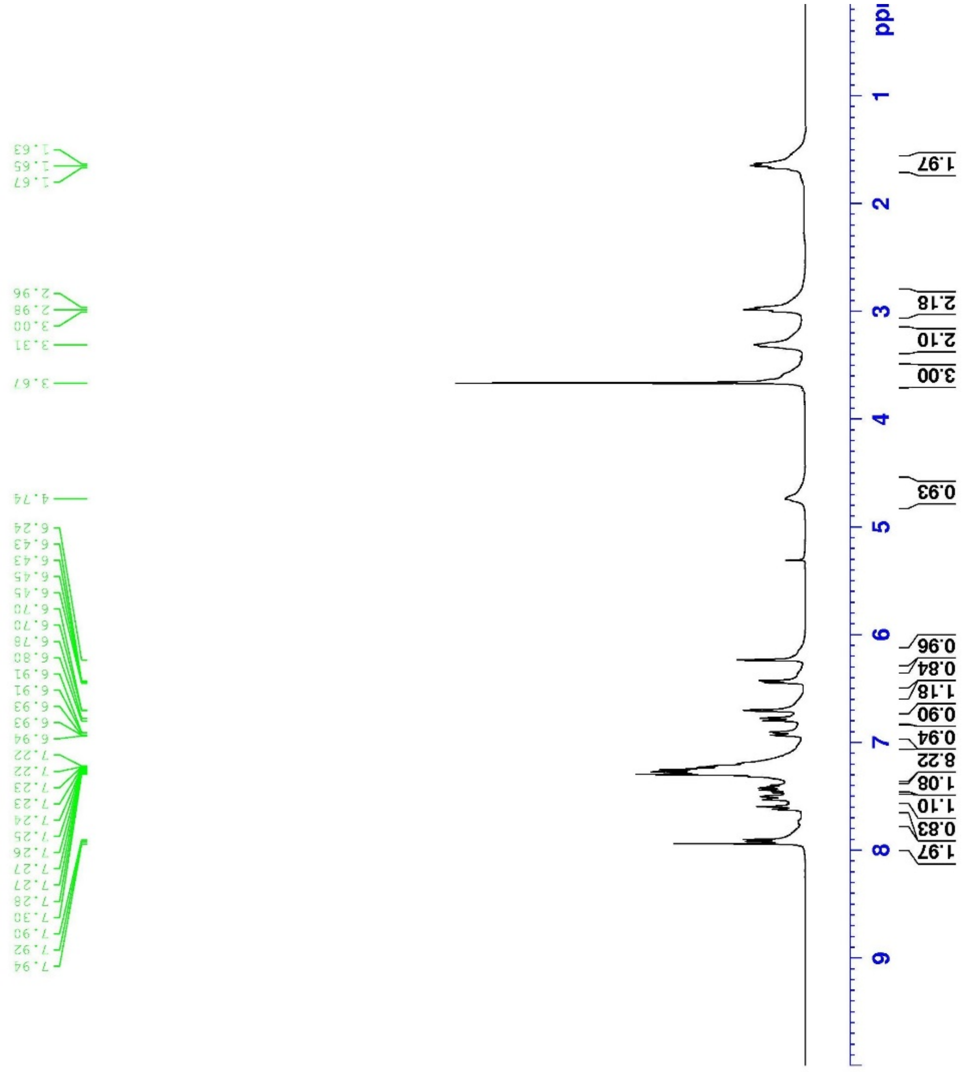
1.63
1.65
1.67
2.96
2.98
3.00
3.31
3.67
4.74
6.24
6.43
6.43
6.45
6.45
6.70
6.70
6.78
6.80
6.91
6.91
6.93
6.93
6.94
7.22
7.22
7.22
7.23
7.23
7.24
7.25
7.26
7.27
7.27
7.28
7.30
7.90
7.92
7.94
1.97
2.18
2.10
3.00
0.93
0.96
0.84
1.18
0.90
0.94
8.22
1.08
1.10
0.83
1.97
1
2
3
4
5
6
7
8
9
ppm

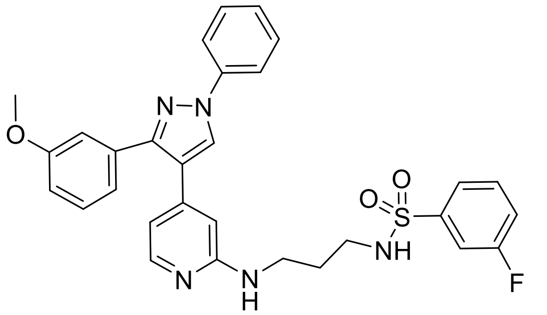

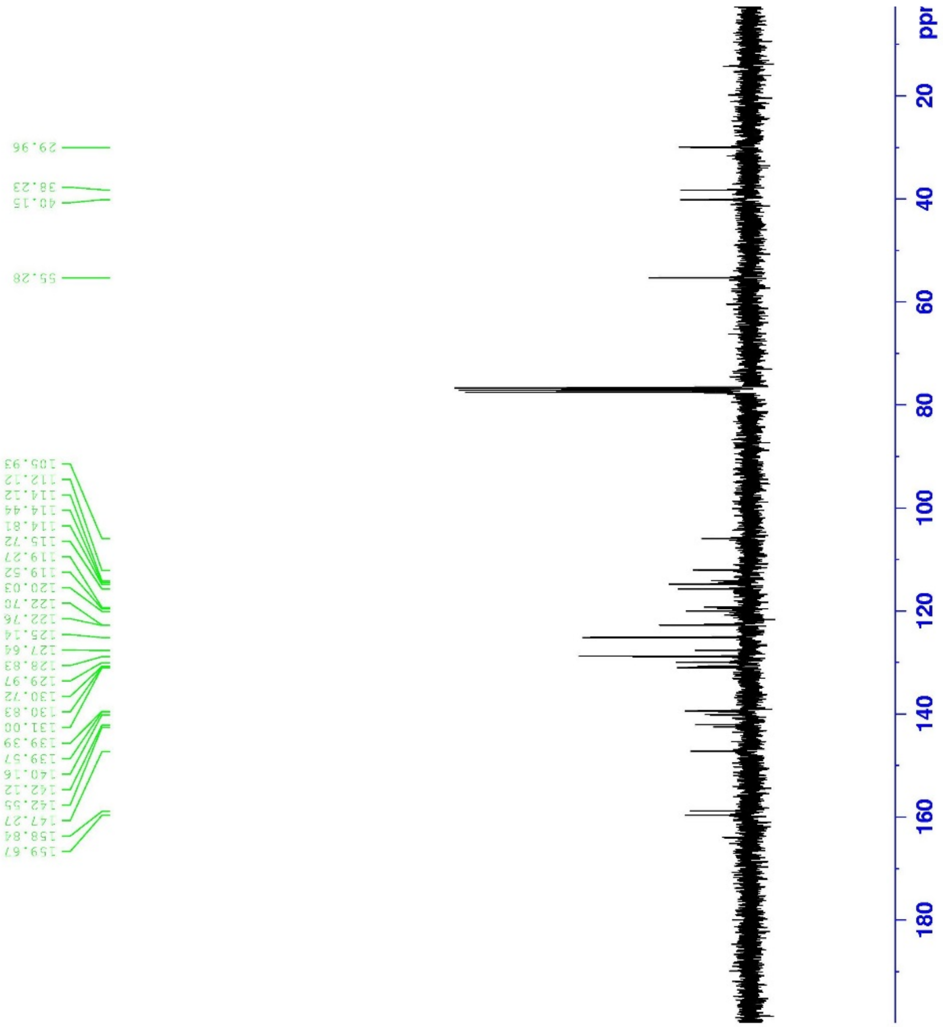

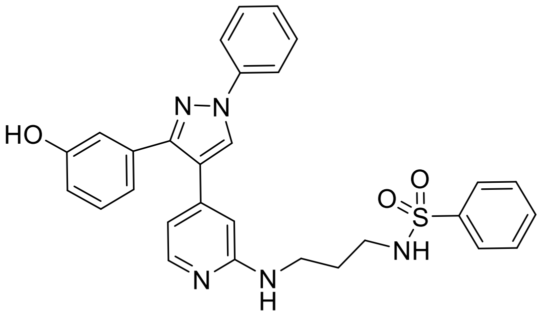
HO
N
N
O
O
S
NH
N
N
H

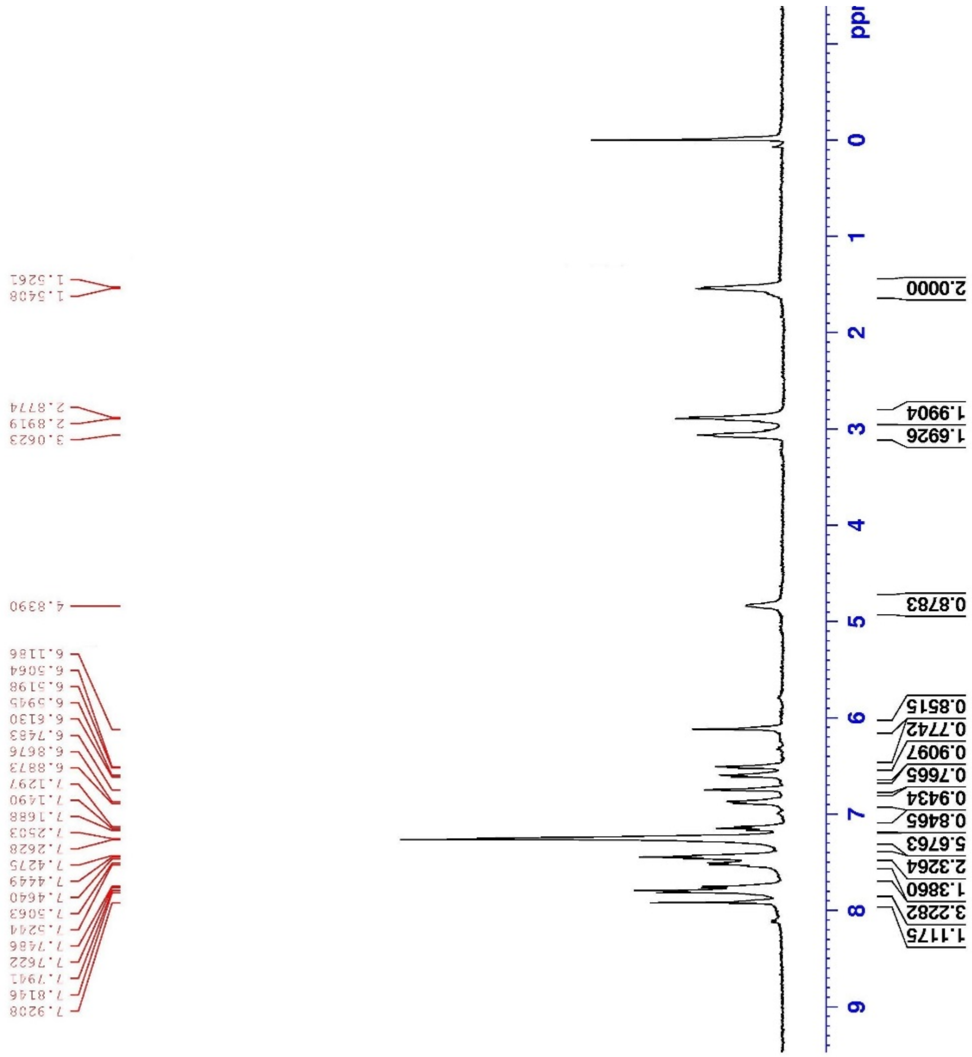

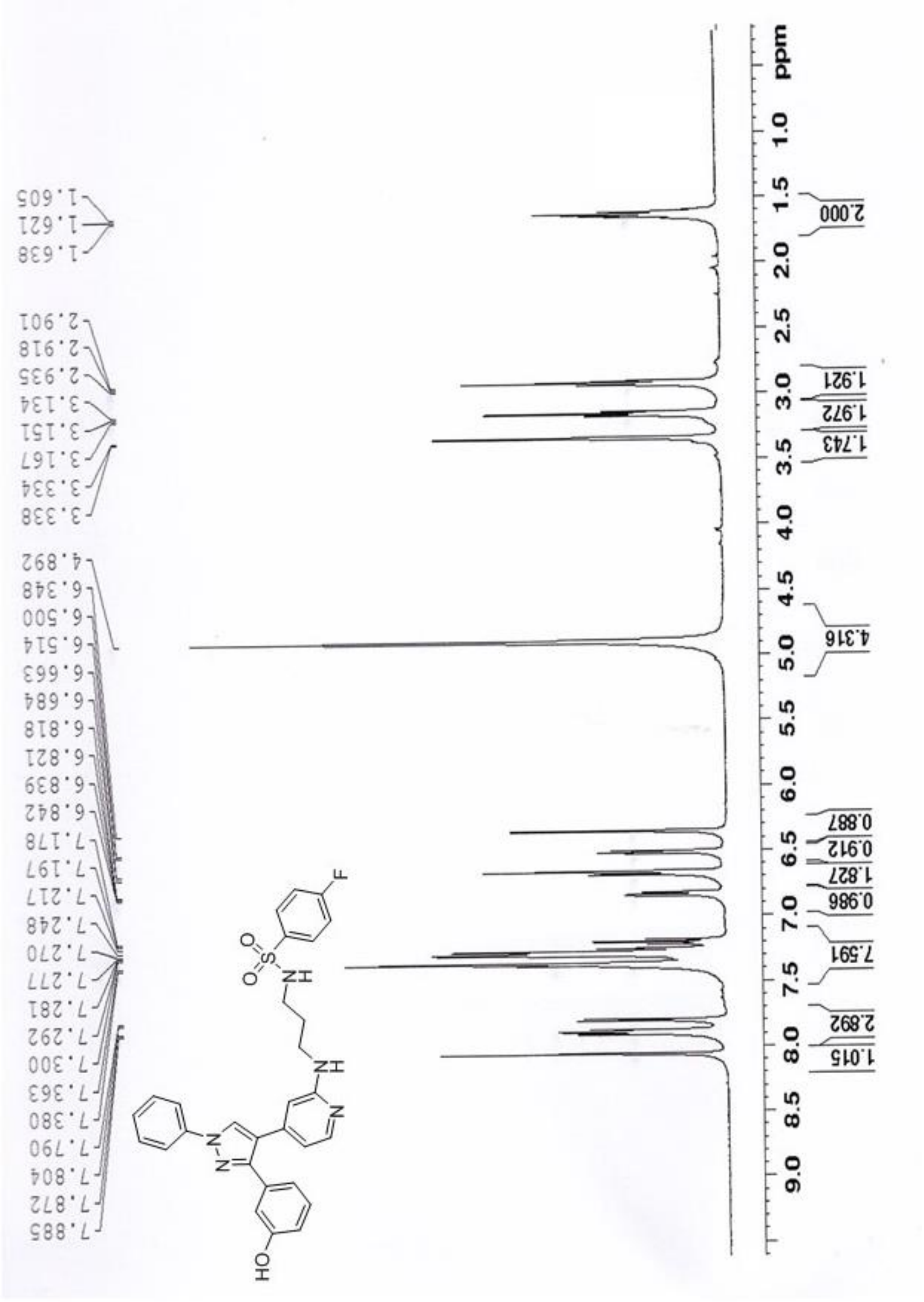
1.605
1.621
1.638
2.901
2.918
2.935
3.134
3.151
3.167
3.334
3.338
4.892
6.348
6.500
6.514
6.663
6.684
6.818
6.821
6.839
6.842
7.178
7.197
7.217
7.248
7.270
7.277
7.281
7.292
7.300
7.363
7.380
7.790
7.804
7.872
7.885
2.000
1.921
1.972
1.743
4.316
0.887
0.912
1.827
0.986
7.591
2.892
1.015
ppm
1.0
1.5
2.0
2.5
3.0
3.5
4.0
4.5
5.0
5.5
6.0
6.5
7.0
7.5
8.0
8.5
9.0
HO
N
NH
S
O
F

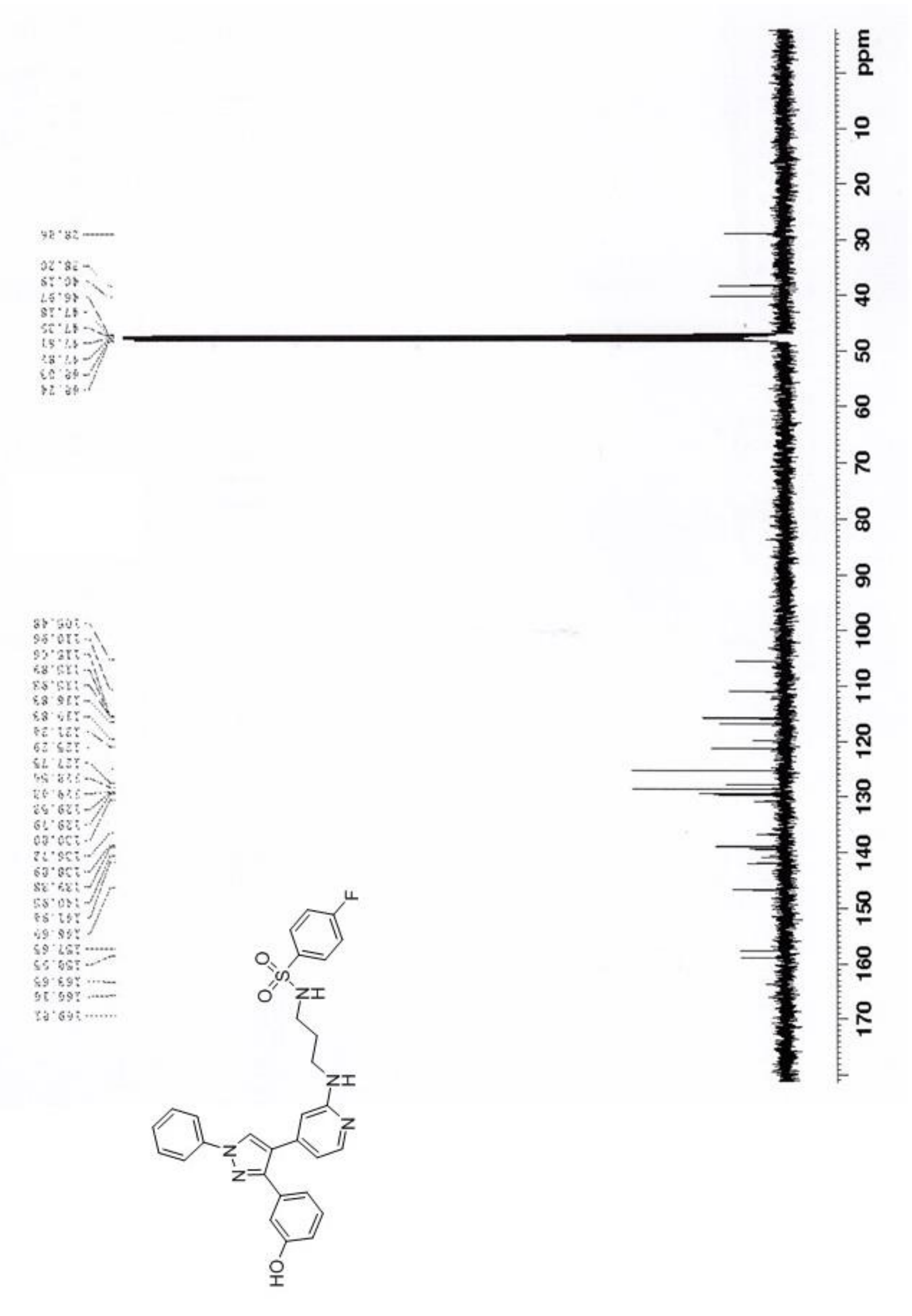

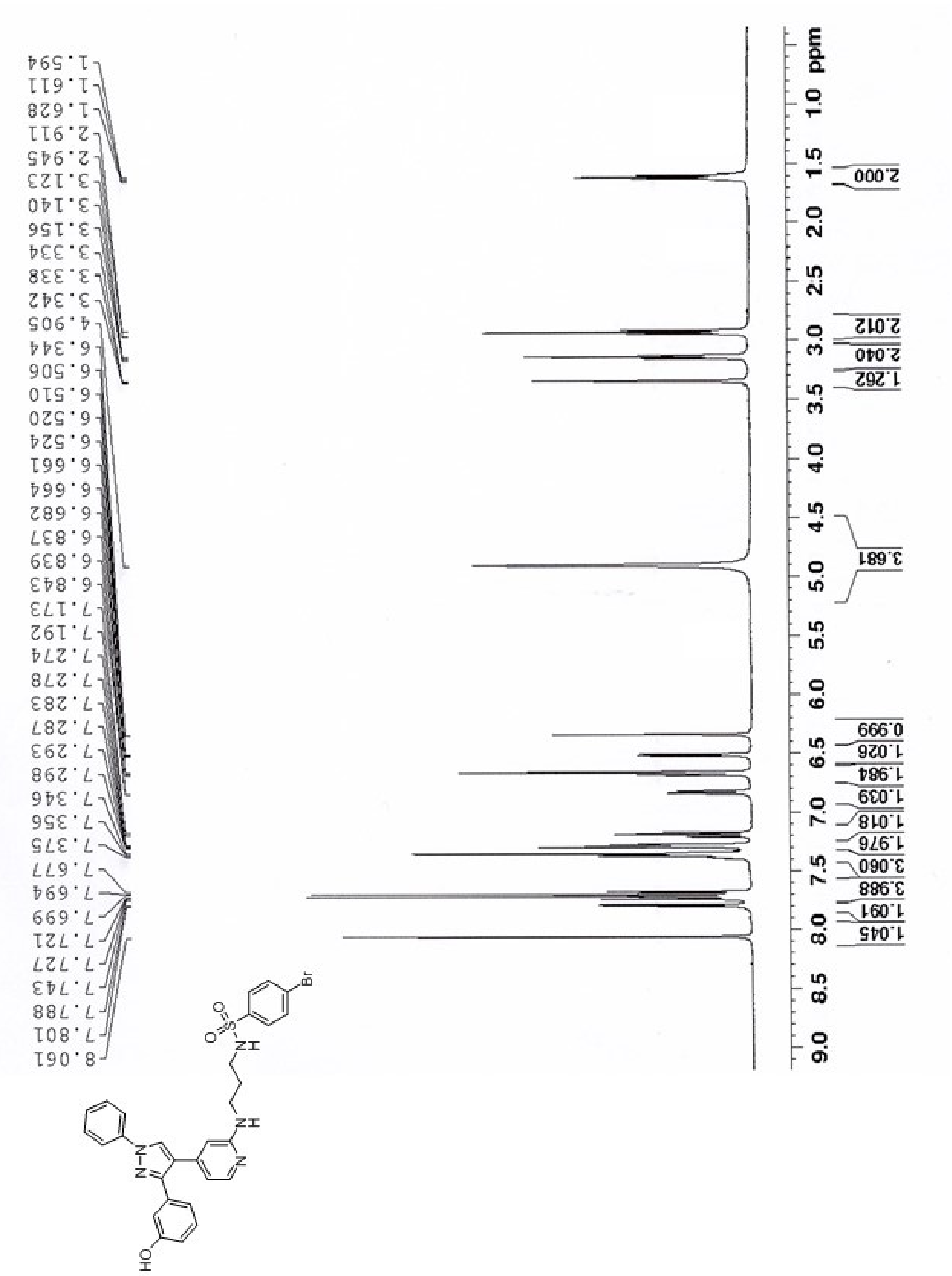

1.594
1.611
1.628
2.911
2.945
3.123
3.140
3.156
3.334
3.338
3.342
4.905
6.344
6.506
6.510
6.520
6.524
6.661
6.664
6.682
6.837
6.839
6.843
7.173
7.192
7.274
7.278
7.283
7.287
7.293
7.298
7.346
7.356
7.375
7.677
7.694
7.699
7.721
7.727
7.743
7.788
7.801
8.061
ppm
2.000
2.012
2.040
1.262
3.681
0.999
1.026
1.984
1.039
1.018
1.976
3.060
3.988
1.091
1.045

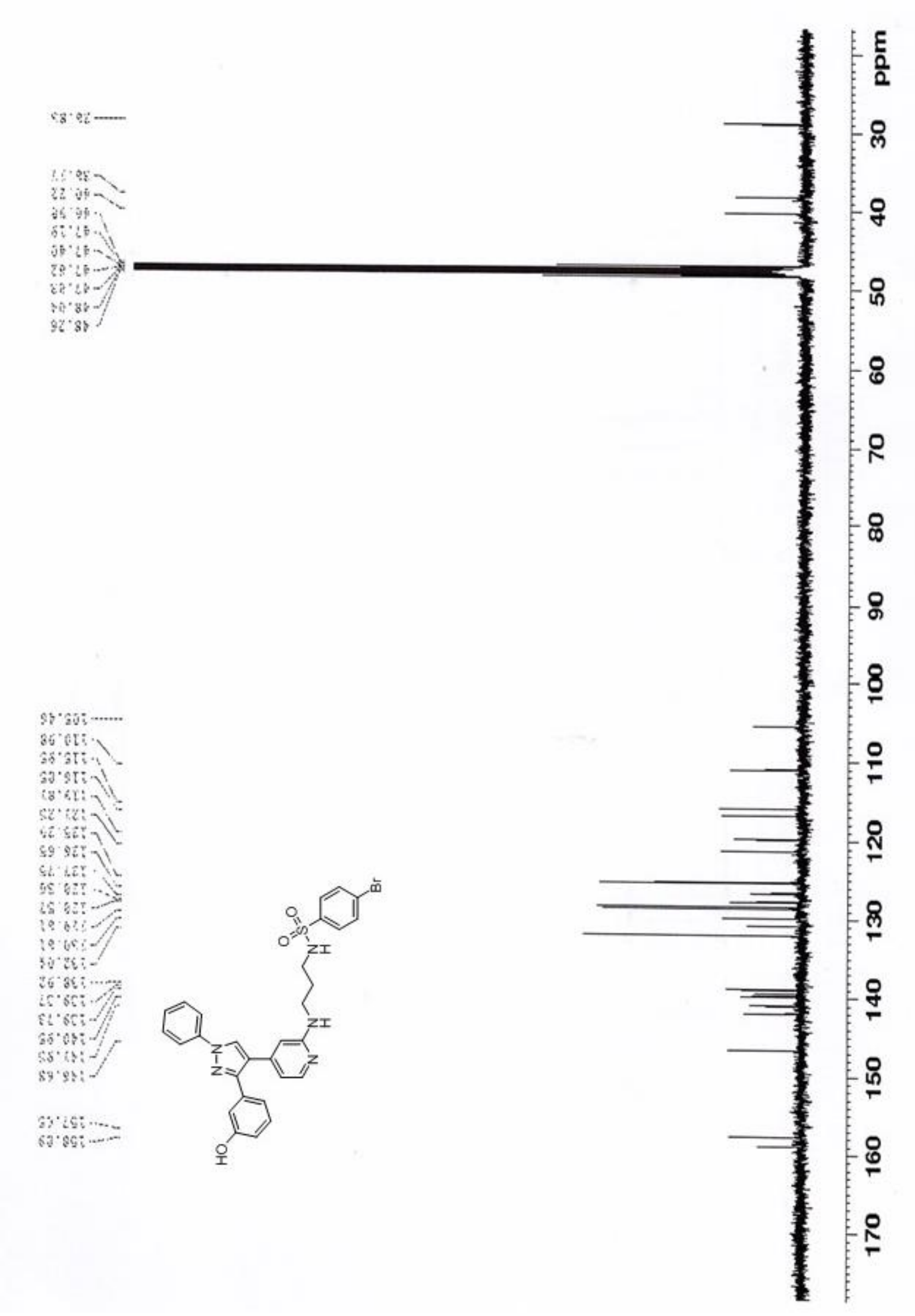
ppm
30
40
50
60
70
80
90
100
110
120
130
140
150
160
170

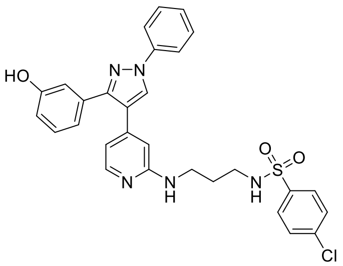
HO
N
N
N
H
N
H
S
O
O
Cl

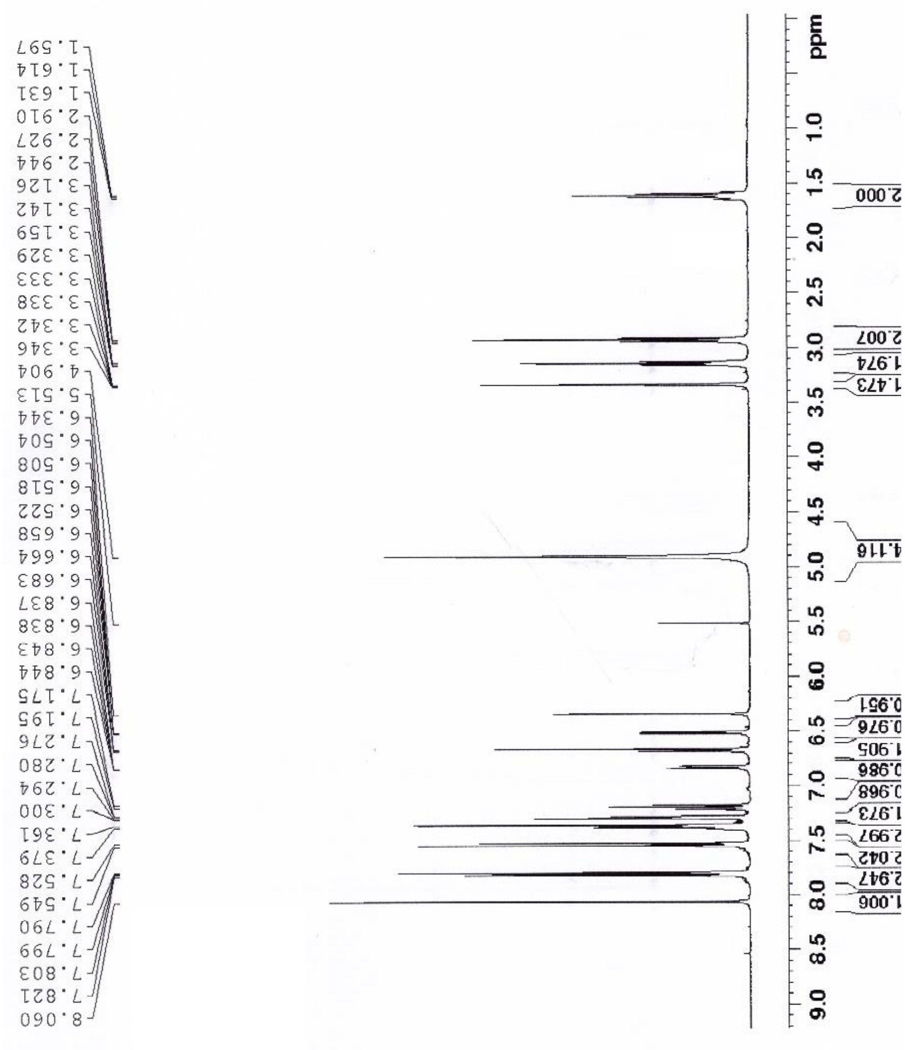
1.597
1.614
1.631
2.910
2.927
2.944
3.126
3.142
3.159
3.329
3.333
3.338
3.342
3.346
4.904
5.513
6.344
6.504
6.508
6.518
6.522
6.658
6.664
6.683
6.837
6.838
6.843
6.844
7.175
7.195
7.276
7.280
7.294
7.300
7.361
7.379
7.528
7.549
7.790
7.799
7.803
7.821
8.060
ppm
1.0
1.5
2.0
2.5
3.0
3.5
4.0
4.5
5.0
5.5
6.0
6.5
7.0
7.5
8.0
8.5
9.0
2.000
2.007
1.974
1.473
4.116
0.951
0.976
1.905
0.986
0.968
1.973
2.997
2.042
2.947
1.006

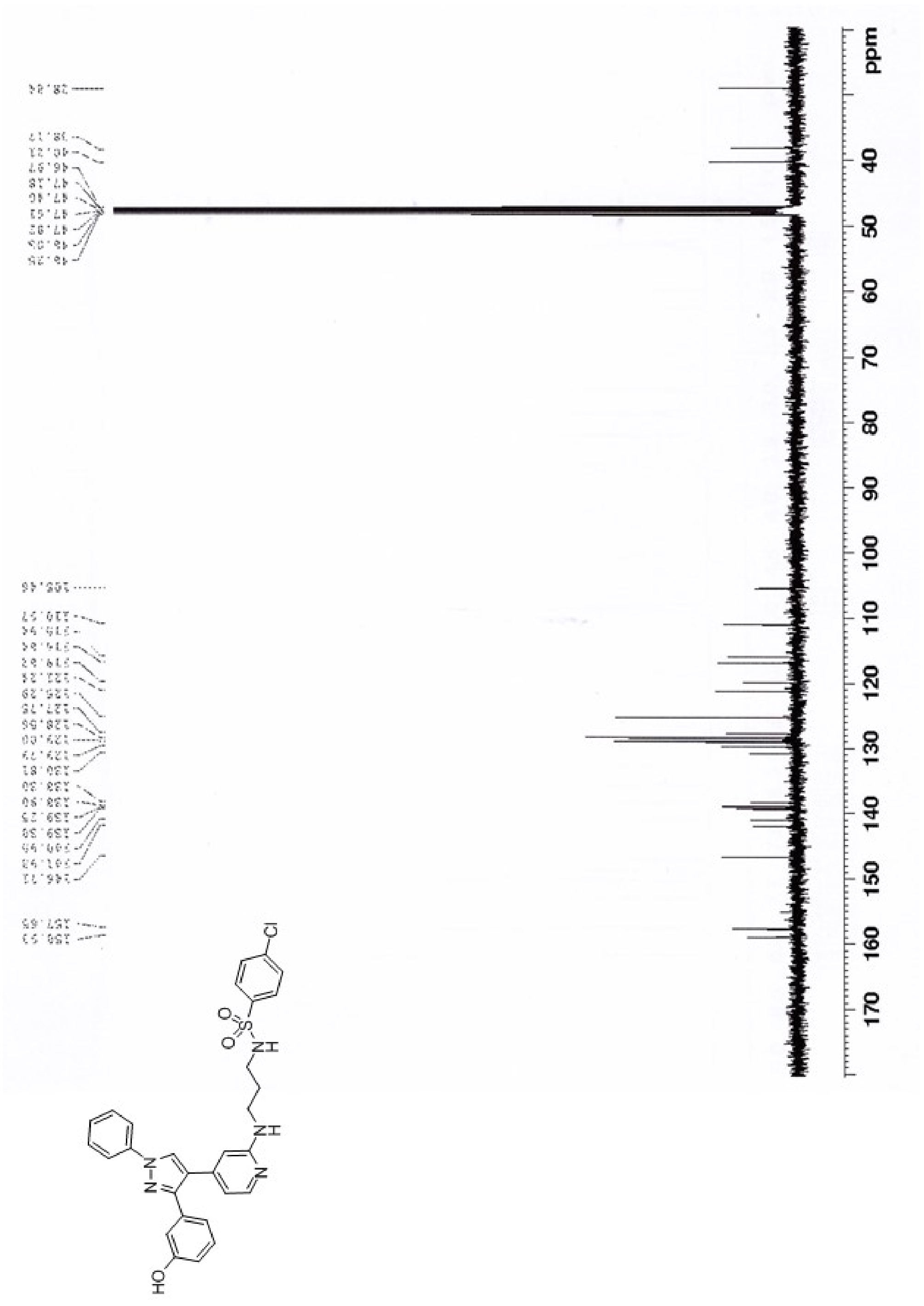

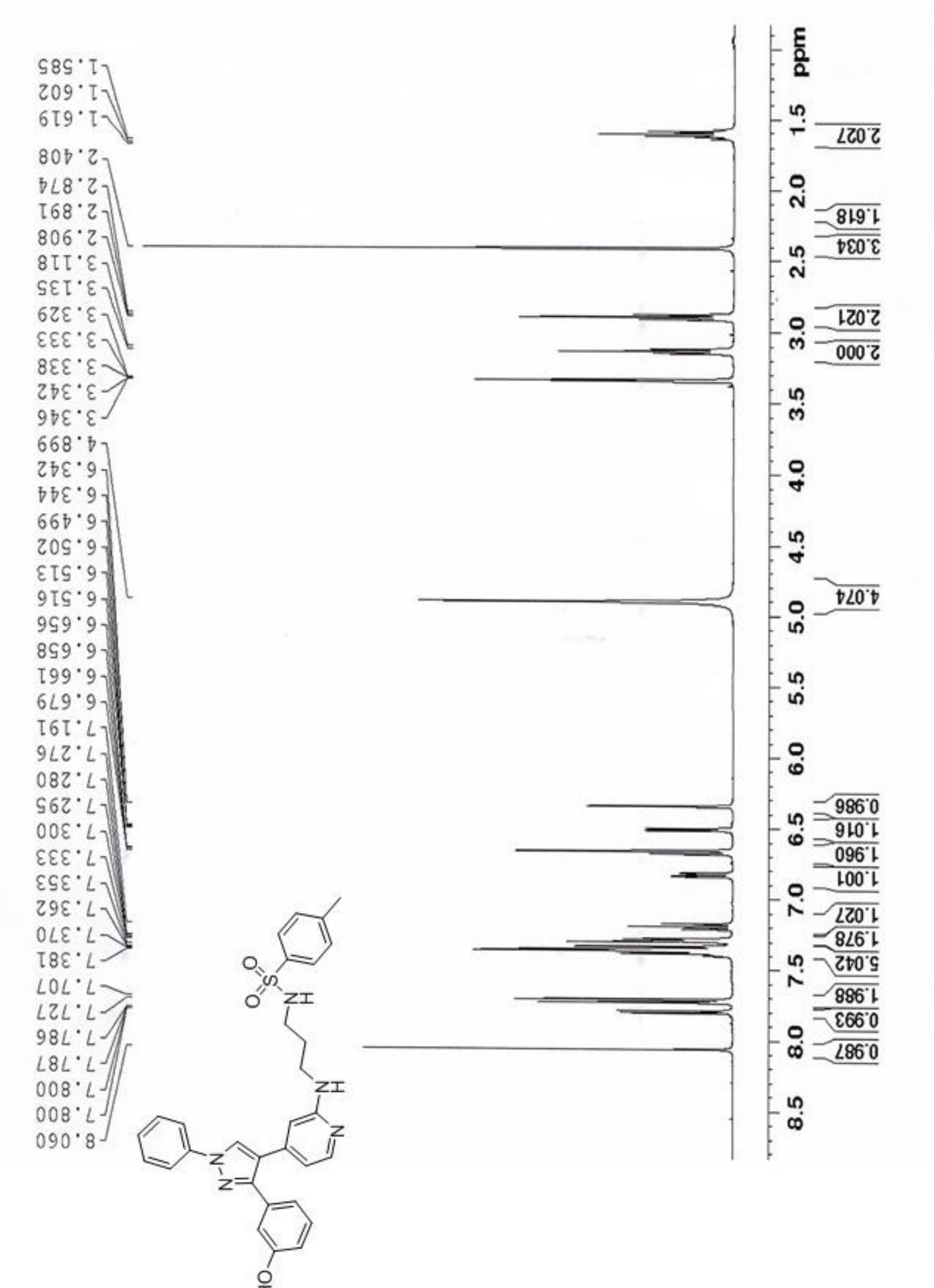

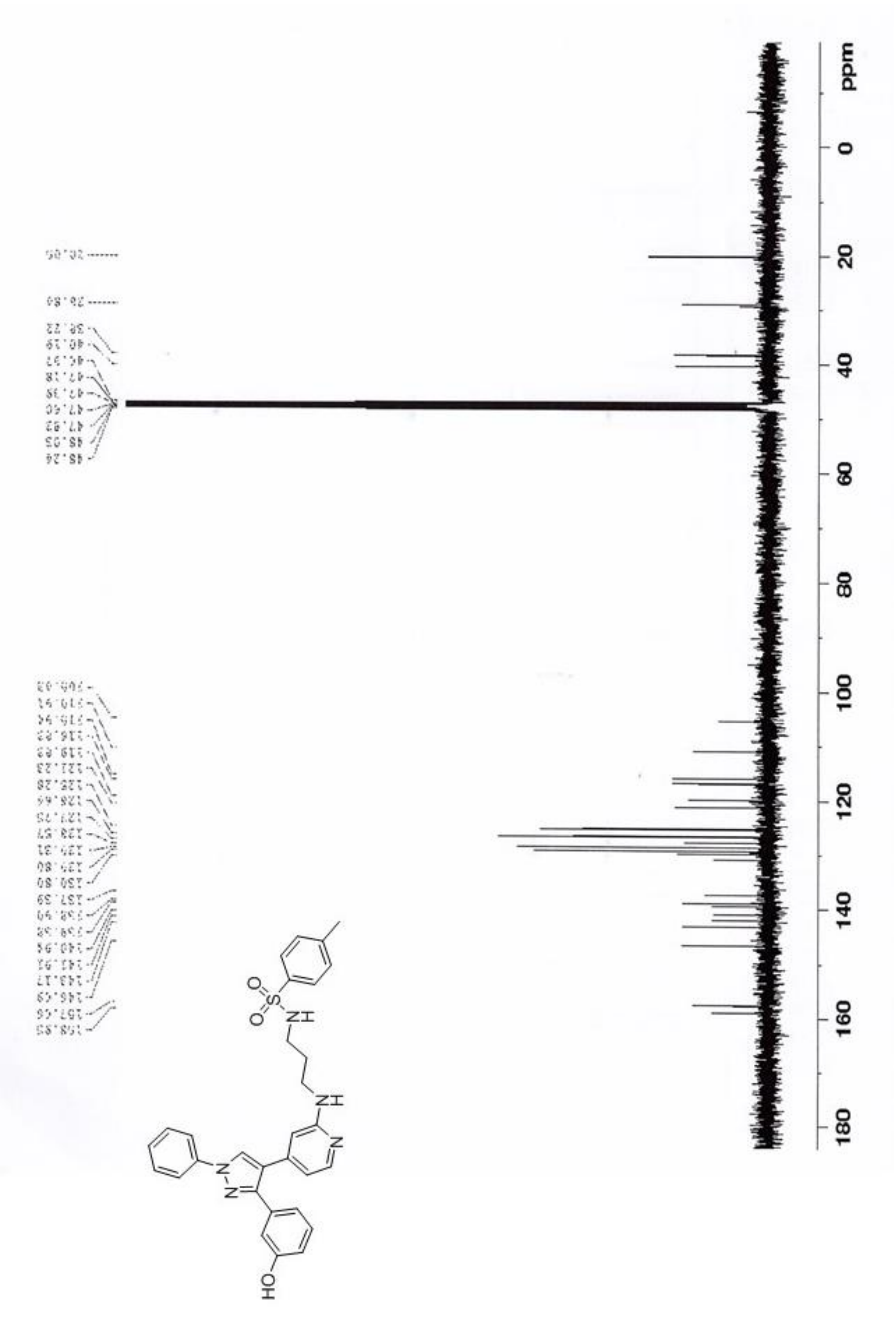

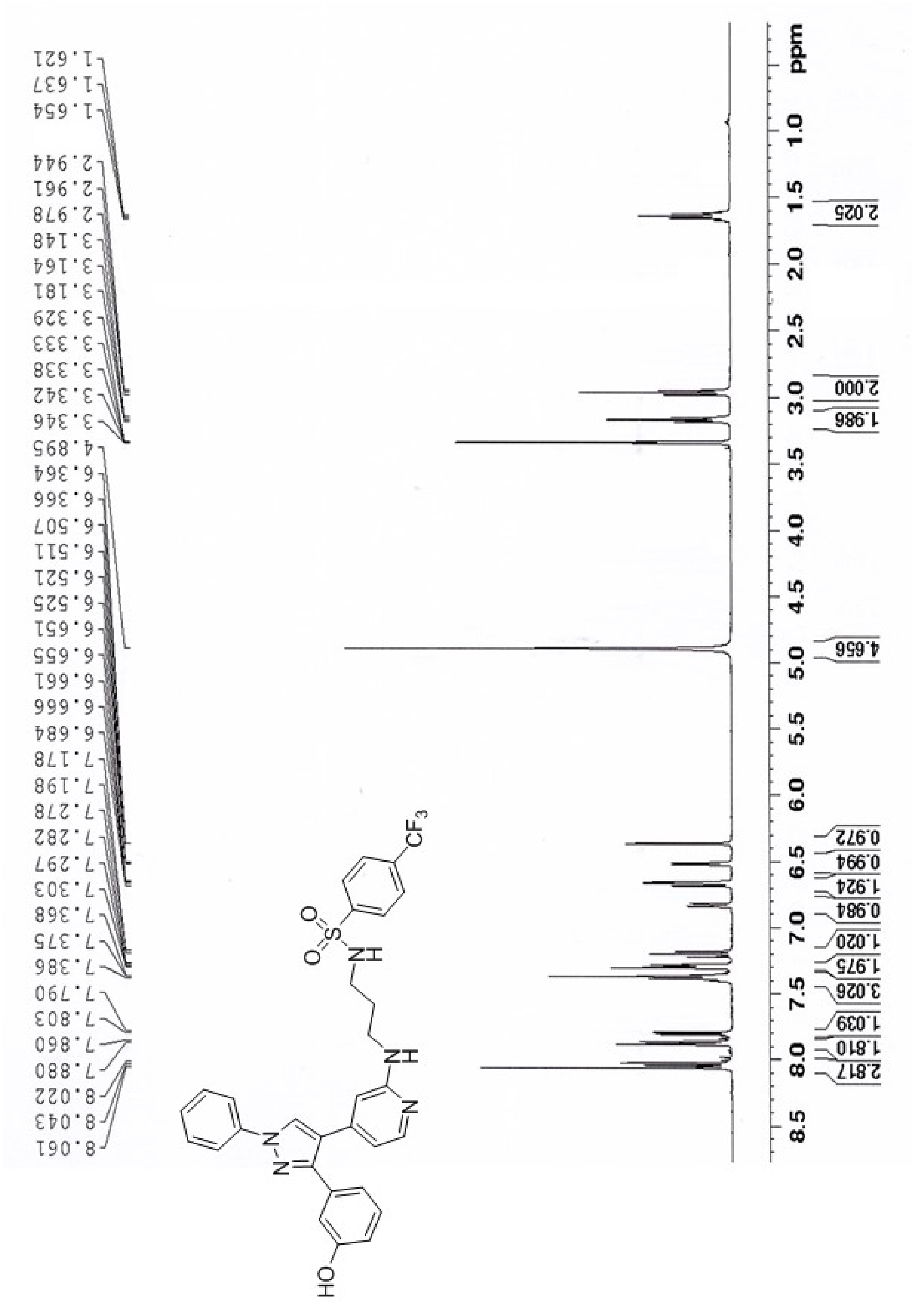
1.621
1.637
1.654
2.944
2.961
2.978
3.148
3.164
3.181
3.329
3.333
3.338
3.342
3.346
4.895
6.364
6.366
6.507
6.511
6.521
6.525
6.651
6.655
6.661
6.666
6.684
7.178
7.198
7.278
7.282
7.297
7.303
7.368
7.375
7.386
7.790
7.803
7.860
7.880
8.022
8.043
8.061
CF_3
HO
ppm
2.025
2.000
1.986
4.656
0.972
0.994
1.924
0.984
1.020
1.975
3.026
1.039
1.810
2.817

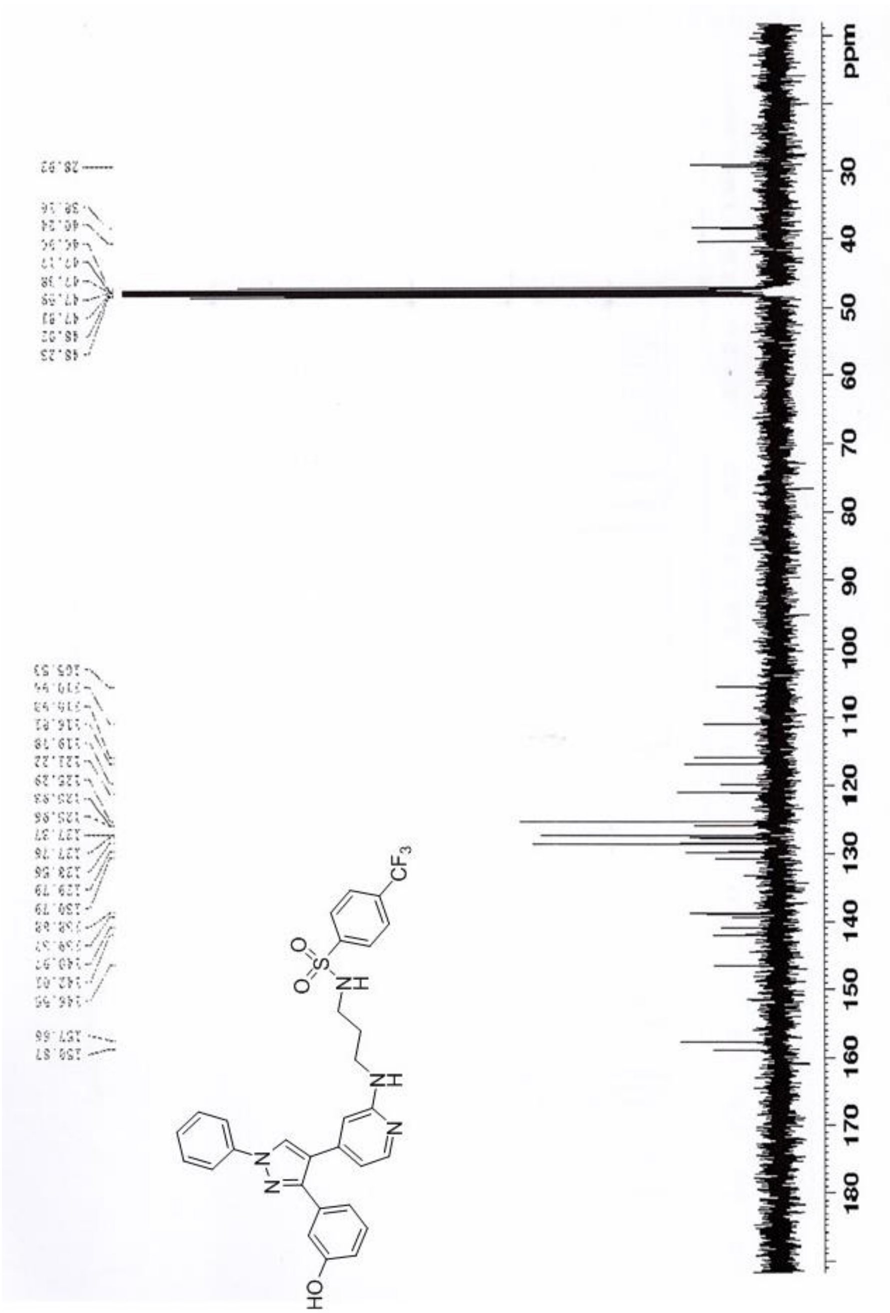

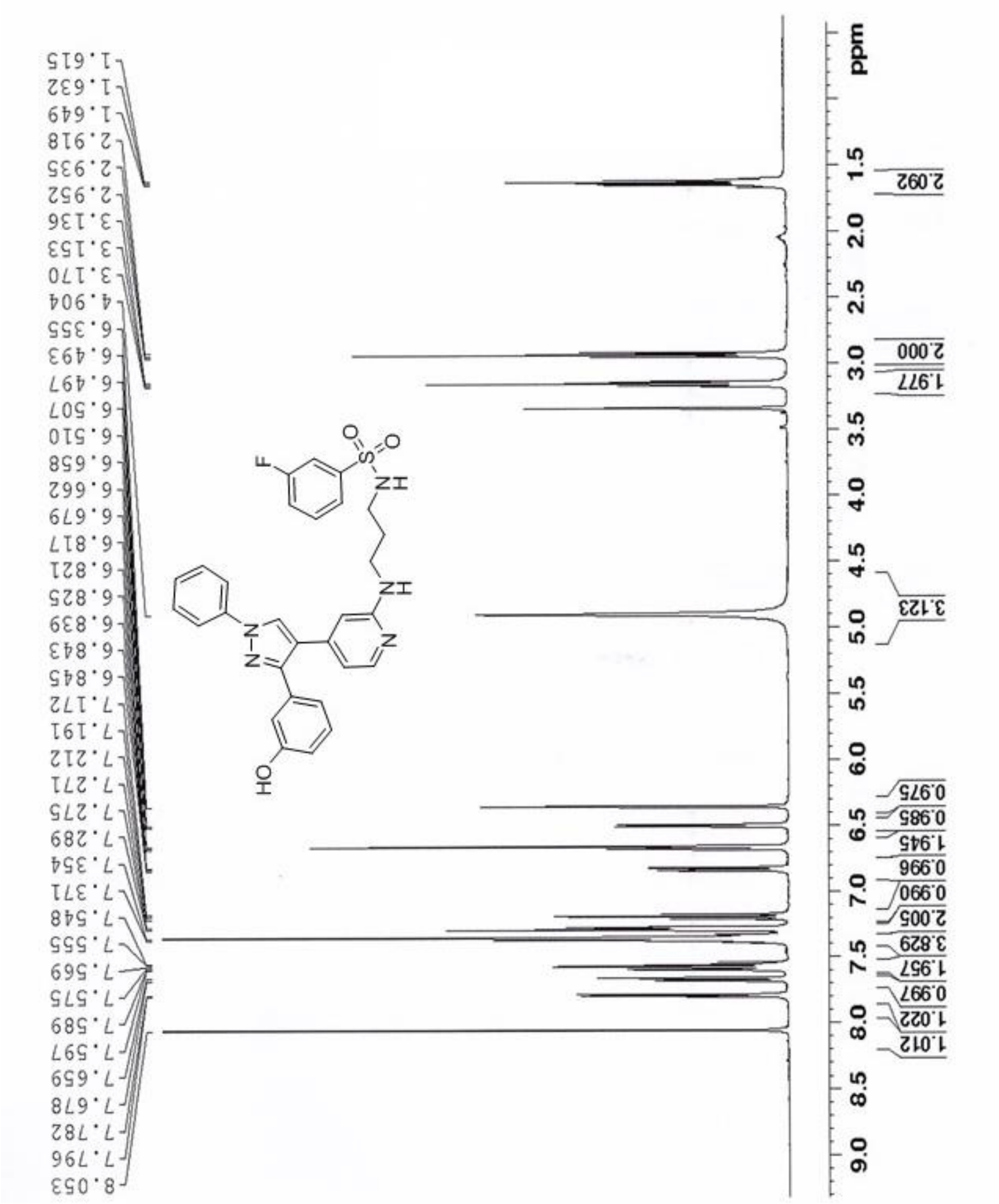
1.615
1.632
1.649
2.918
2.935
2.952
3.136
3.153
3.170
4.904
6.355
6.493
6.497
6.507
6.510
6.658
6.662
6.679
6.817
6.821
6.825
6.839
6.843
6.845
7.172
7.191
7.212
7.271
7.275
7.289
7.354
7.371
7.548
7.555
7.569
7.575
7.589
7.597
7.659
7.678
7.782
7.796
8.053
F
O
S
O
NH
NH
N
N
N
HO
2.092
2.000
1.977
3.123
0.975
0.985
1.945
0.996
0.990
2.005
3.829
1.957
0.997
1.022
1.012
ppm
1.5
2.0
2.5
3.0
3.5
4.0
4.5
5.0
5.5
6.0
6.5
7.0
7.5
8.0
8.5
9.0

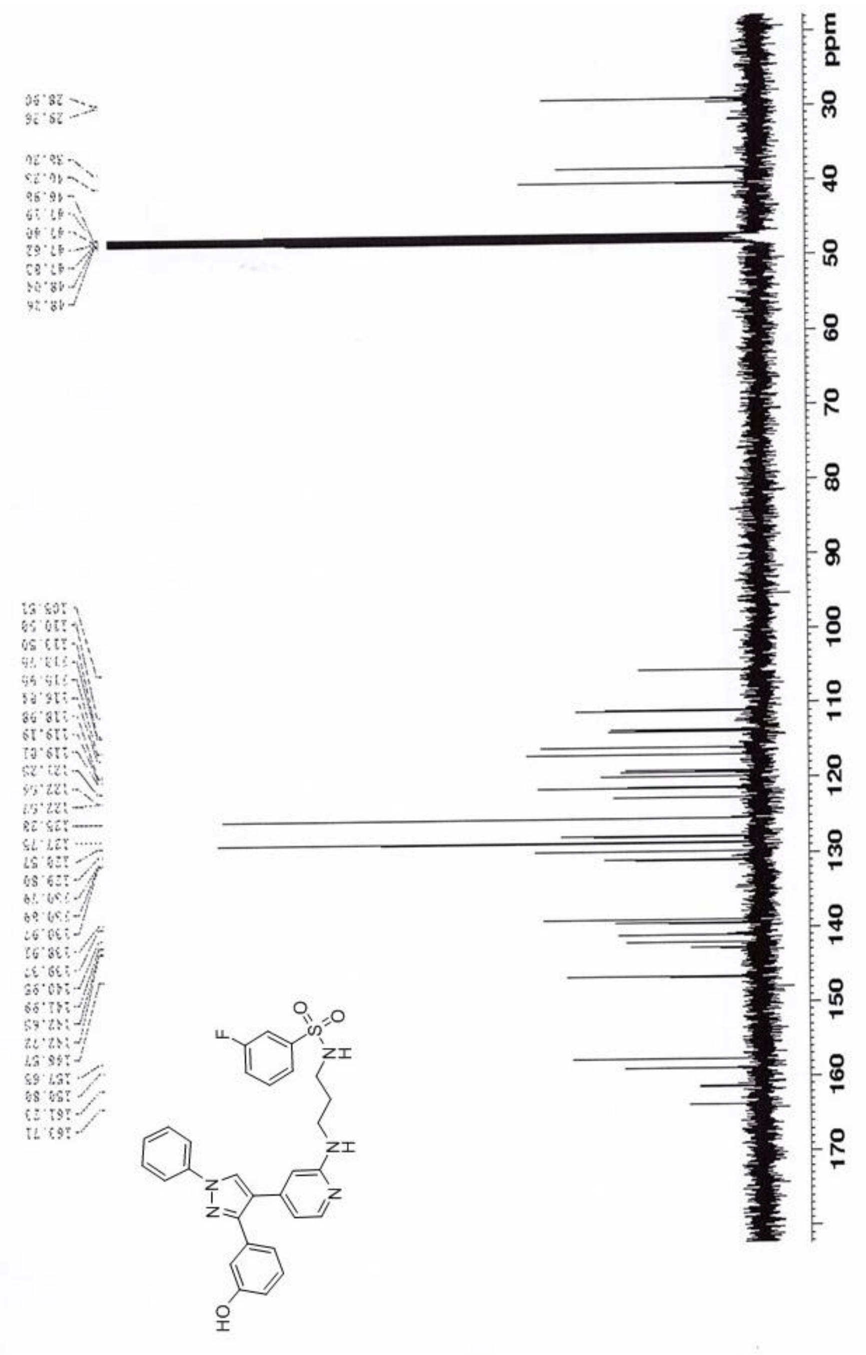

Supplement: Supplementary file 1 [file molecules-23-02556-s001.pdf]
